# Ribosome Pool Engineering Increases Protein Biosynthesis Yields

**DOI:** 10.1021/acscentsci.3c01413

**Published:** 2024-03-20

**Authors:** Camila Kofman, Jessica A. Willi, Ashty S. Karim, Michael C. Jewett

**Affiliations:** † Department of Chemical and Biological Engineering, Northwestern University, Evanston, Illinois 60208, United States; ‡ Department of Bioengineering, Stanford University, Stanford California 94305, United States

## Abstract

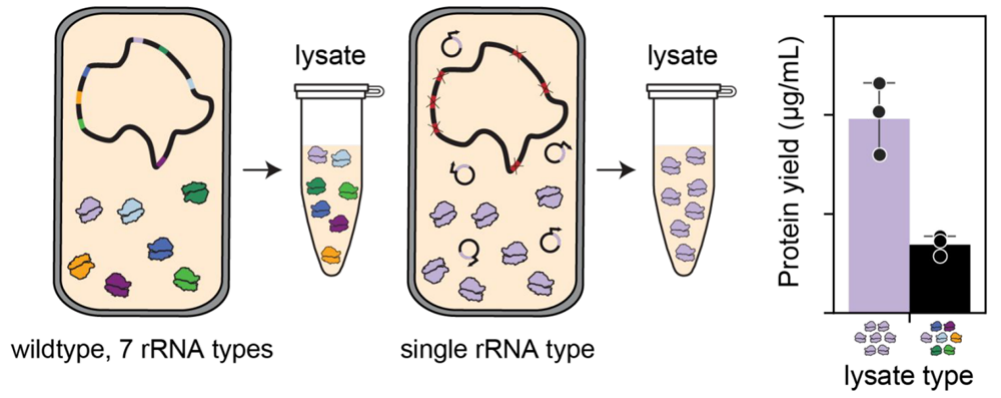

The biosynthetic capability of the bacterial ribosome motivates efforts to understand and harness sequence-optimized versions for synthetic biology. However, functional differences between natively occurring ribosomal RNA (rRNA) operon sequences remain poorly characterized. Here, we use an *in vitro* ribosome synthesis and translation platform to measure protein production capabilities of ribosomes derived from all unique combinations of 16S and 23S rRNAs from seven distinct *Escherichia coli* rRNA operon sequences. We observe that polymorphisms that distinguish native *E*. *coli* rRNA operons lead to significant functional changes in the resulting ribosomes, ranging from negligible or low gene expression to matching the protein production activity of the standard rRNA operon B sequence. We go on to generate strains expressing single rRNA operons and show that not only do some purified *in vivo* expressed homogeneous ribosome pools outperform the wild-type, heterogeneous ribosome pool but also that a crude cell lysate made from the strain expressing only operon A ribosomes shows significant yield increases for a panel of medically and industrially relevant proteins. We anticipate that ribosome pool engineering can be applied as a tool to increase yields across many protein biomanufacturing systems, as well as improve basic understanding of ribosome heterogeneity and evolution.

## Abstract 2

Unique rRNA operons existing in the *Escherichiacoli* genome yield functionally distinct ribosomes with varying recombinant protein synthesis capabilities in cell-free systems.

## Introduction

Ribosomes are macromolecular machines that play a central role in the synthesis of proteins by catalyzing peptide bond formation between amino acids in a sequence defined manner. They are composed of small and large subunits (SSU and LSU) that contain both ribosomal RNA (rRNA) and ribosomal proteins (r-proteins). In *Escherichiacoli*, the 16S rRNA and 21 r-proteins make up the SSU, while the LSU is composed of the 23S rRNA, 5S rRNA, and 33 r-proteins. Ribosomes have conventionally been thought of as uniform molecular assemblies even though most organisms carry multiple copies of unique rRNA-encoding operons (rrn) in their genomes.^1^ The *E*. *coli* K-12 strain MG1655, for example, has seven genomically encoded rRNA operons containing several polymorphisms and are named with letters A–E, G, and H in increasing distance from the origin of replication.^2^

The seven unique rRNA operons in *E*. *coli* have been studied through the lens of promoter strength;^1,3,4^ it is known that the rRNA operon promoters are among the strongest in the genome, responsible for more than 70% of total RNA synthesis in fast-growing cells.^5^ Previous work has shown that certain operons, such as rrnE, have stronger promoters and are more highly expressed.^3^ Other studies have shown that specific rRNA genes, such as the 16S rRNA of rrnH, are more highly expressed in response to nutrient limitation and result in a ribosome population that is more resistant to tetracycline, a class of antibiotics that blocks tRNAs from interacting with the ribosome’s active site.^6^ However, while studying differential transcription of rRNA sequences provides insight into the regulation of ribosome heterogeneity and specialization, it does not directly show the impact of rRNA sequence diversity on the performance of molecular translation. If rRNA sequences produce functionally different ribosomes, then rRNA sequences in the genomes of biomanufacturing strains could be manipulated to express optimized ribosome pools for increasing protein synthesis yields.

Unfortunately, studying if and how native rRNA sequences affect protein translation is limited by our inability to isolate and test ribosomes from specific operons, as well as difficulties in controlling for the effects of operon promoter architecture and position in the genome. Previous work has explored inactivating rRNA operons in the genome to assess how cells performed with fewer ribosomal operons and found that having fewer instances of rRNA in the genome results in slower doubling times, but these findings were not controlled for the differences in genome position and promoter architecture.^7^ An alternative approach to assess the impact of rRNA sequence on ribosome function would be purifying distinct ribosomes from cells. However, adding purification tags with which to isolate specific ribosomes would require genome engineering that is complicated by significant homology between rRNA operons.^8^ In addition, purification tags would only target the SSU or LSU individually rather than the formed 70S particle composed of both subunits, and the tag itself may have confounding effects on translation studies.^9,10^

*In vitro* approaches can circumvent some of the aforementioned limitations and have been used previously to build, assemble, and study known and novel rRNAs.^11−14^ For example, the recently developed *in vitro* ribosome synthesis, assembly, and translation (iSAT) platform provides an approach to individually synthesize and assess the activity of the unique, naturally occurring rRNA operons that exist in the *E*. *coli* genome.^15−17^ Specifically, iSAT enables one-pot coactivation of rRNA transcription, assembly of rRNA with native r-proteins into *E*. *coli* ribosomes, and the synthesis of functional proteins from these ribosomes in a crude S150 extract lacking native ribosomes.^16^ This system allows for the prototyping of different rRNA sequences by simply changing the input DNA that codes for the rRNA of interest. Previously, iSAT has been used to carry out mutation mapping of the 70S ribosome,^14^ enable assessment of computationally designed ribosomes,^18,19^ evolve the ribosome for new function,^20^ and study the assembly landscape of the large ribosomal subunit.^21^

Here, we set out to use the iSAT method to explore whether heterogeneity of native rRNA sequences affects the activity of resulting ribosomes and whether this can be used to optimize protein biosynthesis. We use *in vitro* rRNA prototyping and strain engineering methods to test individual rRNA operons and combinations of operon components. We demonstrate that ribosomes resulting from different operons display a wide range of activities when expressed and assembled both *in vitro* and *in vivo*, and extracts from strains carrying some homogeneous rRNA populations yield significantly improved cell-free protein synthesis over those from the parent strain for a panel of proteins. Our results suggest that ribosome pool engineering has the potential to improve biomanufacturing systems for many applications in synthetic biology, including cell-free protein synthesis and recombinant protein production, as well as to elucidate a deeper understanding of ribosome heterogeneity and evolution.

## Results

### Activity of Ribosomes Derived from Single rRNA Operons Varies Widely

**Figure 1:**
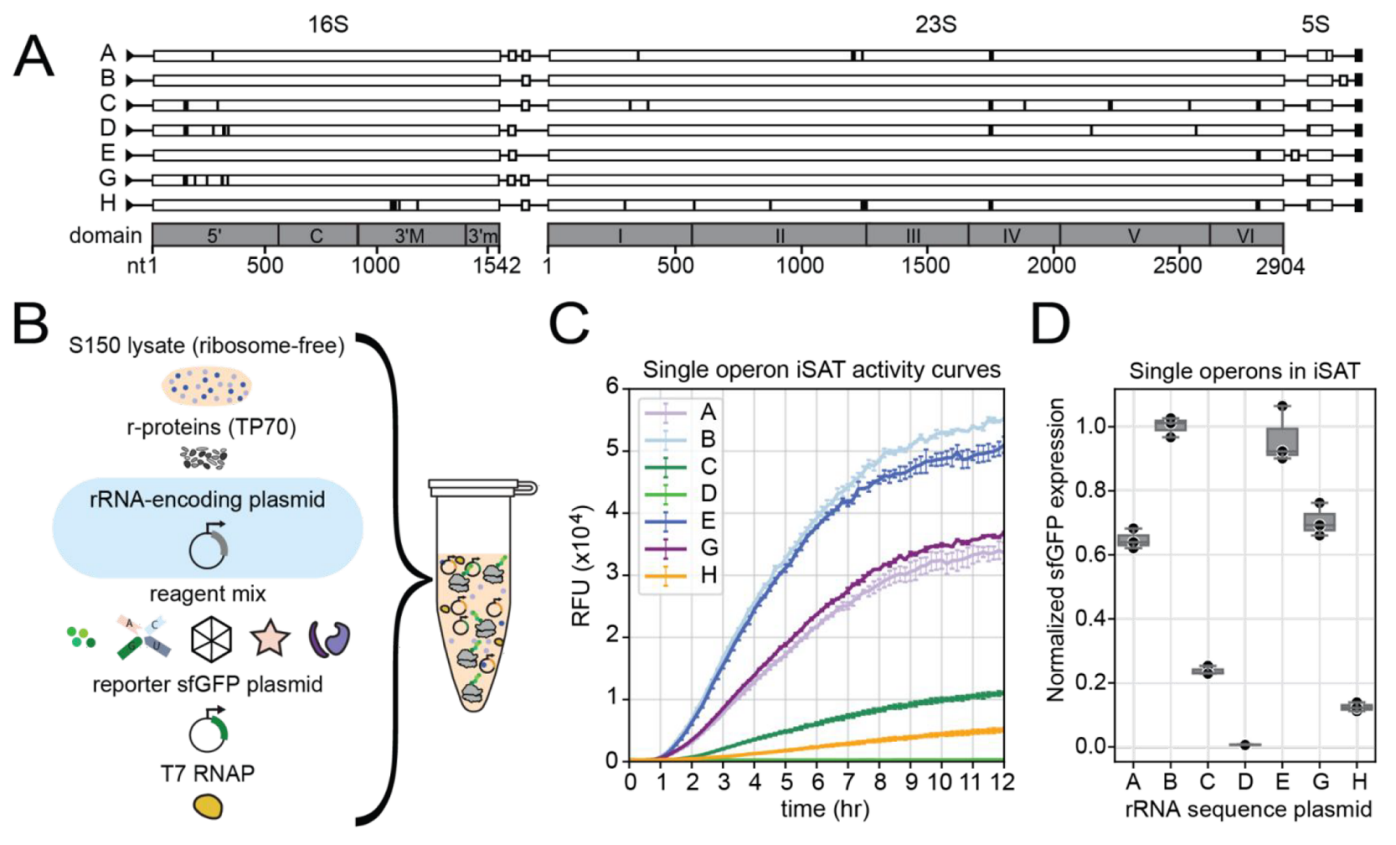
Genomic rRNA operons produce ribosomes that vary in iSAT activity. (A) rrn operons in the genome have different architectures and sequences. Residues that differ from the reference operon B sequence are highlighted in black; white boxes indicate tRNA genes; domains and nucleotide scale are indicated in gray below. (B) iSAT reaction set up allows for expression, assembly, and testing of individual rRNA sequences in a ribosome-free lysate. (C) Single operon rRNA encoding plasmids show a wide range of activities in iSAT. Gene expression curves of sfGFP synthesis in iSAT, mean of *n* = 3. (D) End point of sfGFP expression, normalized to iSAT activity of reference operon B, mean of *n* = 3.

The goal of our work was to characterize the sequence effect of natively occurring rRNA operons on recombinant protein production. As a model, we focused on the seven distinct genomically encoded rRNA operons from *E*. *coli* K-12 strain MG1655 (Figure 1A). These operons are largely the same in sequence but differ by a total of 21 unique point mutants in the 16S rRNA, 34 in the 23S rRNA, and 3 in the 5S rRNA. These mutations are present in all domains of the 23S rRNA and exist on both the 5′ and 3′ ends of the 16S rRNA. Notably, the operons even have sequence differences in the 23S rRNA that forms the catalytic active site of the ribosome, or peptidyl-transferase center (PTC).

We used the iSAT platform^16^ to study how the rRNA operon sequence differences impacted protein production. Typically, in the field of ribosome engineering and in past work using the iSAT system, the model operon rrnB is used.^12,22,23^ Thus, we used the architecture of the pT7rrnB plasmid (Supplementary Table S1) as a template, and rRNA fragments from other operons were exchanged into this plasmid (Supplementary Tables S2, S3). With each distinct operon on individual plasmids, we assembled separate iSAT reactions for each operon supplementing a ribosome-free S150 lysate with the operon plasmid, T7-superfolder green fluorescent protein (sfGFP) plasmid (reporter), ribosomal proteins, and energy mix (Figure 1B).^18^ Notably, iSAT has two features that help to prevent rRNA and ribosome degradation. First, the strain that we use to make S150 lysate lacks RNase I and, thus, is known for its low RNase activity. Second, during S150 extract preparation, RNase inhibitor is added both before and after cell lysis, as described in the Methods section.

iSAT reactions were incubated at 37 °C where the transcribed rRNA is assembled into a ribosome and tested for the ability to synthesize sfGFP. We found the seven naturally occurring rRNA operons in *E*. *coli* produce ribosomes exhibiting a wide range of protein synthesis activity. Operons B and E (16S:23S:5S) yielded the highest amount of sfGFP, and operons D and H yielded the lowest amount (Figure 1C,D), with operon D being nonfunctional.

**Figure 2:**
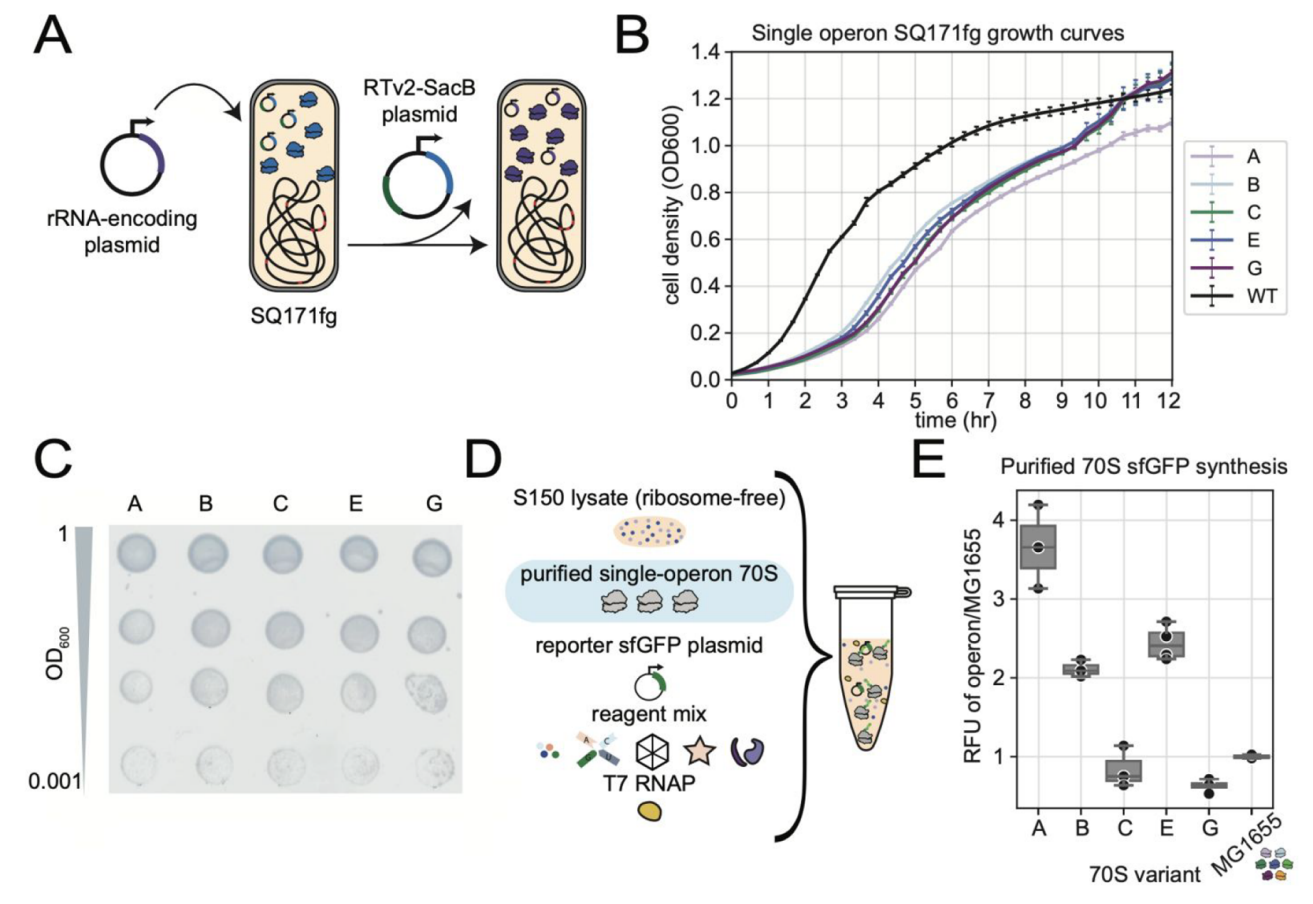
Expression and assembly of single-operon ribosomes *in vivo* shows advantages over native heterogeneous ribosome pool. (A) Single-operon strain selection process. Operon of interest is transformed into SQ171fg cells to replace the original rRNA copy, which is maintained on a SacB-containing plasmid. (B) Growth curves of a wild type *E*. *coli* strain (BL21 Star (DE3)) and cured SQ171fg strains carrying single operons. Cell density was measured in LB medium at 37 °C. Curves represent mean and standard deviation of up to *n* = 7 replicates. (C) SQ171fg strains carrying single operons A, B, C, E, and G were successfully generated and grown on agar plates. Single-operon strains were normalized to OD_600_ = 1, and serial dilutions were spotted onto plates. Plates were imaged when the most dilute sample showed cell growth. Images are representative of *n* = 3 assays. (D) 70S ribosomes purified from single-operon strains can be tested for sfGFP production in a ribosome-free lysate. (E) Purified 70S show a wide range of activity, with many variants showing an advantage over the mixed pool (MG1655). End point of sfGFP expression, normalized to iSAT activity of reference MG1655 pool, mean of *n* = 3.

We next investigated whether the functional variation of ribosomal operons is also observed in living *E*. *coli* by constructing strains that express only one rRNA operon sequence instead of the 7 native sequences. This process involves transforming rRNA-carrying plasmids into strain SQ171fg, which was evolved from the “Squires” SQ171 strain.^23,24^ The SQ171fg has all 7 genomic rRNA copies removed and survives off an rRNA sequence encoding a tethered ribosome, Ribo-T v2,^23^ on a plasmid. This Ribo-T v2 plasmid also contains SacB and an antibiotic resistance gene, which can both be used as selection markers (Figure 2A).^25^ If the rRNA variant of interest is able to support cell growth, the original plasmid can be cured, replacing the original RT-v2-SacB plasmid with a plasmid carrying the rRNA variant of interest.^18^

Operons A, B, C, E, and G were successfully transformed and selected, having similar growth phenotypes when grown in LB medium (Figure 2B,C). While these SQ171fg derived single-operon strains could grow under the laboratory conditions tested, they had slower growth rates and increased lag times as compared to wild type strains, such as MG1655 and BL21 Star (DE3) (Supplementary Table S5). This can be attributed, in part, to the metabolic burden of plasmid maintenance as has been reported in the literature.^26,27^ Single operon strain development for operons D and H was not successful, as the original SacB plasmid was not able to be cured, indicating that rrnD and H sequences are unable to independently enable cell growth in the context of the SQ171g strain under controlled laboratory conditions.

We then compared the translational activity of ribosomes derived from each operon to a wild-type heterogeneous pool of ribosomes. 70S ribosomes were purified from the single-operon strains A, B, C, E, and G, as well as from the parent strain MG1655, which natively expresses all seven rRNA operons. Reactions were prepared *in vitro* with purified ribosomes, ribosome-free lysate, reporter plasmid, and reagent mix (Figure 2D). Operon A, B, and E ribosomes performed better than the MG1655 70S ribosomes, while C and G showed lower sfGFP production than did the MG1655 pool (Figure 2E, Supplementary Figure S2). This, in combination with the failure of D and H to enable cell growth in the context of the SQ171fg strain, suggests that the MG1655 70S translation capacity may be diluted by less active variants, specifically C, D, G, and H. Replacing weaker variants with the most active ribosomes could optimize the overall ribosome pool and enable higher efficiency protein production systems.

Of note, we see differences between growth trends (Figure 2B) and purified ribosome activity (Figure 2E), indicating that a strain’s growth profile is not necessarily correlated to the protein synthesis capacity of its ribosome pool. For example, strains carrying operon A have similar growth profiles to strains carrying operon C (Figure 2B), while the purified A ribosomes produce nearly 4-fold higher sfGFP yields when tested in the cell-free translation (Figure 2E). Such variations are consistent with previous studies that have shown that activity of a specific rRNA sequence in an *in vitro* context does not always correlate directly to that same ribosome’s activity *in vivo*.^18,19^ These observations could be attributed to differences between testing for protein synthesis activity in simplified, cell-free environments versus assessing a ribosome pool’s ability to enable cell proliferation.

As expected, we also observed that the activity trends for purified homogeneous ribosome pools (Figure 2E) do not perfectly match the trends seen in iSAT (Figure 1C) (see, for example, operons A and G). This apparent inconsistency is a likely result of the assay differences. Whereas iSAT activity reports on combined *in vitro* rRNA transcription, ribosome assembly, and translation, cell-free reactions with purified ribosomes are only assessing translational activity of ribosomes expressed and assembled in cells and then purified via ultracentrifugation.

### Removing rRNA Polymorphisms Recovers Translation Activity

To investigate why *E*. *coli* might maintain copies of rRNA operons that yield low-activity ribosomes such as those from D and H, we designed an experiment to ask whether pairing the SSU and LSU rRNAs from different operons could recover the activity of operons that were less active. In living cells, it is possible that the SSU from one operon could associate with an LSU from a different operon to form a 70S ribosome because translation of a messenger-RNA (mRNA) is initiated first by the association of an SSU with the 5′ end of the mRNA, followed by the LSU coassociating to form a translationally competent complex.^28^

**Figure 3:**
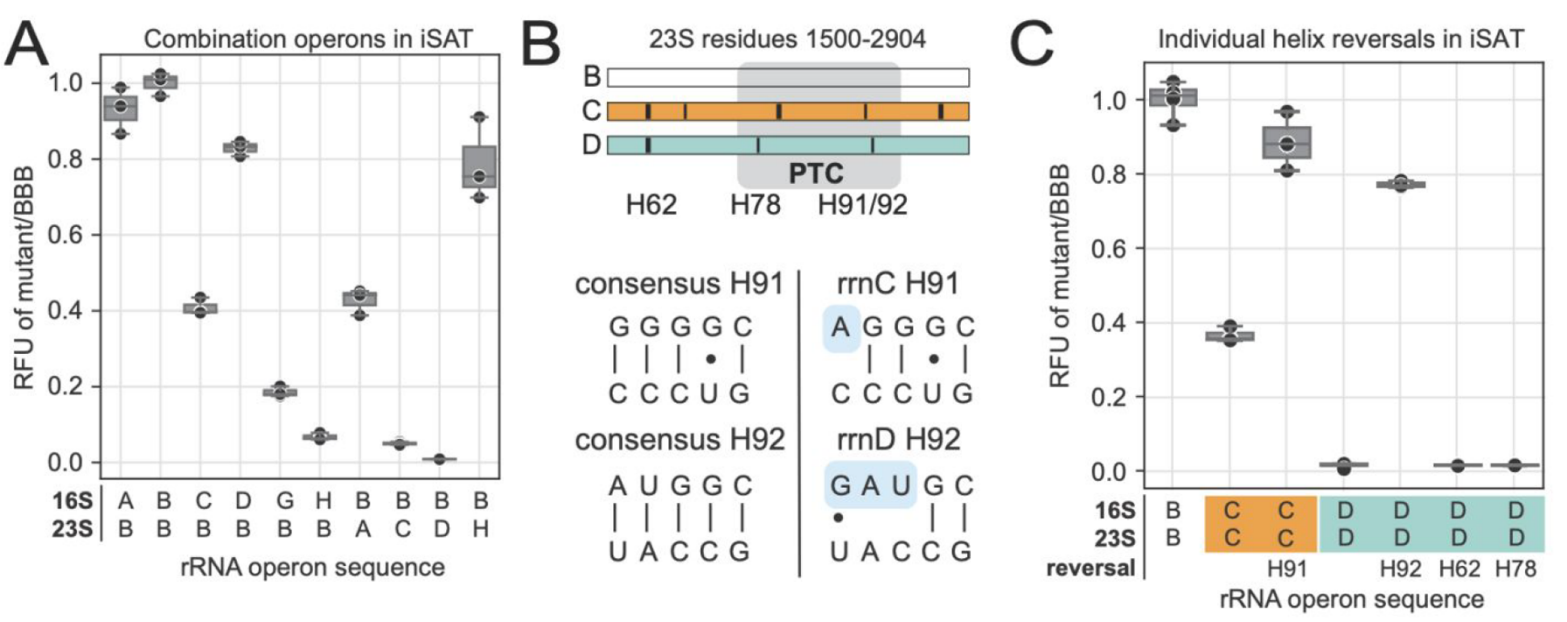
Engineered ribosomal operons highlight that some natively occurring rRNA sequences are low performing or inactive. (A) iSAT activities of the combination operons. End point of sfGFP expression, normalized to iSAT activity of reference BBB, mean of *n* = 3. (B) Helix 91 (H91) and 92 (H92) polymorphism case study to reverse nonconsensus sequences in the PTC. (C) Reversing H91 and H92 polymorphisms to match the consensus helps recover activity of operons C and D in iSAT. End point of sfGFP expression, normalized to iSAT activity of reference BBB, mean of *n* = 3.

To build operon combinations, we made constructs that used 16S and 23S rRNA genes individually from each operon with counterparts from operon B to test in iSAT. All constructs carry the 5S rRNA sequence from the model B operon. As some of the 16S and 23S rRNA sequences are the same between different operons, this resulted in only 10 unique sequences, including that of the B operon (e.g., the 23S rRNA sequence of the B operon matches that of the G operon). When the unique combination constructs were tested for sfGFP production in iSAT, we found that 6 of 9 pairs yielded ribosomes that produced at least 20% as much sfGFP as the control from operon B (Figure 3A). However, the 23S rRNA of operons C and D yielded a significant decrease in activity when paired with the 16S rRNA from operon B. Similarly, the 16S rRNA sequence from operon H resulted in a severe drop in the level of sfGFP production. These results indicate that polymorphisms in individual subunit rRNAs may be responsible for the negative impact on the translation ability.

Considering the structure of the rRNA and where these polymorphisms fall in 3D space, we can infer how sequence differences between operons may affect activity (Supplementary Figure S1). While most of the polymorphisms sit on the outer regions of the rRNA and are thus less likely to be directly interacting with translocation processes of tRNAs through the ribosomal active site, some exist in motifs that are known to play dynamic roles in translation activity. For example, the 23S rRNA sequences of operons A, C, E, and H differ from that of B, D, and G at Helix 98 (H98), a motif that has been shown to participate in a tertiary interaction that is important for ribosome stabilization.^29^ Similarly, H68, a 23S rRNA helix that is extended by a Watson–Crick (WC) base pair in the A operon is known to be actively involved in dynamic ribosome movements that are necessary for the process of elongation via coordination with the L1 stalk and tRNAs.^30^ The additional WC base pair, which would increase the length of H68, potentially affects the dynamics of the interaction with the L1 stalk, altering the efficiency of the elongation process. In the 16S rRNA, operon H carries seven unique polymorphisms in helix 33 (h33), which sits in the “head” of the small subunit and plays an important role in ratcheting along the mRNA in the process of translation.^31^ Additionally, many polymorphisms exist in rRNA motifs that interact closely with r-proteins. For example, h11 of operon D contains a single residue change, but is adjacent to S16, a protein that is essential for cellular viability.^32^ Changes in the sequence of h11 could thus impact the interaction with S16 and potentially have deleterious effects on the ribosome activity.

The 23S rRNA sequences from both operons C and D carry polymorphisms in the PTC that differentiate them from consensus operon B (Figure 3B). The PTC, located in domain V of the rRNA, is one of the most sequence conserved and catalytically important regions of rRNA.^33^ Operon C contains a single nucleotide polymorphism in Helix 91 (H91) resulting in the loss of a WC base pair, which is the strongest possible RNA base pair interaction.^34^ Similarly, operon D contains a deletion and a single nucleotide polymorphism in Helix 92 (H92), effectively removing two of the three WC base pairs present in the consensus sequence (Figure 3B). Both helices H91 and H92 compose part of the functionally important and highly sequence conserved region of the “accommodation corridor”.^35^ Additionally, our previous work has shown these two helices to be highly sensitive to mutations, especially when mutations result in a loss of WC base pairing interactions.^18^

To test whether these specific polymorphisms are the source of decreased ribosome activity, we synthesized individual plasmids containing rrnC and rrnD with single motifs of interest mutated to match the consensus sequence of operon B and tested them in iSAT (Figure 3C). We found that the resulting ribosome activity more than doubled when the single nucleotide polymorphism in H91 of operon C was reversed to that of the Operon B sequence. The activity of operon D changed from being undetectable to achieving nearly 80% of operon B’s activity when the polymorphisms in H92 were reversed to the sequence of rrnB. We then reverted other polymorphisms in operon D (in H62 and H78) but did not observe activity from these ribosomes. These data indicate that the mutations of helices H91 and H92 are largely responsible for the decrease in ribosome activity from operons C and D, suggesting that optimization of ribosome pools (i.e., removing these low-activity rRNA sequences and replacing them with high performing variants) could improve protein production capacity in *E*. *coli*. Future work to systematically elucidate all effects of individual and combinatorial polymorphisms on translational activity would improve our understanding of the mechanistic and functional consequences of these distinct, natively occurring rRNA sequences.

### Single Operon Derived Ribosomes Pools Increase Protein Biosynthesis Yields

**Figure 4:**
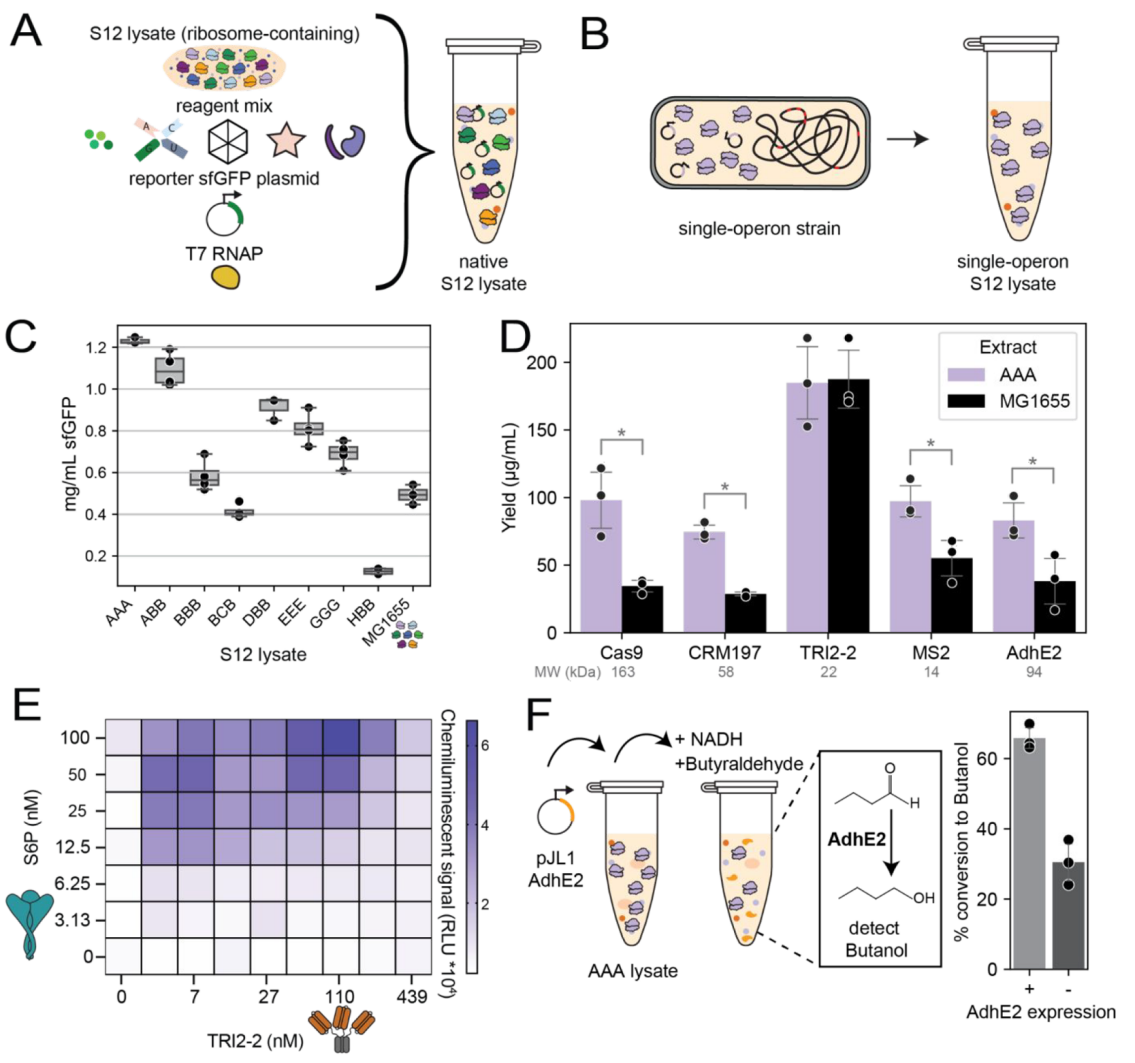
Homogeneous ribosome pools increase protein biosynthesis yields relative to heterogeneous ribosome pools. (A) Standard S12 lysates for CFPS contain a heterogeneous ribosome pool. (B) S12 lysates for CFPS made from single-operon strains yield lysate expressing a homogeneous ribosome pool. (C) Homogeneous ribosome pool lysates show a wide range of sfGFP production, and some outperform standard S12 lysate from a mixed ribosome pool (MG1655). Operon sequence notation shown as 16S:23S:5S. Error bars indicate standard deviations of *n* = 4 replicates. (D) Protein yields as determined by radioactive quantification of ^14^C-leucine for a panel of proteins. Standard deviation shown for *n* = 3 replicates. Statistical significance in expression between AAA and MG1655 lysate denoted by asterisk (*) as calculated by a student’s paired *t* test with *p* < 0.05. (E) AlphaLISA binding pattern of TRI2-2 interfacing with S6P. (F) AdhE2 expressed in AAA lysate shows expected butyraldehyde conversion efficiency. Butanol produced in the absence of AdhE2 expression is a result of native *E. coli* alcohol dehydrogenases. Standard deviation shown for *n* = 3 replicates.

We next sought to use sequence-optimized ribosomes to increase the protein biosynthesis yields. As a model, we explored this strategy in the context of cell-free protein synthesis (CFPS). CFPS is an attractive approach to produce proteins *in vitro* without the need to maintain cell growth.^36,37^ In recent years, CFPS has matured to impact a variety of applications in diagnostics, biomanufacturing, and educational kits, among others.^38−48^ Typically, the ribosome-containing lysates (S12 lysates) for CFPS are made from bacterial strains harboring multiple rRNA operons, producing a lysate with a heterogeneous ribosome pool (Figure 4A). Here, we sought to assess if protein synthesis could be increased by creating CFPS-capable lysates that do not contain ribosomes derived from these deleterious operons (e.g., operon D).

We made cell-free lysates derived from source strains with homogeneous ribosome pools (i.e., expressing single rRNA operons) (Figure 2A; Figure 4B). We chose single operons that produced ribosomes with a relative activity over 50% in iSAT (Figure 1C; Figure 3A) as well as two low-performing operons (BCB and HBB, notation 16S:23S:5S rRNA) as negative controls. Of note, sequence BHB, which performed well in iSAT, was unable to enable cell growth in the context of SQ171fg and so could not be prepared as a single-operon lysate. We then set up a CFPS reaction with these lysates and measured sfGFP production (Figure 4C). We found that single operon lysates produced more protein than lysates made from the parent strain (MG1655) with a pool of 7 operons, and the lysate containing only operon A rRNA (AAA) showed nearly a 3-fold increase in protein production. Notably, activity trends seen in the *in vivo* purified ribosomes closely match those of the CFPS reactions made from the single-operon strains (compare Figure 2C and Supplementary Figure S2 to Figure 4C).

We then took the highest performing lysate (AAA) and used it to express a panel of five proteins that differ in size, function, and structure (Figure 4D). We chose proteins representing diverse fields of interest for industrial and medical applications (e.g., genetic engineering (Cas9), vaccines (CRM197), antibodies/protein binders (TRI2-2), bacteriophages (MS2), and sustainable chemical production (AdhE2) (Supplementary Table S4)). Of the five proteins tested, four showed a statistically significant increase in yield (as calculated by a student’s paired *t* test with *p* < 0.05), and one had comparable expression when expressed in the AAA lysate compared to the MG1655 lysate.

We next assayed two of the proteins for activity. We used an amplified luminescent proximity homogeneous assay (AlphaLISA)^49^ to detect the binding of TRI2-2, a multivalent minibinder protein, to the trimeric HexaPro SARS-CoV-2 S glycoprotein (S6P)^43^ (Figure 4E). AlphaLISA detected a characteristic binding interaction between CFPS-expressed minibinder TRI2-2 and target S6P. We also confirmed the functionality of the aldehyde-alcohol dehydrogenase (AdhE2) from *Clostridium acetobutylicum* by measuring conversion of butyraldehyde to butanol in crude AAA lysates with and without AdhE2 expression (Figure 4F).^50^ When AdhE2 was expressed in AAA lysate, we measured a net conversion rate of ∼35% of butyraldehyde to butanol, matching previously reported values.^50^ The butanol yield seen in the negative control (without AdhE2 expression) results from the previously described activity of native *E*. *coli* alcohol dehydrogenases which act on butyraldehyde.^51^ Notably, attaining an equivalent butanol yield using MG1655 lysate to express AdhE2 required more than twice the CFPS reaction volume (Supplementary Figure S3). These findings highlight that using a lysate for CFPS containing only AAA rRNA sequences is beneficial for significantly improving the functional yields of a wide variety of proteins.

## Discussion

We set out to investigate the impact of sequence differences in rRNA operons found in nature on protein biosynthesis. While past works have indicated that ribosomal operons in *E*. *coli* are differentially transcribed and that trends in transcription can change as a function of environmental stresses,^3,6^ changes in translation activity arising from unique operon sequences have not been directly studied, to our knowledge. By using an *in vitro* ribosome synthesis, assembly, and translation system, we were able to determine for the first time that native rRNA sequence heterogeneity results in significant protein synthesis differences from the resulting ribosomes. In fact, some operons, like rrnD and rrnH, have no or little activity in iSAT and are unable to independently support cell growth in the context of SQ171fg, while others outperform the natively expressed 7-operon mixture. By leveraging these findings, we were then able to show proof-of-concept that cell-free systems composed of homogeneous ribosome pools derived from high-performing single rRNA operons yield a statistically significant increase in expression of a variety of proteins when compared with lysates expressing the wild-type, heterogeneous ribosome pool of wild-type ribosomes.

Our finding that ribosomes derived from the rrnD and rrnH operons were nonfunctional was surprising and suggests that the native *E*. *coli* ribosome pool may be diluted with low-performing variants such as D and H. This begs the question of why the *E*. *coli* genome would retain ribosome variants of lesser fitness; heterologous ribosomes with suboptimal translation performance may contribute to cell survival in adverse conditions, such as being able to translate during nutrient starvation^6^ or to fine-tune translation while entering and exiting stationary phase hibernation.^42^ It may be that laboratory cell growth conditions provide a non-natural environment where some ribosome variants perform better, while others do not contribute to high protein yields, as was measured in this study. Further investigation of these specialized ribosomes’ properties is needed.

In summary, this work demonstrates new basic science knowledge that functional activity variation exists across ribosomes derived from the seven rRNA operons in natively expressed ribosome pools in *E*. *coli*. We also illustrate the concept of ribosome pool engineering and show that some rRNA sequences have increased bulk protein biosynthesis yields. We anticipate that ribosome pool engineering will enable new applications in common workhorse organisms and strains by optimizing the ribosome pool to contain only the most productive rRNA sequences for specific objectives both *in vitro* and *in vivo*. Thus, ribosome pool engineering represents a previously overlooked dimension of optimization for maximizing industrial protein production. Looking forward, we anticipate that studying rRNA sequence-function relationships will build a deeper understanding of how ribosomes have evolved and how we might design specialized ribosomes for biotechnology and synthetic biology.

## Methods

### Plasmids

Ribosomal operon sequences and annotations were acquired from the *Escherichia coli* K-12 substr. MG1655 reference genome (EcoCyc). rRNA-coding plasmids were constructed by mixing and matching fragments from synthetic plasmids ordered from Twist Biosciences within a pT7rrnB backbone as previously described.^18^ As some rRNA sequences between different operons match (for example, operons E and B have identical 23S rRNA sequences), only 12 total rRNA constructs were purchased: ABB, BEB, CBB, DBB, GBB, HBB, BAB, HBB, BCB, BDB, BEB, and BHB (16S:23S:5S). Primers were designed to amplify the 16S and 23S rRNA sequences from the sequence-verified Twist plasmids and combined into the AAB/BBB/CCB/DDB/EEB/GGB/HHB sequences as well as the mixed-operon 16S and 23S rRNA combination constructs using Gibson assembly. 5S polymorphisms were introduced via site-directed mutagenesis to result in pure-operon sequences AAA/BBB/CCC/DDD/EEE/GGG/HHH and confirmed by Sanger sequencing. Plasmids were cloned into chemically competent Dh10B and purified using the Zymo Midiprep Kit and then further purified via ethanol precipitation using 0.5 M NH_4_OAc for use in iSAT reactions.

Plasmids for expression of rRNA *in vivo* were assembled by cloning the rRNA sequence from the Twist plasmids and using Gibson assembly to insert it into a pAM-backbone plasmid, so that the rRNA expression is under the control of phage lambda promoter pL, regulated by the bacteriophage lambda cI857 repressor.^52^ Plasmids were cloned into chemically competent POP2136 cells,^53^ grown at 30 °C, and purified using the Zymo Miniprep Kit.

DNA constructs for the expression of the proteins in CFPS were made using the pJL1 backbone plasmid as previously described^54^ and purified using the Zymo Midiprep Kit.

### S150 Lysate Preparation

S150 lysate was prepared as previously reported.^18^ One liter of 2X YTPG medium (containing 18 g/L of glucose) was inoculated with 10 mL of an overnight culture of MRE600. The 1 L culture was incubated at 37 °C while being shaken at 250 rpm until the OD600 reached 2.8. The culture was then immediately centrifuged at 5,000*g* for 10 min at 4 °C. Throughout the handling process, cells were kept on ice and as cold as possible. The supernatant was discarded, and the resulting pellet was suspended in S30 buffer (10 mM TrisOAc pH 8.2, 14 mM Mg(OAc)_2_, 60 mM KOAc). The cell resuspension was then subjected to two additional spins at 10,000*g* for 3 min each. Between each spin, the supernatant was removed, and the pellet was resuspended in 40 mL of fresh S30 buffer. Following the third spin, the pellets were weighed and immediately flash-frozen in liquid nitrogen before being stored at −80 °C.

After thawing on ice for 20 min, S30 buffer was added at a ratio of 5 mL per 1 g of cell mass, and then the cells were resuspended by vortexing until fully in solution. 100 μL of HALT Protease Inhibitor Cocktail was added per 10 mL of cell suspension, and 75 μL of Takara Recombinant RNase inhibitor was added per 4 g of dry cell mass. Cell lysis was achieved using a C3 Avestin Homogenizer at a pressure of approximately 25,000 psig. Following lysis, a second aliquot of the Takara Recombinant RNase inhibitor was added. The resulting mixture was centrifuged at 12,000*g* at 4 °C for 15 min to remove cell debris. The supernatant was then layered on top of an equivalent volume of sucrose cushion buffer (20 mM Tris–HCl (pH 7.2 at 4 °C), 100 mM NH_4_Cl, 10 mM MgCl_2_, 0.5 mM EDTA, 2 mM DTT, 37.7% sucrose) in Ti70 tubes.

The samples were then spun in an ultracentrifuge at 90,000*g* for 18 h. After this first spin, the supernatant was carefully transferred to fresh Ti70 tubes and spun for 3 additional hours at 150,000*g*. The pellets (ribosome pellets) remaining in the first tubes were used to purify the r-proteins for TP70. After the second spin, the top 2/3 of the supernatant was collected and transferred into MWCO = 3.5 K dialysis tubing (SnakeSkin) and dialyzed 2 × 1.5 h × 3 L of S150 Extract Buffer at 4 °C. For the third dialysis, 3 L of fresh S150 Extract Buffer was used to dialyze overnight (12–15 h). S150 extract was concentrated using Centripreps (3 kDa MWCO) until *A*_260_ = 25 and *A*_280_ = 15. Extract was aliquoted and flash frozen in liquid nitrogen. TP70 was prepared from the ribosome pellets as previously described.^20^

### SQ171 Transformations and Plasmid Selections

Electrocompetent *E. coli* SQ171fg cells, harboring RiboT-v2 rRNA on a pCSacB plasmid and kanamycin resistance (KanR),^25,55^ were prepared and stored in 50 μL aliquots. The SQ171fg strain is a modified *E. coli* strain with all seven rRNA operons deleted from its genome. The pCSacB/KanR plasmid contains the sequence for RiboT-v2,^23^ which functions as the sole rRNA operon in the cell. To remove the original pCSacB-RiboT-v2 plasmid and introduce pAM552 plasmids carrying the rRNA sequence of interest and an ampicillin resistance gene, selection was performed by plating on sucrose and carbenicillin (Cb). Successful selection was confirmed by checking the strain’s resistance to Kan.

50 ng of purified mutant pAM552 plasmid transformed into the SQ171fg electrocompetent cells. The cell/plasmid mixture was then incubated in 850 μL of SOC in a 1.5 mL microcentrifuge tube at 37 °C while being shaken at 250 rpm for 1 h. After the incubation, 270 μL of the cell recovery was transferred to 2 mL of Super Optimal broth with Catabolite repression (SOC) supplemented with 50 μg/Ll Cb (Cb_50_) and 0.25% sucrose in a 14 mL plastic culture tube. The tubes were incubated overnight at 37 °C for 16–18 h. After incubation, the tubes were centrifuged at room temperature for 5 min at 4000*g*. Two mL of clear supernatant was removed, and the remaining cell pellet was concentrated into the remaining 270 μL. The concentrated cell suspension was plated on lysogeny broth (LB) agar plates containing 5% sucrose and 100 μg/mL of Cb. The plates were incubated at 37 °C until colonies appeared. Eight colonies were selected from each plate and spotted onto two LB-agar plates, one containing Cb_100_ and the other containing Kan_50_. Colonies that grew successfully on Cb_100_ but not on Kan_50_ were chosen and cultured overnight in LB with Cb_100_ for midiprep by using the ZymoPURE II Plasmid Midiprep Kit. The midiprepped plasmids were then subjected to Sanger sequencing to confirm that the operon sequence was as expected.

Constructs that did not yield colonies on LB-Suc_5%_-Cb_100_ plates underwent two subsequent transformation and selection attempts to confirm their inability to independently enable cell growth in the SQ171fg strain. Constructs that produced colonies on both antibiotics were investigated further by picking and spot plating additional colonies. If troubleshooting failed, transformations were repeated up to three times before concluding that the construct was unsuccessful.

To confirm that the cells relied solely on the mutated ribosomes, overnight cultures of the successfully transformed SQ171fg strains were grown in 5 mL volumes, and total RNA was extracted using the QiagenTM RNeasy Mini kit. RT-PCRs were conducted using the Invitrogen SuperScript IV One-Step RT-PCR system to amplify regions of rRNA that contained mutations in the operons. The amplified products were then Sanger sequenced.

### Growth Curve Assays

Overnight cultures of SQ171fg solely expressing the desired rRNA operon were diluted and normalized to OD600 of 0.01, in antibiotic-free LB medium. Seven replicates of 180 μL each were plated on Corning 96-well flat bottom plates, with MG1655 (SQ171fg parent strain) and BL21 Star (DE3) (common protein production strain) serving as positive controls and noninoculated LB as blank. Plates were incubated in an Agilent BioTek Synergy Neo2Microplate Reader at 37 °C for 25 h while measuring cell density (OD600) every 20 min. Readings from the blank wells were averaged and subtracted from all of the data points before analysis. Wells containing OD600 values that fell more than two standard deviations away from the median were categorized as outliers and excluded from the analysis. Lag time for each well was output as a calculation from the Neo2 software.

### Ribosome Purifications and Testing

500 mL of LB-Miller was induced with an overnight culture of strain containing desired ribosomes, targeting an OD of 0.05. Cells were grown at 37 °C at 250 rpm until they reached an OD of 0.6–0.8. The cells were then pelleted via centrifugation for 10 min at 8,000*g* at 4 °C. Supernatant was removed, and the pellet was resuspended by vortexing in 25 mL of Buffer A (20 mM Tris-chloride pH 7.2 at 4 °C, 100 mM ammonium chloride, 10 mM magnesium chloride, 0.5 mM EDTA, 2 mM DTT). The pellet was washed in Buffer A for a total of three times, at which point the pellet was flash frozen and stored at −80 °C.

The cell pellet was resuspended in 1 mL of Buffer A per gram of cell pellet and lysed by sonication (50% Amplitude, 45 s ON, 59 s OFF, 950 J per mL of suspension). The sonicated cell suspension was then diluted to a total volume of 13 mL in Buffer A and centrifuged for 10 min at 12,000*g*. The supernatant (clarified lysate) was layered on top of 13 mL of Buffer B (20 mM Tris-HCl pH 7.2, 100 mM NH_4_Cl, 10 mM MgCl_2_, 0.5 mM EDTA, 2 mM DTT, 37.7% sucrose) in a Ti70 ultracentrifuge tube. Samples were spun at 90,000*g* for 18 h, at which point the resulting pellet was resuspended in Buffer C (10 mM Tris-OAc pH 7.5, 60 mM NH_4_Cl, 7.5 mM Mg(OAc)_2_, 0.5 mM EDTA, 2 mM DTT) and normalized to 25 μM. Ribosomes were added into blank iSAT reactions (no TP70 or pT7rrnB plasmid) to reach a final concentration of 4 μM.

### S12 Extract Preparation

Cell growth for extract preparation was carried out as previously described.^54,56,57^ Overnight cultures of strains used were used to inoculate 100 mL of LB-Miller at an OD of 0.05. The cells were grown at 37 °C and 250 rpm, and the OD was monitored until they reached an OD of 2.8. The culture was then spun down for 10 min at 12,000*g* at 4 °C. The pellet was resuspended in 25 mL of S30 Buffer by vortexing and spun for 2 min at 12,000*g*. This was repeated a total of three times, at which point the pellet was weighed and flash frozen in liquid nitrogen to be stored at −80 °C.

The pellet was thawed on ice and resuspended with 1 mL of S30 Buffer per gram of pellet in a 1.5 mL Eppendorf tube. The cells were then lysed via sonication (50% amplitude, 45 s ON, 59 s OFF, 950 J per mL of suspension) and centrifuged for 10 min at 12,000*g*. The supernatant was aliquoted and flash frozen for use as an S12 extract.

### AdhE2 Activity Quantification

CFPS reactions for the expression of pJL1-AdhE2 were set up with S12 lysates AAA and MG1655 as described in the CFPS Reactions section and run overnight at 30 °C. Negative control reactions were set up with pJL1-sfGFP, as the expression of sfGFP should not enable improved butyraldehyde conversion to butanol. Butyraldehyde conversion reactions then were assembled in 1.5 mL tubes to contain total AdhE2 concentrations of 0.075 μM by adding in corresponding volumes of overnight AAA and MG1655 CFPS AdhE2 expression reactions (as quantified by ^14^C-Leucine incorporation).

The overnight reactions were then mixed with 10 mM butyraldehyde, 10 mM Mg(Glu)_2_, 10 mM NH_4_(Glu), 134 mM KGlu, and 500 mM BisTris buffer. NADH (20 mM) was added to initiate the reaction, and samples were quenched after 1 h by adding an equivalent volume of 10% (w/v) trichloroacetic acid. Eppendorf tubes containing the quenched reactions were spun at a maximum speed for 10 min, at which point the supernatant was transferred to HPLC vials. Five μL of supernatant was injected into an Agilent 1290 HPLC with a Bio-Rad Fast Acid Analysis column held at 40 °C using 0.1% formic acid as the mobile phase flowing at 0.6 mL/min. Butanol concentrations were determined using refractive index values compared with a standard curve.

### Spot Growth Experiment

SQ171fg strains containing the single operon ribosomes were grown overnight in 3 mL cultures, with Cb_50_. The following morning, each culture was normalized to an OD_600_ of 1. Four 10-fold serial dilutions of each construct were prepared (OD_600_ = 0.1, 0.01, 0.001, 0.0001). 3-μL of each dilution was carefully pipetted onto a Cb_50_ plate. Plates were incubated at 37 °C and imaged as soon as a construct at the most dilute concentration showed growth detectable by eye. Spot growth experiments were completed three separate times to ensure consistent results.

### Radioactive Quantification of CFPS Yields

Total CFPS yields were quantified by incorporation of ^14^C-leucine (PerkinElmer) as previously described.^58−60^^14^C-Leucine was included in CFPS reactions to reach a final concentration of 10 μM in triplicate 15 μL reactions and incubated overnight at 30 °C with continuous shaking in a plate reader. Five μL of each reaction was mixed with an equivalent volume of 0.5 N KOH and incubated for 20 min at 37 °C. Five μL of each reaction mixture was then spotted onto two separate 96-well filtermats (PerkinElmer 1450-421) and dried under a heat lamp. One of the mats was washed three times in 5% trichloroacetic acid solution at 4 °C to precipitate protein products (with 15 min incubations) and a final wash in 100% ethanol before being fully dried under a heat lamp. Radioactivity was measured by a liquid scintillation counter (PerkinElmer MicroBeta) compared to the unwashed filtermat.

### AlphaLISA Assay

The AlphaLISA assay leverages the use of proprietary donor and acceptor beads that enable detection of protein–protein interactions based on bead proximity.^40^ AlphaLISA assays were run based on a previously published protocol^34^ in a 50 mM HEPES pH 7.4, 150 mM NaCl, 1 mg/mL BSA, and 0.015% v/v Triton X-100 buffer (“Alpha buffer”). Reaction components were dispensed into a ProxiPlate-384 Plus (PerkinElmer 6008280) destination plate from a 384-well polypropylene 2.0 Plus source microplate (Labcyte, PPL-0200) using an Echo 525 liquid acoustic liquid handler. The assays were run in a solution of 50 mM HEPES (pH 7.4), 150 mM NaCl, 1 mg/mL BSA, and 0.015% v/v Triton X-100 buffer (“Alpha buffer”). Anti-FLAG donor beads (PerkinElmer) were used to immobilize TRI2-2 protein, which was expressed with a sFLAG tag on its C-terminus.^61^ His-tagged stabilized trimeric S protein (S6P) (Acro SPN-C52H9), which has previously been shown to bind TRI2-2,^43^ was immobilized onto the acceptor bead. The final concentrations of the donor and acceptor beads were 0.08 and 0.02 mg/mL, respectively. S6P and CFPS reactions to produce TRI2-2 were cross titrated with final dilutions ranging from 25 to 0 nM for S6P and 20-fold to 6400-fold dilutions for TRI2-2 and incubated for 1 h at room temperature. The donor and acceptor beads were then added to the wells and incubated for an additional 1 h at room temperature. Chemiluminescence was measured on a Tecan Infinite M1000 Pro using the AlphaLISA filter with an excitation time of 100 ms, an integration time of 300 ms, and a settle time of 20 ms after 10 min of incubation inside the instrument as previously reported.^62^

### iSAT Reactions

iSAT reactions were assembled with four 5-μL replicates per rRNA construct being tested based on previous work.^16^ Reactions contained 8 mM magnesium glutamate, 10 mM ammonium glutamate, 130 mM potassium glutamate, 0.85 mM each of GTP, UTP, and CTP, 1.2 mM ATP, 34 μg/mL folinic acid, 0.171 mg/mL *E*. *coli* tRNA, 0.33 mM NAD, 0.27 mM CoA, 4 mM oxalic acid, 1 mM putrescine, 1.5 mM spermidine, 57 mM HEPES, 2 mM 20 amino acids, 37 mM PEP, ∼300 nM total protein of the 70S ribosome (TP70), 60 μg/mL T7 RNA polymerase, 0.50 μL of PEG-8000 40% (SigmaAldrich), and 1.83 μL of S150 extract (in a 5 μL reaction). The pJL1-sfGFP plasmid concentration was 6.27 ng/μL, and the pT7rrn plasmid concentration was 20.78 ng/μL.

The Echo 525 Acoustic Liquid Handler was used to assemble reaction components (separated into a master mix and individual rRNA plasmids to be tested) into 384-well nunc_267461 plates from a 384-well Polypropylene 2.0 Plus source microplate (Labcyte, PPL-0200). The nunc_267461 plate was then spun down, and reactions were run in a plate reader at 37 °C, measuring sfGFP fluorescence (excitation: 485 nm, emission: 528 nm) every 15 min and with constant shaking for 15 h.

### CFPS Reactions

CFPS reactions were set up as previously published^58−60,63^ work. 15-μL reactions were set up in triplicate on 384-well nunc_267461 plates. Reactions contained 8 mM magnesium glutamate, 10 mM ammonium glutamate, 130 mM potassium glutamate, 0.85 mM each of GTP, UTP, and CTP, 1.2 mM ATP, 34 μg/mL folinic acid, 0.171 mg/mL *E*. *coli* tRNA, 0.33 mM NAD, 0.27 mM CoA, 4 mM oxalic acid, 1 mM putrescine, 1.5 mM spermidine, 57 mM HEPES, 2 mM 20 amino acids, 0.03 M phosphoenolpyruvate, 36 μg/mL T7 RNA polymerase, 2.4 μL of S12 lysate, and 13.3 ng/μL of the pJL1 backbone plasmid. Reactions were incubated at 30 °C with continuous shaking for 15 h, and fluorescence (excitation: 485 nm, emission: 528 nm) was measured every 5 min for sfGFP expression.

## Supporting Information Available

Figures showing polymorphisms of operon sequences, iSAT activities of all constructs tested in this study, and AdhE2 activity assay control data. Tables containing sequences of all plasmids and primers used as well as growth data of all strains generated in this study (PDF)
Transparent Peer Review report available (PDF)
