# Supplementary material for: Ribosome Pool Engineering Increases Protein Biosynthesis Yields
Source: ACS Cent Sci. 2024 Mar 20;10(4):871–81. doi: 10.1021/acscentsci.3c01413 (PMC11046459; doi:10.1021/acscentsci.3c01413)
Supplement: Supplementary file 1 — oc3c01413_si_001.pdf [file oc3c01413_si_001.pdf]

Supplementary Information

Ribosome pool engineering increases protein biosynthesis yields

Camila Kofman[1], Jessica A. Willi[1], Ashty S. Karim[1], and Michael C. Jewett[1,2*]

Affiliations
[1] Department of Chemical and Biological Engineering, Northwestern University, Evanston, IL, USA
[2] Department of Bioengineering, Stanford University, Stanford CA, USA

*Correspondence should be addressed to mjewett@stanford.edu

Table of Contents

Supplementary Figures **S3**

Figure S1: Polymorphism mapping onto rRNA structure. 23S rRNA is shown in green, with polymorphisms highlighted as red spheres. 16S rRNA is shown in dark blue, with polymorphisms highlighted as orange spheres. 5S rRNA is shown in light blue, with polymorphisms highlighted as magenta spheres. S3

Figure S2. 70S sfGFP expression in iSAT reaction background for all single-operon constructs purified. Data presented are means of n = 3 experiments with standard deviation shown. S4

Figure S3. Amount of butanol produced by equivalent concentration of AdhE2 expressed in AAA and MG1655 lysate. Data presented are means of n = 3 experiments with standard deviation shown. S5

Supplementary Tables **S6**

Table S1. Plasmid sequence for pT7rrnBBB with backbone sequence bolded and highlighted in gray. S6

Table S2. 16S and 23S rRNA sequence variants for testing in iSAT. Constructs were ordered individually from a DNA synthesis company. Backbone sequence is maintained as that from pT7_BBB. S13

Table S3. Primers for cloning and sequence confirmation of rrn operon constructs. S36

Table S4. Plasmid sequences for protein expression panel. S37

Table S5. Growth data of all strains included in this study. Lag time and doubling times shown. Data represent mean and standard deviation (SD) of n=7 replicates. Replicates containing outliers that fell >2 standard deviations from the median were excluded from analysis. S56

Supplementary Figures

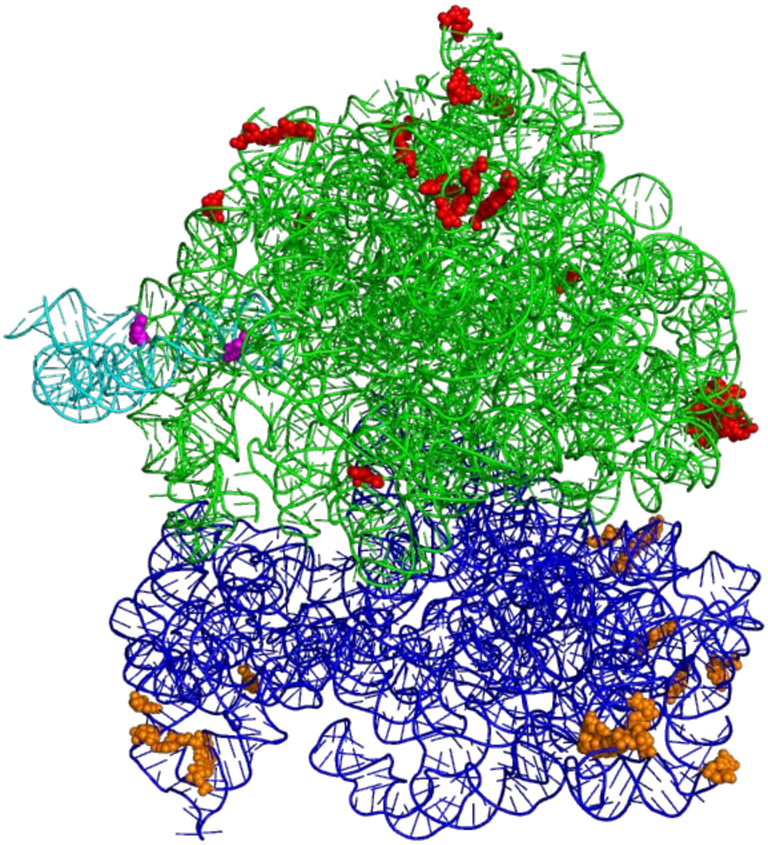

Figure S1: Polymorphism mapping onto rRNA structure. 23S rRNA is shown in green, with polymorphisms highlighted as red spheres. 16S rRNA is shown in dark blue, with polymorphisms highlighted as orange spheres. 5S rRNA is shown in light blue, with polymorphisms highlighted as magenta spheres.

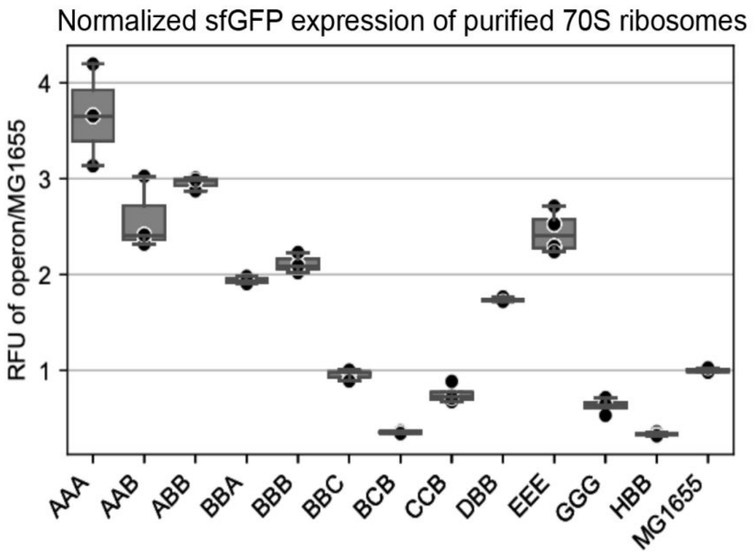

Figure S2. 70S sfGFP expression in iSAT reaction background for all single-operon constructs purified. Data presented are means of n = 3 experiments with standard deviation shown.

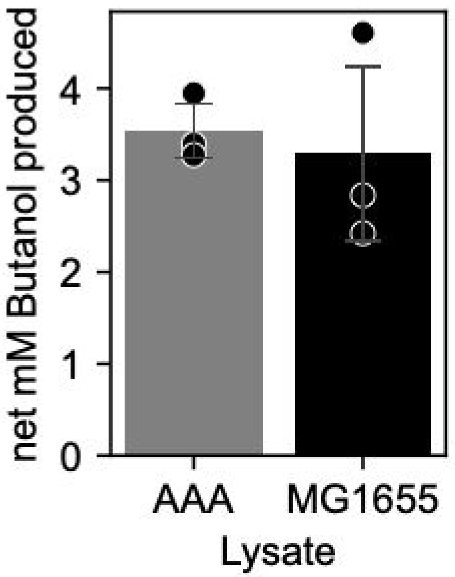

Figure S3. Amount of butanol produced by equivalent concentration of AdhE2 expressed in AAA and MG1655 lysate. Data presented are means of n = 3 experiments with standard deviation shown.

Supplementary Tables

Table S1. Plasmid sequence for pT7rrnBBB with backbone sequence bolded and highlighted in gray.

GGTTAAGCGACTAAGCGTACACGGTGGATGCCCTGGCAGTCAGAGGCGAT
GAAGGACGTGCTAATCTGCGATAAGCGTCGGTAAGGTGATATGAACCGTTA
TAACCGGCGATTTCCGAATGGGGAAACCCAGTGTGTTTCGACACACTATCA
TTAACTGAATCCATAGGTTAATGAGGCGAACCGGGGGAACTGAAACATCTA
AGTACCCCGAGGAAAAGAAATCAACCGAGATTCCCCCAGTAGCGGCGAGC
GAACGGGGAGCAGCCCAGAGCCTGAATCAGTGTGTGTGTTAGTGGAAGCG
TCTGGAAAGGCGCGCGATACAGGGTGACAGCCCCGTACACAAAAATGCAC
ATGCTGTGAGCTCGATGAGTAGGGCGGGACACGTGGTATCCTGTCTGAATA
TGGGGGGACCATCCTCCAAGGCTAAATACTCCTGACTGACCGATAGTGAAC
CAGTACCGTGAGGGAAAGGCGAAAAGAACCCCGGCGAGGGGAGTGAAAAA
GAACCTGAAACCGTGTACGTACAAGCAGTGGGAGCACGCTTAGGCGTGTG
ACTGCGTACCTTTTGTATAATGGGTCAGCGACTTATATTCTGTAGCAAGGTT
AACCGAATAGGGGAGCCGAAGGGAAACCGAGTCTTAACTGGGCGTTAAGT
TGCAGGGTATAGACCCGAAACCCGGTGATCTAGCCATGGGCAGGTTGAAG
GTTGGGTAACACTAACTGGAGGACCGAACCGACTAATGTTGAAAAATTAGC
GGATGACTTGTGGCTGGGGGTGAAAGGCCAATCAAACCGGGAGATAGCTG
GTTCTCCCCGAAAGCTATTTAGGTAGCGCCTCGTGAATTCATCTCCGGGGG
TAGAGCACTGTTTCGGCAAGGGGGTCATCCCGACTTACCAACCCGATGCAA
ACTGCGAATACCGGAGAATGTTATCACGGGAGACACACGGCGGGTGCTAA
CGTCCGTCGTGAAGAGGGAAACAACCCAGACCGCCAGCTAAGGTCCCAAA

GTCATGGTTAAGTGGGAAACGATGTGGGAAGGCCCAGACAGCCAGGATGT
TGGCTTAGAAGCAGCCATCATTTAAAGAAAGCGTAATAGCTCACTGGTCGA
GTCGGCCTGCGCGGAAGATGTAACGGGGCTAAACCATGCACCGAAGCTGC
GGCAGCGACGCTTATGCGTTGTTGGGTAGGGGAGCGTTCTGTAAGCCTGC
GAAGGTGTGCTGTGAGGCATGCTGGAGGTATCAGAAGTGCGAATGCTGAC
ATAAGTAACGATAAAGCGGGTGAAAAGCCCGCTCGCCGGAAGACCAAGGG
TTCCTGTCCAACGTTAATCGGGGCAGGGTGAGTCGACCCCTAAGGCGAGG
CCGAAAGGCGTAGTCGATGGGAAACAGGTTAATATTCCTGTACTTGGTGTT
ACTGCGAAGGGGGGACGGAGAAGGCTATGTTGGCCGGGCGACGGTTGTC
CCGGTTTAAGCGTGTAGGCTGGTTTTCCAGGCAAATCCGGAAAATCAAGGC
TGAGGCGTGATGACGAGGCACTACGGTGCTGAAGCAACAAATGCCCTGCT
TCCAGGAAAAGCCTCTAAGCATCAGGTAACATCAAATCGTACCCCAAACCG
ACACAGGTGGTCAGGTAGAGAATACCAAGGCGCTTGAGAGAACTCGGGTG
AAGGAACTAGGCAAAATGGTGCCGTAACTTCGGGAGAAGGCACGCTGATAT
GTAGGTGAGGTCCCTCGCGGATGGAGCTGAAATCAGTCGAAGATACCAGC
TGGCTGCAACTGTTTATTAAAAACACAGCACTGTGCAAACACGAAAGTGGA
CGTATACGGTGTGACGCCTGCCCGGTGCCGGAAGGTTAATTGATGGGGTT
AGCGCAAGCGAAGCTCTTGATCGAAGCCCCGGTAAACGGCGGCCGTAACT
ATAACGGTCCTAAGGTAGCGAAATTCCTTGTCGGGTAAGTTCCGACCTGCA
CGAATGGCGTAATGATGGCCAGGCTGTCTCCACCCGAGACTCAGTGAAATT
GAACTCGCTGTGAAGATGCAGTGTACCCGCGGCAAGACGGAAAGACCCCG
TGAACCTTTACTATAGCTTGACACTGAACATTGAGCCTTGATGTGTAGGATA
GGTGGGAGGCTTTGAAGTGTGGACGCCAGTCTGCATGGAGCCGACCTTGA

AATACCACCCTTTAATGTTTGATGTTCTAACGTTGACCCGTAATCCGGGTTG

CGGACAGTGTCTGGTGGGTAGTTTGACTGGGGCGGTCTCCTCCTAAAGAG

TAACGGAGGAGCACGAAGGTTGGCTAATCCTGGTCGGACATCAGGAGGTT

AGTGCAATGGCATAAGCCAGCTTGACTGCGAGCGTGACGGCGCGAGCAGG

TGCGAAAGCAGGTCATAGTGATCCGGTGGTTCTGAATGGAAGGGCCATCG

CTCAACGGATAAAAGGTACTCCGGGGATAACAGGCTGATACCGCCCAAGA

GTTCATATCGACGGCGGTGTTTGGCACCTCGATGTCGGCTCATCACATCCT

GGGGCTGAAGTAGGTCCCAAGGGTATGGCTGTTCGCCATTTAAAGTGGTAC

GCGAGCTGGGTTTAGAACGTCGTGAGACAGTTCGGTCCCTATCTGCCGTG

GGCGCTGGAGAACTGAGGGGGGCTGCTCCTAGTACGAGAGGACCGGAGT

GGACGCATCACTGGTGTTCGGGTTGTCATGCCAATGGCACTGCCCGGTAG

CTAAATGCGGAAGAGATAAGTGCTGAAAGCATCTAAGCACGAAACTTGCCC

CGAGATGAGTTCTCCCTGACCCTTTAAGGGTCCTGAAGGAACGTTGAAGAC

GACGACGTTGATAGGCCGGGTGTGTAAGCGCAGCGATGCGTTGAGCTAAC

CGGTACTAATGAACCGTGAGGCTTAACCTTACAACGCCGAAGCTGTTTTGG

CGGATGAGAGAAGATTTTCAGCCTGATACAGATTAAATCAGAACGCAGAAG

CGGTCTGATAAAACAGAATTTGCCTGGCGGCAGTAGCGCGGTGGTCCCAC

CTGACCCCATGCCGAACTCAGAAGTGAAACGCCGTAGCGCCGATGGTAGT

GTGGGGTCTCCCCATGCGAGAGTAGGGAACTGCCAGGCAT**CAAATAAAAC**

GAAAGGCTCAGTCGAAAGACTGGGCCTTTCGTTTTATCTGTTGTTTGTCGG

TGAACGCTCTCCTGAGTAGGACAAATCCGCCGGGAGCGGATTTGAACGTT

GCGAAGCAACGGCCCGGAGGGTGGCGGGCAGGACGCCCGCCATAAACT

GCCAGGCATCAAATTAAGCAGAAGGCCATCCTGACGGATGGCCTTTTTGC

GTTTCTACAAACTCTTCCTGTCGTCATATCTACAAGCCGGCGCCAAATT

GACAATTACTCATCCGGCTCGAATAATGTGTGGAACTTAAACACACACAG

GAGGAAAACATATGTCTATCCAGCACTTCCGTGTTGCGCTGATCCCGTTCT

TCGCGGCGTTCTGCCTGCCGGTTTTCGCGCACCCGGAAACCCTGGTTAAA

GTTAAAGACGCGGAAGACCAGCTGGGTGCGCGTGTTGGTTACATCGAAC

TGGACCTGAACTCTGGTAAAATCCTGGAATCTTTCCGTCCGGAAGAACGT

TTCCCGATGATGTCTACCTTCAAAGTTCTGCTGTGCGGTGCGGTTCTGTCT

CGTGTTGACGCGGGTCAGGAACAGCTGGGTCGTCGTATCCACTACTCTCA

GAACGACCTGGTTGAATACTCTCCCGTTACCGAAAAACACCTGACCGACG

GTATGACCGTTCGTGAACTGTGCTCTGCGGCGATCACCATGTCTGACAAC

ACCGCAGCGAACCTGCTGCTGACCACCATCGGTGGTCCGAAAGAACTGA

CCGCGTTCCTGCACAACATGGGCGACCACGTTACCCGTCTGGACCGTTGG

GAACCGGAACTGAACGAAGCGATCCCGAACGACGAACGTGACACCACCA

TGCCTGCGGCGATGGCGACCACCCTGCGTAAACTGCTGACCGGTGAACT

GCTGACCCTGGCATCTCGTCAGCAGCTGATCGACTGGATGGAAGCGGAC

AAAGTTGCGGGTCCGCTGCTGCGTTCTGCGCTGCCTGCGGGTTGGTTCAT

CGCGGACAAATCTGGTGCGGGTGAACGTGGTTCTCGTGGTATCATCGCG

GCGCTGGGTCCGGACGGTAAACCGTCTCGTATCGTTGTTATCTACACCAC

CGGTTCTCAGGCGACCATGGACGAACGTAACCGTCAGATCGCGGAAATC

GGTGCGTCTCTGATTAAACACTGGTAAACTCACTCCTAGCCCGCCTAATA

AGCGGGCTTTTTTTCTGCAGACCAAGTTTACTCATATATACTTTAGATTGAT

TTAAAACTTCATTTTTAATTTAAAAGGATCTAGGTGAAGATCCTTTTTGATA

ATCTCATGACCAAAATCCCTTAACGTGAGTTTTCGTTCCACTGAGCGTCAG

ACCCCGTAGAAAAGATCAAAGGATCTTCTTGAGATCCTTTTTTTCTGCGCG

TAATCTGCTGCTTGCAAACAAAAAAACCACCGCTACCAGCGGTGGTTTGT

TTGCCGGATCAAGAGCTACCAACTCTTTTTCCGAAGGTAACTGGCTTCAG

CAGAGCGCAGATACCAAATACTGTCCTTCTAGTGTAGCCGTAGTTAGGCC

ACCACTTCAAGAACTCTGTAGCACCGCCTACATACCTCGCTCTGCTAATC

CTGTTACCAGTGGCTGCTGCCAGTGGCGATAAGTCGTGTCTTACCGGGTT

GGACTCAAGACGATAGTTACCGGATAAGGCGCAGCGGTCGGGCTGAACG

GGGGGTTCGTGCACACAGCCCAGCTTGGAGCGAACGACCTACACCGAAC

TGAGATACCTACAGCGTGAGCTATGAGAAAGCGCCACGCTTCCCGAAGG

GAGAAAGGCGGACAGGTATCCGGTAAGCGGCAGGGTCGGAACAGGAGA

GCGCACGAGGGAGCTTCCAGGGGGAAACGCCTGGTATCTTTATAGTCCT

GTCGGGTTTCGCCACCTCTGACTTGAGCGTCGATTTTTGTGATGCTCGTCA

GGGGGGCGGAGCCTATGGAAAAACGCCAGCAACGCGGCCTTTTTACGGT

TCCTGGCCTTTTGCTGGTTAATACGACTCACTATAGGGGCCGCTGAGAAA

AAGCGAAGCGGCACTGCTCTTTAACAATTTATCAGACAATCTGTGTGGGC

ACTCGAAGATACGGATTCTTAACGTCGCAAGACGAAAAATGAATACCAAG

TCTCAAGAGTGAACACGTAATTCATTACGAAGTTTAATTCTTTGAGCGTCA

AACTTTTAAATTGAAGAGTTTGATCATGGCTCAGATTGAACGCTGGCGGCA

GGCCTAACACATGCAAGTCGAACGGTAACAGGAAGAAGCTTGCTTCTTTGC

TGACGAGTGGCGGACGGGTGAGTAATGTCTGGGAAACTGCCTGATGGAGG

GGGATAACTACTGGAAACGGTAGCTAATACCGCATAACGTCGCAAGACCAA

AGAGGGGGACCTTCGGGCCTCTTGCCATCGGATGTGCCCAGATGGGATTA

GCTAGTAGGTGGGGTAACGGCTCACCTAGGCGACGATCCCTAGCTGGTCT

GAGAGGATGACCAGCCACACTGGAACTGAGACACGGTCCAGACTCCTACG
GGAGGCAGCAGTGGGGAATATTGCACAATGGGCGCAAGCCTGATGCAGCC
ATGCCGCGTGTATGAAGAAGGCCTTCGGGTTGTAAAGTACTTTCAGCGGGG
AGGAAGGGAGTAAAGTTAATACCTTTGCTCATTGACGTTACCCGCAGAAGA
AGCACCGGCTAACTCCGTGCCAGCAGCCGCGGTAATACGGAGGGTGCAAG
CGTTAATCGGAATTACTGGGCGTAAAGCGCACGCAGGCGGTTTGTTAAGTC
AGATGTGAAATCCCCGGGCTCAACCTGGGAACTGCATCTGATACTGGCAAG
CTTGAGTCTCGTAGAGGGGGGTAGAATTCCAGGTGTAGCGGTGAAATGCG
TAGAGATCTGGAGGAATACCGGTGGCGAAGGCGGCCCCCTGGACGAAGAC
TGACGCTCAGGTGCGAAAGCGTGGGGAGCAAACAGGATTAGATACCCTGG
TAGTCCACGCCGTAAACGATGTCGACTTGGAGGTTGTGCCCTTGAGGCGT
GGCTTCCGGAGCTAACGCGTTAAGTCGACCGCCTGGGGAGTACGGCCGCA
AGGTTAAAACTCAAATGAATTGACGGGGGCCCGCACAAGCGGTGGAGCAT
GTGGTTTAATTCGATGCAACGCGAAGAACCTTACCTGGTCTTGACATCCAC
GGAAGTTTTCAGAGATGAGAATGTGCCTTCGGGAACCGTGAGACAGGTGCT
GCATGGCTGTCGTCAGCTCGTGTTGTGAAATGTTGGGTTAAGTCCCGCAAC
GAGCGCAACCCTTATCCTTTGTTGCCAGCGGTCCGGCCGGGAACTCAAAG
GAGACTGCCAGTGATAAACTGGAGGAAGGTGGGGATGACGTCAAGTCATC
ATGGCCCTTACGACCAGGGCTACACACGTGCTACAATGGCGCATACAAAGA
GAAGCGACCTCGCGAGAGCAAGCGGACCTCATAAAGTGCGTCGTAGTCCG
GATTGGAGTCTGCAACTCGACTCCATGAAGTCGGAATCGCTAGTAATCGTG
GATCAGAATGCCACGGTGAATACGTTCCCGGGCCTTGTACACACCGCCCG
TCACACCATGGGAGTGGGTTGCAAAAGAAGTAGGTAGCTTAACCTTCGGGA

GGGCGCTTACCACTTTGTGATTCATGACTGGGGTGAAGTCGTAACAAGGTA
ACCGTAGGGGAACCTGCGGTTGGATCACCTCCTTACCTTAAAGAAGCGTAC
TTTGTAGTGCTCACACAGATTGTCTGATAGAAAGTGAAAAGCAAGGCGTTTA
CGCGTTGGGAGTGAGGCTGAAGAGAATAAGGCCGTTCGCTTTCTATTAATG
AAAGCTCACCCTACACGAAAATATCACGCAACGCGTGATAAGCAATTTTCGT
GTCCCCTTCGTCTAGAGGCCCAGGACACCGCCCTTTCACGGCGGTAACAG
GGGTTCGAATCCCCTAGGGGACGCCACTTGCTGGTTTGTGAGTGAAAGTC
GCCGACCTTAATATCTCAAAACTCATCTTCGGGTGATGTTTGAGATATTTGC
TCTTTAAAAATCTGGATCAAGCTGAAAATTGAAACACTGAACAACGAGAGTT
GTTCGTGAGTCTCTCAAATTTTCGCAACACGATGATGAATCGAAAGAAACAT
CTTCGGGTTGTGA

Table S2. 16S and 23S rRNA sequence variants for testing in iSAT. Constructs were ordered individually from a DNA synthesis company. Backbone sequence is maintained as that from pT7_BBB.

pT7_rrnAB B:	AAATTGAAGAGTTTGATCATGGCTCAGATTGAACGCTGGCGGCAG GCCTAACACATGCAAGTCGAACGGTAACAGGAAGAAGCTTGCTTC TTTGCTGACGAGTGGCGGACGGGTGAGTAATGTCTGGGAAACTGC CTGATGGAGGGGGATAACTACTGGAAACGGTAGCTAATACCGCAT AACGTCGCAAGACCAAAGAGGGGTACCTTCGGGCCTCTTGCCATC GGATGTGCCCAGATGGGATTAGCTAGTAGGTGGGGTAACGGCTCA CCTAGGCGACGATCCCTAGCTGGTCTGAGAGGATGACCAGCCACA CTGGAACTGAGACACGGTCCAGACTCCTACGGGAGGCAGCAGTG GGGAATATTGCACAATGGGCGCAAGCCTGATGCAGCCATGCCGCG TGTATGAAGAAGGCCTTCGGGTTGTAAAGTACTTTCAGCGGGGAG GAAGGGAGTAAAGTTAATACCTTTGCTCATTGACGTTACCCGCAGA AGAAGCACCGGCTAACTCCGTGCCAGCAGCCGCGGTAATACGGA GGGTGCAAGCGTTAATCGGAATTACTGGGCGTAAAGCGCACGCAG GCGGTTTGTTAAGTCAGATGTGAAATCCCCGGGCTCAACCTGGGA ACTGCATCTGATACTGGCAAGCTTGAGTCTCGTAGAGGGGGGTAG AATTCCAGGTGTAGCGGTGAAATGCGTAGAGATCTGGAGGAATAC CGGTGGCGAAGGCGGCCCCCTGGACGAAGACTGACGCTCAGGTG CGAAAGCGTGGGGAGCAAACAGGATTAGATACCCTGGTAGTCCAC GCCGTAAACGATGTCGACTTGGAGGTTGTGCCCTTGAGGCGTGGC TTCCGGAGCTAACGCGTTAAGTCGACCGCCTGGGGAGTACGGCC GCAAGGTTAAAACTCAAATGAATTGACGGGGGCCCGCACAAGCGG

	TGGAGCATGTGGTTTAATTCGATGCAACGCGAAGAACCTTACCTGG TCTTGACATCCACGGAAGTTTTCAGAGATGAGAATGTGCCTTCGGG AACCGTGAGACAGGTGCTGCATGGCTGTCGTCAGCTCGTGTTGTG AAATGTTGGGTTAAGTCCCGCAACGAGCGCAACCCTTATCCTTTGT TGCCAGCGGTCCGGCCGGGAACTCAAAGGAGACTGCCAGTGATA AACTGGAGGAAGGTGGGGATGACGTCAAGTCATCATGGCCCTTAC GACCAGGGCTACACACGTGCTACAATGGCGCATACAAAGAGAAGC GACCTCGCGAGAGCAAGCGGACCTCATAAAGTGCGTCGTAGTCCG GATTGGAGTCTGCAACTCGACTCCATGAAGTCGGAATCGCTAGTAA TCGTGGATCAGAATGCCACGGTGAATACGTTCCCGGGCCTTGTAC ACACCGCCCGTCACACCATGGGAGTGGGTTGCAAAAGAAGTAGGT AGCTTAACCTTCGGGAGGGCGCTTACCACTTTGTGATTCATGACTG GGGTGAAGTCGTAACAAGGTAACCGTAGGGGAACCTGCGGTTGGA TCACCTCCTTA
pT7_rrnCBB:	AAATTGAAGAGTTTGATCATGGCTCAGATTGAACGCTGGCGGCAG GCCTAACACATGCAAGTCGAACGGTAACAGGAAACAGCTTGCTGT TTCGCTGACGAGTGGCGGACGGGTGAGTAATGTCTGGGAAACTGC CTGATGGAGGGGGATAACTACTGGAAACGGTAGCTAATACCGCAT AACGTCGCAAGACCAAAGAGGGGGACCTTCGGGCCTCTTGCCATC AGATGTGCCCAGATGGGATTAGCTAGTAGGTGGGGTAACGGCTCA CCTAGGCGACGATCCCTAGCTGGTCTGAGAGGATGACCAGCCACA CTGGAACTGAGACACGGTCCAGACTCCTACGGGAGGCAGCAGTG

	GGGAATATTGCACAATGGGCGCAAGCCTGATGCAGCCATGCCGCG<br>TGTATGAAGAAGGCCTTCGGGTTGTAAAGTACTTTCAGCGGGGAG<br>GAAGGGAGTAAAGTTAATACCTTTGCTCATTGACGTTACCCGCAGA<br>AGAAGCACCGGCTAACTCCGTGCCAGCAGCCGCGGTAATACGGA<br>GGGTGCAAGCGTTAATCGGAATTACTGGGCGTAAAGCGCACGCAG<br>GCGGTTTGTTAAGTCAGATGTGAAATCCCCGGGCTCAACCTGGGA<br>ACTGCATCTGATACTGGCAAGCTTGAGTCTCGTAGAGGGGGGTAG<br>AATTCCAGGTGTAGCGGTGAAATGCGTAGAGATCTGGAGGAATAC<br>CGGTGGCGAAGGCGGCCCCCTGGACGAAGACTGACGCTCAGGTG<br>CGAAAGCGTGGGGAGCAAACAGGATTAGATACCCTGGTAGTCCAC<br>GCCGTAAACGATGTCGACTTGGAGGTTGTGCCCTTGAGGCGTGGC<br>TTCCGGAGCTAACGCGTTAAGTCGACCGCCTGGGGAGTACGGCC<br>GCAAGGTTAAAACTCAAATGAATTGACGGGGGCCCGCACAAGCGG<br>TGGAGCATGTGGTTTAATTCGATGCAACGCGAAGAACCTTACCTGG<br>TCTTGACATCCACGGAAGTTTTCAGAGATGAGAATGTGCCTTCGGG<br>AACCGTGAGACAGGTGCTGCATGGCTGTCGTCAGCTCGTGTTGTG<br>AAATGTTGGGTTAAGTCCCGCAACGAGCGCAACCCTTATCCTTTGT<br>TGCCAGCGGTCCGGCCGGGAACTCAAAGGAGACTGCCAGTGATA<br>AACTGGAGGAAGGTGGGGATGACGTCAAGTCATCATGGCCCTTAC<br>GACCAGGGCTACACACGTGCTACAATGGCGCATACAAAGAGAAGC<br>GACCTCGCGAGAGCAAGCGGACCTCATAAAGTGCGTCGTAGTCCG<br>GATTGGAGTCTGCAACTCGACTCCATGAAGTCGGAATCGCTAGTAA<br>TCGTGGATCAGAATGCCACGGTGAATACGTTCCCGGGCCTTGTAC

	ACACCGCCCGTCACACCATGGGAGTGGGTTGCAAAAGAAGTAGGTAGCTTAACCTTCGGGAGGGCGCTTACCACTTTGTGATTCATGACTGGGGTGAAGTCGTAACAAGGTAACCGTAGGGGAACCTGCGGTTGGATCACCTCCTTA
pT7_rrnDBB:	AAATTGAAGAGTTTGATCATGGCTCAGATTGAACGCTGGCGGCAGGCCTAACACATGCAAGTCGAACGGTAACAGGAAACAGCTTGCTGTTTCGCTGACGAGTGGCGGACGGGTGAGTAATGTCTGGGAAACTGCCTGATGGAGGGGGATAACTACTGGAAACGGTAGCTAATACCGCATAACGTCGCAAGACCAAAGAGGGGGACCCTCGGGCCTCTTGCCATCGGATGTGCCCAGATGGGATTAGCTTGTTGGTGGGGTAACGGCTCACCAAGGCGACGATCCCTAGCTGGTCTGAGAGGATGACCAGCCACACTGGAACTGAGACACGGTCCAGACTCCTACGGGAGGCAGCAGTGGGGAATATTGCACAATGGGCGCAAGCCTGATGCAGCCATGCCGCGTGTATGAAGAAGGCCTTCGGGTTGTAAAGTACTTTCAGCGGGGAGGAAGGGAGTAAAGTTAATACCTTTGCTCATTGACGTTACCCGCAGAAGAAGCACCGGCTAACTCCGTGCCAGCAGCCGCGGTAATACGGAGGGTGCAAGCGTTAATCGGAATTACTGGGCGTAAAGCGCACGCAGGCGGTTTGTTAAGTCAGATGTGAAATCCCCGGGCTCAACCTGGGAACTGCATCTGATACTGGCAAGCTTGAGTCTCGTAGAGGGGGGTAGAATTCCAGGTGTAGCGGTGAAATGCGTAGAGATCTGGAGGAATACCGGTGGCGAAGGCGGCCCCCTGGACGAAGACTGACGCTCAGGTGCGAAAGCGTGGGGAGCAAACAGGATTAGATACCCTGGTAGTCCAC

	GCCGTAAACGATGTCGACTTGGAGGTTGTGCCCTTGAGGCGTGGC TTCCGGAGCTAACGCGTTAAGTCGACCGCCTGGGGAGTACGGCC GCAAGGTTAAAACTCAAATGAATTGACGGGGGCCCGCACAAGCGG TGGAGCATGTGGTTTAATTCGATGCAACGCGAAGAACCTTACCTGG TCTTGACATCCACGGAAGTTTTCAGAGATGAGAATGTGCCTTCGGG AACCGTGAGACAGGTGCTGCATGGCTGTCGTCAGCTCGTGTTGTG AAATGTTGGGTTAAGTCCCGCAACGAGCGCAACCCTTATCCTTTGT TGCCAGCGGTCCGGCCGGGAACTCAAAGGAGACTGCCAGTGATA AACTGGAGGAAGGTGGGGATGACGTCAAGTCATCATGGCCCTTAC GACCAGGGCTACACACGTGCTACAATGGCGCATACAAAGAGAAGC GACCTCGCGAGAGCAAGCGGACCTCATAAAGTGCGTCGTAGTCCG GATTGGAGTCTGCAACTCGACTCCATGAAGTCGGAATCGCTAGTAA TCGTGGATCAGAATGCCACGGTGAATACGTTCCCGGGCCTTGTAC ACACCGCCCGTCACACCATGGGAGTGGGTTGCAAAAGAAGTAGGT AGCTTAACCTTCGGGAGGGCGCTTACCACTTTGTGATTCATGACTG GGGTGAAGTCGTAACAAGGTAACCGTAGGGGAACCTGCGGTTGGA TCACCTCCTTA
pT7_rrnGBB:	AAATTGAAGAGTTTGATCATGGCTCAGATTGAACGCTGGCGGCAG GCCTAACACATGCAAGTCGAACGGTAACAGGAAGCAGCTTGCTGC TTCGCTGACGAGTGGCGGACGGGTGAGTAATGTCTGGGAAGCTG CCTGATGGAGGGGGATAACTACTGGAAACGGTAGCTAATACCGCA

	TAATGTCGCAAGACCAAAGAGGGGGACCTTCGGGCCTCTTGCCAT CGGATGTGCCCAGATGGGATTAGCTTGTTGGTGGGGTAACGGCTC ACCAAGGCGACGATCCCTAGCTGGTCTGAGAGGATGACCAGCCAC ACTGGAACTGAGACACGGTCCAGACTCCTACGGGAGGCAGCAGT GGGGAATATTGCACAATGGGCGCAAGCCTGATGCAGCCATGCCGC GTGTATGAAGAAGGCCTTCGGGTTGTAAAGTACTTTCAGCGGGGA GGAAGGGAGTAAAGTTAATACCTTTGCTCATTGACGTTACCCGCAG AAGAAGCACCGGCTAACTCCGTGCCAGCAGCCGCGGTAATACGGA GGGTGCAAGCGTTAATCGGAATTACTGGGCGTAAAGCGCACGCAG GCGGTTTGTTAAGTCAGATGTGAAATCCCCGGGCTCAACCTGGGA ACTGCATCTGATACTGGCAAGCTTGAGTCTCGTAGAGGGGGGTAG AATTCCAGGTGTAGCGGTGAAATGCGTAGAGATCTGGAGGAATAC CGGTGGCGAAGGCGGCCCCCTGGACGAAGACTGACGCTCAGGTG CGAAAGCGTGGGGAGCAAACAGGATTAGATACCCTGGTAGTCCAC GCCGTAAACGATGTCGACTTGGAGGTTGTGCCCTTGAGGCGTGGC TTCCGGAGCTAACGCGTTAAGTCGACCGCCTGGGGAGTACGGCC GCAAGGTTAAAACTCAAATGAATTGACGGGGGCCCGCACAAGCGG TGGAGCATGTGGTTTAATTCGATGCAACGCGAAGAACCTTACCTGG TCTTGACATCCACGGAAGTTTTCAGAGATGAGAATGTGCCTTCGGG AACCGTGAGACAGGTGCTGCATGGCTGTCGTCAGCTCGTGTTGTG AAATGTTGGGTTAAGTCCCGCAACGAGCGCAACCCTTATCCTTTGT TGCCAGCGGTCCGGCCGGGAACTCAAAGGAGACTGCCAGTGATA AACTGGAGGAAGGTGGGGATGACGTCAAGTCATCATGGCCCTTAC

	GACCAGGGCTACACACGTGCTACAATGGCGCATACAAAGAGAAGC GACCTCGCGAGAGCAAGCGGACCTCATAAAGTGCGTCGTAGTCCG GATTGGAGTCTGCAACTCGACTCCATGAAGTCGGAATCGCTAGTAA TCGTGGATCAGAATGCCACGGTGAATACGTTCCCGGGCCTTGTAC ACACCGCCCGTCACACCATGGGAGTGGGTTGCAAAAGAAGTAGGT AGCTTAACCTTCGGGAGGGCGCTTACCACTTTGTGATTCATGACTG GGGTGAAGTCGTAACAAGGTAACCGTAGGGGAACCTGCGGTTGGA TCACCTCCTTA
pT7_rrnHB B:	AAATTGAAGAGTTTGATCATGGCTCAGATTGAACGCTGGCGGCAG GCCTAACACATGCAAGTCGAACGGTAACAGGAAGAAGCTTGCTTC TTTGCTGACGAGTGGCGGACGGGTGAGTAATGTCTGGGAAACTGC CTGATGGAGGGGGATAACTACTGGAAACGGTAGCTAATACCGCAT AACGTCGCAAGACCAAAGAGGGGGACCTTCGGGCCTCTTGCCATC GGATGTGCCCAGATGGGATTAGCTAGTAGGTGGGGTAACGGCTCA CCTAGGCGACGATCCCTAGCTGGTCTGAGAGGATGACCAGCCACA CTGGAACTGAGACACGGTCCAGACTCCTACGGGAGGCAGCAGTG GGGAATATTGCACAATGGGCGCAAGCCTGATGCAGCCATGCCGCG TGTATGAAGAAGGCCTTCGGGTTGTAAAGTACTTTCAGCGGGGAG GAAGGGAGTAAAGTTAATACCTTTGCTCATTGACGTTACCCGCAGA AGAAGCACCGGCTAACTCCGTGCCAGCAGCCGCGGTAATACGGA GGGTGCAAGCGTTAATCGGAATTACTGGGCGTAAAGCGCACGCAG

	GCGGTTTGTTAAGTCAGATGTGAAATCCCCGGGCTCAACCTGGGA<br>ACTGCATCTGATACTGGCAAGCTTGAGTCTCGTAGAGGGGGGTAG<br>AATTCCAGGTGTAGCGGTGAAATGCGTAGAGATCTGGAGGAATAC<br>CGGTGGCGAAGGCGGCCCCCTGGACGAAGACTGACGCTCAGGTG<br>CGAAAGCGTGGGGAGCAAACAGGATTAGATACCCTGGTAGTCCAC<br>GCCGTAAACGATGTCGACTTGGAGGTTGTGCCCTTGAGGCGTGGC<br>TTCCGGAGCTAACGCGTTAAGTCGACCGCCTGGGGAGTACGGCC<br>GCAAGGTTAAAACTCAAATGAATTGACGGGGGCCCGCACAAGCGG<br>TGGAGCATGTGGTTTAATTCGATGCAACGCGAAGAACCTTACCTGG<br>TCTTGACATCCACAGAACTTTCCAGAGATGGATTGGTGCCTTCGGG<br>AACTGTGAGACAGGTGCTGCATGGCTGTCGTCAGCTCGTGTTGTG<br>AAATGTTGGGTTAAGTCCCGCAACGAGCGCAACCCTTATCTTTTGT<br>TGCCAGCGGTCCGGCCGGGAACTCAAAGGAGACTGCCAGTGATA<br>AACTGGAGGAAGGTGGGGATGACGTCAAGTCATCATGGCCCTTAC<br>GACCAGGGCTACACACGTGCTACAATGGCGCATACAAAGAGAAGC<br>GACCTCGCGAGAGCAAGCGGACCTCATAAAGTGCGTCGTAGTCCG<br>GATTGGAGTCTGCAACTCGACTCCATGAAGTCGGAATCGCTAGTAA<br>TCGTGGATCAGAATGCCACGGTGAATACGTTCCCGGGCCTTGTAC<br>ACACCGCCCGTCACACCATGGGAGTGGGTTGCAAAAGAAGTAGGT<br>AGCTTAACCTTCGGGAGGGCGCTTACCACTTTGTGATTCATGACTG<br>GGGTGAAGTCGTAACAAGGTAACCGTAGGGGAACCTGCGGTTGGA<br>TCACCTCCTTA

pT7_rrnBA<br>B:	GGTTAAGCGACTAAGCGTACACGGTGGATGCCCTGGCAGTCAGAG<br>GCGATGAAGGACGTGCTAATCTGCGATAAGCGTCGGTAAGGTGAT<br>ATGAACCGTTATAACCGGCGATTTCCGAATGGGGAAACCCAGTGT<br>GTTTCGACACACTATCATTAACTGAATCCATAGGTTAATGAGGCGA<br>ACCGGGGGAACTGAAACATCTAAGTACCCCGAGGAAAAGAAATCA<br>ACCGAGATTCCCCCAGTAGCGGCGAGCGAACGGGGAGCAGCCCA<br>GAGCCTGAATCAGTGTGTGTGTTAGTGGAAGCGTCTGGAAAGGCG<br>TGCGATACAGGGTGACAGCCCCGTACACAAAAATGCACATGCTGT<br>GAGCTCGATGAGTAGGGCGGGACACGTGGTATCCTGTCTGAATAT<br>GGGGGGACCATCCTCCAAGGCTAAATACTCCTGACTGACCGATAG<br>TGAACCAGTACCGTGAGGGAAAGGCGAAAAGAACCCCGGCGAGG<br>GGAGTGAAAAAGAACCTGAAACCGTGTACGTACAAGCAGTGGGAG<br>CACGCTTAGGCGTGTGACTGCGTACCTTTTGTATAATGGGTCAGC<br>GACTTATATTCTGTAGCAAGGTTAACCGAATAGGGGAGCCGAAGG<br>GAAACCGAGTCTTAACTGGGCGTTAAGTTGCAGGGTATAGACCCG<br>AAACCCGGTGATCTAGCCATGGGCAGGTTGAAGGTTGGGTAACAC<br>TAACTGGAGGACCGAACCGACTAATGTTGAAAAATTAGCGGATGAC<br>TTGTGGCTGGGGGTGAAAGGCCAATCAAACCGGGAGATAGCTGGT<br>TCTCCCCGAAAGCTATTTAGGTAGCGCCTCGTGAATTCATCTCCGG<br>GGGTAGAGCACTGTTTCGGCAAGGGGGTCATCCCGACTTACCAAC<br>CCGATGCAAACTGCGAATACCGGAGAATGTTATCACGGGAGACAC<br>ACGGCGGGTGCTAACGTCCGTCGTGAAGAGGGAAACAACCCAGA<br>CCGCCAGCTAAGGTCCCAAAGTCATGGTTAAGTGGGAAACGATGT

	GGGAAGGCCCAGACAGCCAGGATGTTGGCTTAGAAGCAGCCATCA TTTAAAGAAAGCGTAATAGCTCACTGGTCGAGTCGGCCTGCGCGG AAGATGTAACGGGGCTAAACCATGCACCGAAGCTGCGGCAGCGAC ACTATGTGTTGTTGGGTAGGGGAGCGTTCTGTAAGCCTGTGAAGG TGTGCTGTGAGGCATGCTGGAGGTATCAGAAGTGCGAATGCTGAC ATAAGTAACGATAAAGCGGGTGAAAAGCCCGCTCGCCGGAAGACC AAGGGTTCCTGTCCAACGTTAATCGGGGCAGGGTGAGTCGACCCC TAAGGCGAGGCCGAAAGGCGTAGTCGATGGGAAACAGGTTAATAT TCCTGTACTTGGTGTTACTGCGAAGGGGGGACGGAGAAGGCTATG TTGGCCGGGCGACGGTTGTCCCGGTTTAAGCGTGTAGGCTGGTTT TCCAGGCAAATCCGGAAAATCAAGGCTGAGGCGTGATGACGAGGC ACTACGGTGCTGAAGCAACAAATGCCCTGCTTCCAGGAAAAGCCT CTAAGCATCAGGTAACATCAAATCGTACCCCAAACCGACACAGGTG GTCAGGTAGAGAATACCAAGGCGCTTGAGAGAACTCGGGTGAAGG AACTAGGCAAAATGGTGCCGTAACTTCGGGAGAAGGCACGCTGAT ATGTAGGTGAAGCGACTTGCTCGTGGAGCTGAAATCAGTCGAAGA TACCAGCTGGCTGCAACTGTTTATTAAAAACACAGCACTGTGCAAA CACGAAAGTGGACGTATACGGTGTGACGCCTGCCCGGTGCCGGA AGGTTAATTGATGGGGTTAGCCGCAAGGCGAAGCTCTTGATCGAA GCCCCGGTAAACGGCGGCCGTAACTATAACGGTCCTAAGGTAGCG AAATTCCTTGTCGGGTAAGTTCCGACCTGCACGAATGGCGTAATGA TGGCCAGGCTGTCTCCACCCGAGACTCAGTGAAATTGAACTCGCT GTGAAGATGCAGTGTACCCGCGGCAAGACGGAAAGACCCCGTGA

	ACCTTTACTATAGCTTGACACTGAACATTGAGCCTTGATGTGTAGG ATAGGTGGGAGGCTTTGAAGTGTGGACGCCAGTCTGCATGGAGCC GACCTTGAAATACCACCCTTTAATGTTTGATGTTCTAACGTTGACCC GTAATCCGGGTTGCGGACAGTGTCTGGTGGGTAGTTTGACTGGGG CGGTCTCCTCCTAAAGAGTAACGGAGGAGCACGAAGGTTGGCTAA TCCTGGTCGGACATCAGGAGGTTAGTGCAATGGCATAAGCCAGCT TGACTGCGAGCGTGACGGCGCGAGCAGGTGCGAAAGCAGGTCAT AGTGATCCGGTGGTTCTGAATGGAAGGGCCATCGCTCAACGGATA AAAGGTACTCCGGGGATAACAGGCTGATACCGCCCAAGAGTTCAT ATCGACGGCGGTGTTTGGCACCTCGATGTCGGCTCATCACATCCT GGGGCTGAAGTAGGTCCCAAGGGTATGGCTGTTCGCCATTTAAAG TGGTACGCGAGCTGGGTTTAGAACGTCGTGAGACAGTTCGGTCCC TATCTGCCGTGGGCGCTGGAGAACTGAGGGGGGCTGCTCCTAGT ACGAGAGGACCGGAGTGGACGCATCACTGGTGTTCGGGTTGTCAT GCCAATGGCACTGCCCGGTAGCTAAATGCGGAAGAGATAAGTGCT GAAAGCATCTAAGCACGAAACTTGCCCCGAGATGAGTTCTCCCTG ACTCCTTGAGAGTCCTGAAGGAACGTTGAAGACGACGACGTTGAT AGGCCGGGTGTGTAAGCGCAGCGATGCGTTGAGCTAACCGGTACT AATGAACCGTGAGGCTTAACCTT
pT7_rrnBC B:	GGTTAAGCGACTAAGCGTACACGGTGGATGCCCTGGCAGTCAGAG GCGATGAAGGACGTGCTAATCTGCGATAAGCGTCGGTAAGGTGAT

	ATGAACCGTTATAACCGGCGATTTCCGAATGGGGAAACCCAGTGT<br>GTTTCGACACACTATCATTAACTGAATCCATAGGTTAATGAGGCGA<br>ACCGGGGGAACTGAAACATCTAAGTACCCCGAGGAAAAGAAATCA<br>ACCGAGATTCCCCCAGTAGCGGCGAGCGAACGGGGAGCAGCCCA<br>GAGCCTGAATCAGTATGTGTGTTAGTGGAAGCGTCTGGAAAGGCG<br>CGCGATACAGGGTGACAGCCCCGTACACAAAAATGCACATATTGT<br>GAGCTCGATGAGTAGGGCGGGACACGTGGTATCCTGTCTGAATAT<br>GGGGGGACCATCCTCCAAGGCTAAATACTCCTGACTGACCGATAG<br>TGAACCAGTACCGTGAGGGAAAGGCGAAAAGAACCCCGGCGAGG<br>GGAGTGAAAAAGAACCTGAAACCGTGTACGTACAAGCAGTGGGAG<br>CACGCTTAGGCGTGTGACTGCGTACCTTTTGTATAATGGGTCAGC<br>GACTTATATTCTGTAGCAAGGTTAACCGAATAGGGGAGCCGAAGG<br>GAAACCGAGTCTTAACTGGGCGTTAAGTTGCAGGGTATAGACCCG<br>AAACCCGGTGATCTAGCCATGGGCAGGTTGAAGGTTGGGTAACAC<br>TAACTGGAGGACCGAACCGACTAATGTTGAAAAATTAGCGGATGAC<br>TTGTGGCTGGGGGTGAAAGGCCAATCAAACCGGGAGATAGCTGGT<br>TCTCCCCGAAAGCTATTTAGGTAGCGCCTCGTGAATTCATCTCCGG<br>GGGTAGAGCACTGTTTCGGCAAGGGGGTCATCCCGACTTACCAAC<br>CCGATGCAAACTGCGAATACCGGAGAATGTTATCACGGGAGACAC<br>ACGGCGGGTGCTAACGTCCGTCGTGAAGAGGGAAACAACCCAGA<br>CCGCCAGCTAAGGTCCCAAAGTCATGGTTAAGTGGGAAACGATGT<br>GGGAAGGCCCAGACAGCCAGGATGTTGGCTTAGAAGCAGCCATCA<br>TTTAAAGAAAGCGTAATAGCTCACTGGTCGAGTCGGCCTGCGCGG

	AAGATGTAACGGGGCTAAACCATGCACCGAAGCTGCGGCAGCGAC GCTTATGCGTTGTTGGGTAGGGGAGCGTTCTGTAAGCCTGCGAAG GTGTGCTGTGAGGCATGCTGGAGGTATCAGAAGTGCGAATGCTGA CATAAGTAACGATAAAGCGGGTGAAAAGCCCGCTCGCCGGAAGAC CAAGGGTTCCTGTCCAACGTTAATCGGGGCAGGGTGAGTCGACCC CTAAGGCGAGGCCGAAAGGCGTAGTCGATGGGAAACAGGTTAATA TTCCTGTACTTGGTGTTACTGCGAAGGGGGGACGGAGAAGGCTAT GTTGGCCGGGCGACGGTTGTCCCGGTTTAAGCGTGTAGGCTGGTT TTCCAGGCAAATCCGGAAAATCAAGGCTGAGGCGTGATGACGAGG CACTACGGTGCTGAAGCAACAAATGCCCTGCTTCCAGGAAAAGCC TCTAAGCATCAGGTAACATCAAATCGTACCCCAAACCGACACAGGT GGTCAGGTAGAGAATACCAAGGCGCTTGAGAGAACTCGGGTGAAG GAACTAGGCAAAATGGTGCCGTAACTTCGGGAGAAGGCACGCTGA TATGTAGGTGAAGCGACTTGCTCGTGGAGCTGAAATCAGTCGAAG ATACCAGCTGGCTGCAACTGTTTATTAAAAACACAGCACTGTGCAA ACACGAAAGTGGACGTATACGGTGTGACGCCTGCCCGGTGCCGG AAGGTTAATTGATGGGGTCAGCGCAAGCGAAGCTCTTGATCGAAG CCCCGGTAAACGGCGGCCGTAACTATAACGGTCCTAAGGTAGCGA AATTCCTTGTCGGGTAAGTTCCGACCTGCACGAATGGCGTAATGAT GGCCAGGCTGTCTCCACCCGAGACTCAGTGAAATTGAACTCGCTG TGAAGATGCAGTGTACCCGCGGCAAGACGGAAAGACCCCGTGAAC CTTTACTATAGCTTGACACTGAACATTGAGCCTTGATGTGTAGGAT AGGTGGGAGGCTTTGAAGTGTGGACGCCAGTCTGCATGGAGCCG

	ACCTTGAAATACCACCCTTTAATGTTTGATGTTCTAACGTGGACCC GTGATCCGGGTTGCGGACAGTGTCTGGTGGGTAGTTTGACTGGGG CGGTCTCCTCCTAAAGAGTAACGGAGGAGCACGAAGGTTGGCTAA TCCTGGTCGGACATCAGGAGGTTAGTGCAATGGCATAAGCCAGCT TGACTGCGAGCGTGACGGCGCGAGCAGGTGCGAAAGCAGGTCAT AGTGATCCGGTGGTTCTGAATGGAAGGGCCATCGCTCAACGGATA AAAGGTACTCCGGGGATAACAGGCTGATACCGCCCAAGAGTTCAT ATCGACGGCGGTGTTTGGCACCTCGATGTCGGCTCATCACATCCT GGAGCTGAAGTAGGTCCCAAGGGTATGGCTGTTCGCCATTTAAAG TGGTACGCGAGCTGGGTTTAGAACGTCGTGAGACAGTTCGGTCCC TATCTGCCGTGGGCGCTGGAGAACTGAGGGGGGCTGCTCCTAGT ACGAGAGGACCGGAGTGGACGCATCACTGGTGTTCGGGTTGTCAT GCCAATGGCACTGCCCGGTAGCTAAATGCGGAAGAGATAAGTGCT GAAAGCATCTAAGCACGAAACTTGCCCCGAGATGAGTTCTCCCTG ACTCCTTGAGAGTCCTGAAGGAACGTTGAAGACGACGACGTTGAT AGGCCGGGTGTGTAAGCGCAGCGATGCGTTGAGCTAACCGGTACT AATGAACCGTGAGGCTTAACCTT
pT7_rrnBDB:	GGTTAAGCGACTAAGCGTACACGGTGGATGCCCTGGCAGTCAGAG GCGATGAAGGACGTGCTAATCTGCGATAAGCGTCGGTAAGGTGAT ATGAACCGTTATAACCGGCGATTTCCGAATGGGGAAACCCAGTGT GTTTCGACACACTATCATTAACTGAATCCATAGGTTAATGAGGCGA

	ACCGGGGGAACTGAAACATCTAAGTACCCCGAGGAAAAGAAATCA<br>ACCGAGATTCCCCCAGTAGCGGCGAGCGAACGGGGAGCAGCCCA<br>GAGCCTGAATCAGTGTGTGTGTTAGTGGAAGCGTCTGGAAAGGCG<br>CGCGATACAGGGTGACAGCCCCGTACACAAAAATGCACATGCTGT<br>GAGCTCGATGAGTAGGGCGGGACACGTGGTATCCTGTCTGAATAT<br>GGGGGGACCATCCTCCAAGGCTAAATACTCCTGACTGACCGATAG<br>TGAACCAGTACCGTGAGGGAAAGGCGAAAAGAACCCCGGCGAGG<br>GGAGTGAAAAAGAACCTGAAACCGTGTACGTACAAGCAGTGGGAG<br>CACGCTTAGGCGTGTGACTGCGTACCTTTTGTATAATGGGTCAGC<br>GACTTATATTCTGTAGCAAGGTTAACCGAATAGGGGAGCCGAAGG<br>GAAACCGAGTCTTAACTGGGCGTTAAGTTGCAGGGTATAGACCCG<br>AAACCCGGTGATCTAGCCATGGGCAGGTTGAAGGTTGGGTAACAC<br>TAACTGGAGGACCGAACCGACTAATGTTGAAAAATTAGCGGATGAC<br>TTGTGGCTGGGGGTGAAAGGCCAATCAAACCGGGAGATAGCTGGT<br>TCTCCCCGAAAGCTATTTAGGTAGCGCCTCGTGAATTCATCTCCGG<br>GGGTAGAGCACTGTTTCGGCAAGGGGGTCATCCCGACTTACCAAC<br>CCGATGCAAACTGCGAATACCGGAGAATGTTATCACGGGAGACAC<br>ACGGCGGGTGCTAACGTCCGTCGTGAAGAGGGAAACAACCCAGA<br>CCGCCAGCTAAGGTCCCAAAGTCATGGTTAAGTGGGAAACGATGT<br>GGGAAGGCCCAGACAGCCAGGATGTTGGCTTAGAAGCAGCCATCA<br>TTTAAAGAAAGCGTAATAGCTCACTGGTCGAGTCGGCCTGCGCGG<br>AAGATGTAACGGGGCTAAACCATGCACCGAAGCTGCGGCAGCGAC<br>GCTTATGCGTTGTTGGGTAGGGGAGCGTTCTGTAAGCCTGCGAAG

	GTGTGCTGTGAGGCATGCTGGAGGTATCAGAAGTGCGAATGCTGA CATAAGTAACGATAAAGCGGGTGAAAAGCCCGCTCGCCGGAAGAC CAAGGGTTCCTGTCCAACGTTAATCGGGGCAGGGTGAGTCGACCC CTAAGGCGAGGCCGAAAGGCGTAGTCGATGGGAAACAGGTTAATA TTCCTGTACTTGGTGTTACTGCGAAGGGGGGACGGAGAAGGCTAT GTTGGCCGGGCGACGGTTGTCCCGGTTTAAGCGTGTAGGCTGGTT TTCCAGGCAAATCCGGAAAATCAAGGCTGAGGCGTGATGACGAGG CACTACGGTGCTGAAGCAACAAATGCCCTGCTTCCAGGAAAAGCC TCTAAGCATCAGGTAACATCAAATCGTACCCCAAACCGACACAGGT GGTCAGGTAGAGAATACCAAGGCGCTTGAGAGAACTCGGGTGAAG GAACTAGGCAAAATGGTGCCGTAACTTCGGGAGAAGGCACGCTGA TATGTAGGTGAAGCGACTTGCTCGTGGAGCTGAAATCAGTCGAAG ATACCAGCTGGCTGCAACTGTTTATTAAAAACACAGCACTGTGCAA ACACGAAAGTGGACGTATACGGTGTGACGCCTGCCCGGTGCCGG AAGGTTAATTGATGGGGTTAGCGCAAGCGAAGCTCTTGATCGAAG CCCCGGTAAACGGCGGCCGTAACTATAACGGTCCTAAGGTAGCGA AATTCCTTGTCGGGTAAGTTCCGACCTGCACGAATGGCGTAATGAT GGCCAGGCTGTCTCCACCCGAGACTCAGTGAAATTGAACTCGCTG TGAAGATGCAGTGTACCCGCGGCAAGACGGAAAGACCCCGTGAAC CTTTACTATAGCTTGACACTGAACATTGAGCCTTGATGTGTAGGAT AGGTGGGAGGCTTAGAAGTGTGGACGCCAGTCTGCATGGAGCCG ACCTTGAAATACCACCCTTTAATGTTTGATGTTCTAACGTTGACCCG TAATCCGGGTTGCGGACAGTGTCTGGTGGGTAGTTTGACTGGGGC

	GGTCTCCTCCTAAAGAGTAACGGAGGAGCACGAAGGTTGGCTAAT CCTGGTCGGACATCAGGAGGTTAGTGCAATGGCATAAGCCAGCTT GACTGCGAGCGTGACGGCGCGAGCAGGTGCGAAAGCAGGTCATA GTGATCCGGTGGTTCTGAATGGAAGGGCCATCGCTCAACGGATAA AAGGTACTCCGGGGATAACAGGCTGATACCGCCCAAGAGTTCATA TCGACGGCGGTGTTTGGCACCTCGATGTCGGCTCATCACATCCTG GGGCTGAAGTAGGTCCCAAGGGTGATGCTGTTCGCCATTTAAAGT GGTACGCGAGCTGGGTTTAGAACGTCGTGAGACAGTTCGGTCCCT ATCTGCCGTGGGCGCTGGAGAACTGAGGGGGGCTGCTCCTAGTA CGAGAGGACCGGAGTGGACGCATCACTGGTGTTCGGGTTGTCATG CCAATGGCACTGCCCGGTAGCTAAATGCGGAAGAGATAAGTGCTG AAAGCATCTAAGCACGAAACTTGCCCCGAGATGAGTTCTCCCTGAC CCTTTAAGGGTCCTGAAGGAACGTTGAAGACGACGACGTTGATAG GCCGGGTGTGTAAGCGCAGCGATGCGTTGAGCTAACCGGTACTAA TGAACCGTGAGGCTTAACCTT
pT7_rrnBEB:	GGTTAAGCGACTAAGCGTACACGGTGGATGCCCTGGCAGTCAGAG GCGATGAAGGACGTGCTAATCTGCGATAAGCGTCGGTAAGGTGAT ATGAACCGTTATAACCGGCGATTTCCGAATGGGGAAACCCAGTGT GTTTCGACACACTATCATTAACTGAATCCATAGGTTAATGAGGCGA ACCGGGGGAACTGAAACATCTAAGTACCCCGAGGAAAAGAAATCA

	ACCGAGATTCCCCCAGTAGCGGCGAGCGAACGGGGAGCAGCCCA<br>GAGCCTGAATCAGTGTGTGTGTTAGTGGAAGCGTCTGGAAAGGCG<br>CGCGATACAGGGTGACAGCCCCGTACACAAAAATGCACATGCTGT<br>GAGCTCGATGAGTAGGGCGGGACACGTGGTATCCTGTCTGAATAT<br>GGGGGGACCATCCTCCAAGGCTAAATACTCCTGACTGACCGATAG<br>TGAACCAGTACCGTGAGGGAAAGGCGAAAAGAACCCCGGCGAGG<br>GGAGTGAAAAAGAACCTGAAACCGTGTACGTACAAGCAGTGGGAG<br>CACGCTTAGGCGTGTGACTGCGTACCTTTTGTATAATGGGTCAGC<br>GACTTATATTCTGTAGCAAGGTTAACCGAATAGGGGAGCCGAAGG<br>GAAACCGAGTCTTAACTGGGCGTTAAGTTGCAGGGTATAGACCCG<br>AAACCCGGTGATCTAGCCATGGGCAGGTTGAAGGTTGGGTAACAC<br>TAACTGGAGGACCGAACCGACTAATGTTGAAAAATTAGCGGATGAC<br>TTGTGGCTGGGGGTGAAAGGCCAATCAAACCGGGAGATAGCTGGT<br>TCTCCCCGAAAGCTATTTAGGTAGCGCCTCGTGAATTCATCTCCGG<br>GGGTAGAGCACTGTTTCGGCAAGGGGGTCATCCCGACTTACCAAC<br>CCGATGCAAACTGCGAATACCGGAGAATGTTATCACGGGAGACAC<br>ACGGCGGGTGCTAACGTCCGTCGTGAAGAGGGAAACAACCCAGA<br>CCGCCAGCTAAGGTCCCAAAGTCATGGTTAAGTGGGAAACGATGT<br>GGGAAGGCCCAGACAGCCAGGATGTTGGCTTAGAAGCAGCCATCA<br>TTTAAAGAAAGCGTAATAGCTCACTGGTCGAGTCGGCCTGCGCGG<br>AAGATGTAACGGGGCTAAACCATGCACCGAAGCTGCGGCAGCGAC<br>GCTTATGCGTTGTTGGGTAGGGGAGCGTTCTGTAAGCCTGCGAAG<br>GTGTGCTGTGAGGCATGCTGGAGGTATCAGAAGTGCGAATGCTGA

	CATAAGTAACGATAAAGCGGGTGAAAAGCCCGCTCGCCGGAAGAC CAAGGGTTCCTGTCCAACGTTAATCGGGGCAGGGTGAGTCGACCC CTAAGGCGAGGCCGAAAGGCGTAGTCGATGGGAAACAGGTTAATA TTCCTGTACTTGGTGTTACTGCGAAGGGGGGACGGAGAAGGCTAT GTTGGCCGGGCGACGGTTGTCCCGGTTTAAGCGTGTAGGCTGGTT TTCCAGGCAAATCCGGAAAATCAAGGCTGAGGCGTGATGACGAGG CACTACGGTGCTGAAGCAACAAATGCCCTGCTTCCAGGAAAAGCC TCTAAGCATCAGGTAACATCAAATCGTACCCCAAACCGACACAGGT GGTCAGGTAGAGAATACCAAGGCGCTTGAGAGAACTCGGGTGAAG GAACTAGGCAAAATGGTGCCGTAACTTCGGGAGAAGGCACGCTGA TATGTAGGTGAGGTCCCTCGCGGATGGAGCTGAAATCAGTCGAAG ATACCAGCTGGCTGCAACTGTTTATTAAAAACACAGCACTGTGCAA ACACGAAAGTGGACGTATACGGTGTGACGCCTGCCCGGTGCCGG AAGGTTAATTGATGGGGTTAGCGCAAGCGAAGCTCTTGATCGAAG CCCCGGTAAACGGCGGCCGTAACTATAACGGTCCTAAGGTAGCGA AATTCCTTGTCGGGTAAGTTCCGACCTGCACGAATGGCGTAATGAT GGCCAGGCTGTCTCCACCCGAGACTCAGTGAAATTGAACTCGCTG TGAAGATGCAGTGTACCCGCGGCAAGACGGAAAGACCCCGTGAAC CTTTACTATAGCTTGACACTGAACATTGAGCCTTGATGTGTAGGAT AGGTGGGAGGCTTTGAAGTGTGGACGCCAGTCTGCATGGAGCCG ACCTTGAAATACCACCCTTTAATGTTTGATGTTCTAACGTTGACCCG TAATCCGGGTTGCGGACAGTGTCTGGTGGGTAGTTTGACTGGGGC GGTCTCCTCCTAAAGAGTAACGGAGGAGCACGAAGGTTGGCTAAT

	CCTGGTCGGACATCAGGAGGTTAGTGCAATGGCATAAGCCAGCTT GACTGCGAGCGTGACGGCGCGAGCAGGTGCGAAAGCAGGTCATA GTGATCCGGTGGTTCTGAATGGAAGGGCCATCGCTCAACGGATAA AAGGTACTCCGGGGATAACAGGCTGATACCGCCCAAGAGTTCATA TCGACGGCGGTGTTTGGCACCTCGATGTCGGCTCATCACATCCTG GGGCTGAAGTAGGTCCCAAGGGTATGGCTGTTCGCCATTTAAAGT GGTACGCGAGCTGGGTTTAGAACGTCGTGAGACAGTTCGGTCCCT ATCTGCCGTGGGCGCTGGAGAACTGAGGGGGGCTGCTCCTAGTA CGAGAGGACCGGAGTGGACGCATCACTGGTGTTCGGGTTGTCATG CCAATGGCACTGCCCGGTAGCTAAATGCGGAAGAGATAAGTGCTG AAAGCATCTAAGCACGAAACTTGCCCCGAGATGAGTTCTCCCTGAC TCCTTGAGAGTCCTGAAGGAACGTTGAAGACGACGACGTTGATAG GCCGGGTGTGTAAGCGCAGCGATGCGTTGAGCTAACCGGTACTAA TGAACCGTGAGGCTTAACCTT
pT7_rrnBHB:	GGTTAAGCGACTAAGCGTACACGGTGGATGCCCTGGCAGTCAGAG GCGATGAAGGACGTGCTAATCTGCGATAAGCGTCGGTAAGGTGAT ATGAACCGTTATAACCGGCGATTTCCGAATGGGGAAACCCAGTGT GTTTCGACACACTATCATTAACTGAATCCATAGGTTAATGAGGCGA ACCGGGGGAACTGAAACATCTAAGTACCCCGAGGAAAAGAAATCA ACCGAGATTCCCCCAGTAGCGGCGAGCGAACGGGGAGGAGCCCA GAGCCTGAATCAGTGTGTGTGTTAGTGGAAGCGTCTGGAAAGGCG

	CGCGATACAGGGTGACAGCCCCGTACACAAAAATGCACATGCTGT GAGCTCGATGAGTAGGGCGGGACACGTGGTATCCTGTCTGAATAT GGGGGGACCATCCTCCAAGGCTAAATACTCCTGACTGACCGATAG TGAACCAGTACCGTGAGGGAAAGGCGAAAAGAACCCCGGCGAGG GGAGTGAAAAAGAACCTGAAACCGTGTACGTACAAGCAGTGGGAG CATGCTTAGGCGTGTGACTGCGTACCTTTTGTATAATGGGTCAGCG ACTTATATTCTGTAGCAAGGTTAACCGAATAGGGGAGCCGAAGGG AAACCGAGTCTTAACTGGGCGTTAAGTTGCAGGGTATAGACCCGA AACCCGGTGATCTAGCCATGGGCAGGTTGAAGGTTGGGTAACACT AACTGGAGGACCGAACCGACTAATGTTGAAAAATTAGCGGATGACT TGTGGCTGGGGGTGAAAGGCCAATCAAACCGGGAGATAGCTGGTT CTCCCCGAAAGCTATTTAGGTAGCGCCTCGTGAACTCATCTCCGG GGGTAGAGCACTGTTTCGGCAAGGGGGTCATCCCGACTTACCAAC CCGATGCAAACTGCGAATACCGGAGAATGTTATCACGGGAGACAC ACGGCGGGTGCTAACGTCCGTCGTGAAGAGGGAAACAACCCAGA CCGCCAGCTAAGGTCCCAAAGTCATGGTTAAGTGGGAAACGATGT GGGAAGGCCCAGACAGCCAGGATGTTGGCTTAGAAGCAGCCATCA TTTAAAGAAAGCGTAATAGCTCACTGGTCGAGTCGGCCTGCGCGG AAGATGTAACGGGGCTAAACCATGCACCGAAGCTGCGGCAGCGAC GCTTATGCGTTGTTGGGTAGGGGAGCGTTCTGTAAGCCTGTGAAG GTGTACTGTGAGGTATGCTGGAGGTATCAGAAGTGCGAATGCTGA CATAAGTAACGATAAAGCGGGTGAAAAGCCCGCTCGCCGGAAGAC CAAGGGTTCCTGTCCAACGTTAATCGGGGCAGGGTGAGTCGACCC

	CTAAGGCGAGGCCGAAAGGCGTAGTCGATGGGAAACAGGTTAATA<br>TTCCTGTACTTGGTGTTACTGCGAAGGGGGACGGAGAAGGCTAT<br>GTTGGCCGGGCGACGGTTGTCCCGGTTTAAGCGTGTAGGCTGGTT<br>TTCCAGGCAAATCCGGAAAATCAAGGCTGAGGCGTGATGACGAGG<br>CACTACGGTGCTGAAGCAACAAATGCCCTGCTTCCAGGAAAAGCC<br>TCTAAGCATCAGGTAACATCAAATCGTACCCCAAACCGACACAGGT<br>GGTCAGGTAGAGAATACCAAGGCGCTTGAGAGAACTCGGGTGAAG<br>GAACTAGGCAAAATGGTGCCGTAACTTCGGGAGAAGGCACGCTGA<br>TATGTAGGTGAAGCGACTTGCTCGTGGAGCTGAAATCAGTCGAAG<br>ATACCAGCTGGCTGCAACTGTTTATTAAAAACACAGCACTGTGCAA<br>ACACGAAAGTGGACGTATACGGTGTGACGCCTGCCCGGTGCCGG<br>AAGGTTAATTGATGGGGTTAGCGCAAGCGAAGCTCTTGATCGAAG<br>CCCCGGTAAACGGCGGCCGTAACTATAACGGTCCTAAGGTAGCGA<br>AATTCCTTGTCGGGTAAGTTCCGACCTGCACGAATGGCGTAATGAT<br>GGCCAGGCTGTCTCCACCCGAGACTCAGTGAAATTGAACTCGCTG<br>TGAAGATGCAGTGTACCCGCGGCAAGACGGAAAGACCCCGTGAAC<br>CTTTACTATAGCTTGACACTGAACATTGAGCCTTGATGTGTAGGAT<br>AGGTGGGAGGCTTTGAAGTGTGGACGCCAGTCTGCATGGAGCCG<br>ACCTTGAAATACCACCCTTTAATGTTTGATGTTCTAACGTTGACCCG<br>TAATCCGGGTTGCGGACAGTGTCTGGTGGGTAGTTTGACTGGGGC<br>GGTCTCCTCCTAAAGAGTAACGGAGGAGCACGAAGGTTGGCTAAT<br>CCTGGTCGGACATCAGGAGGTTAGTGCAATGGCATAAGCCAGCTT<br>GACTGCGAGCGTGACGGCGCGAGCAGGTGCGAAAGCAGGTCATA

	GTGATCCGGTGGTTCTGAATGGAAGGGCCATCGCTCAACGGATAA<br>AAGGTACTCCGGGGATAACAGGCTGATACCGCCCAAGAGTTCATA<br>TCGACGGCGGTGTTTGGCACCTCGATGTCGGCTCATCACATCCTG<br>GGGCTGAAGTAGGTCCCAAGGGTATGGCTGTTCGCCATTTAAAGT<br>GGTACGCGAGCTGGGTTTAGAACGTCGTGAGACAGTTCGGTCCCT<br>ATCTGCCGTGGGCGCTGGAGAACTGAGGGGGGCTGCTCCTAGTA<br>CGAGAGGACCGGAGTGGACGCATCACTGGTGTTCGGGTTGTCATG<br>CCAATGGCACTGCCCGGTAGCTAAATGCGGAAGAGATAAGTGCTG<br>AAAGCATCTAAGCACGAAACTTGCCCCGAGATGAGTTCTCCCTGAC<br>TCCTTGAGAGTCCTGAAGGAACGTTGAAGACGACGACGTTGATAG<br>GCCGGGTGTGTAAGCGCAGCGATGCGTTGAGCTAACCGGTACTAA<br>TGAACCGTGAGGCTTAACCTT

Table S3. Primers for cloning and sequence confirmation of rrn operon constructs.

Primer Name	Sequence	Purpose
16S.F	CATTACGAAGTTTAATTCTTTGAGCGTCAAACT	Amplify 16S sequence, with Gibson overhangs
16S.R	GAGCACTACAAAGTACGCTTCTTTAAGG	
BB_d16S.F	CCTTAAAGAAGCGTACTTTGTAGTGCTC	Amplify 23S sequence, with Gibson overhangs
23S.R	CAAAACAGCTTCGGCGTTGT	
BB_d23S.F	ACAACGCCGAAGCTGTTTTG	Amplify 5S + backbone, with Gibson overhangs
BB_d16S.R	AGTTTGACGCTCAAAGAATTAAACTTCGTAATG	
pAM_BB_F	GAACGCTCTCCTGAGTAG	Amplify pAM552 backbone, with Gibson overhangs
pAM_BB_R	CCACACAGATTGTCTGATAAATTG	
rrn_liftoff_F	CAATTTATCAGACAATCTGTGTGG	Amplify entire rrn operon to combine with pAM552 backbone
rrn_liftoff_R	CTACTCAGGAGAGCGTTC	
16S_check1FP	GAGTTTGATCATGGCTC	RT-PCR amplify part 1 of 16S
16S_check1RP	TACGCCCAGTAATTCC	
16S_check2FP	GAGTACGGCCGCAAG	RT-PCR amplify part 2 of 16S
16S_check2_RP	CGAAGGTTAAGCTACCTAC	
23S_check1FP	AAAGACCCCGTGAAC	RT-PCR amplify 23S
23S_check1RP	GGTTAAGCCTCACGG	
16S_Sanger1	TCAGATTGAACGCTGGC	Sequencing primer 16S part 1
16S_Sanger2	CAAATGAATTGACGGG	Sequencing primer 16S part 2
23S_Sanger1	CATTGAGCCTTGATGTG	Sequencing primer 23S

Table S4. Plasmid sequences for protein expression panel.

**pJL1-sfGFP**	AGATCAAAGGATCTTCTTGAGATCCTTTTTTTCTGCGCGTAATCTG<br>CTGCTTGCAAACAAAAAAACCACCGCTACCAGCGGTGGTTTGTTTG<br>CCGGATCAAGAGCTACCAACTCTTTTTCCGAAGGTAACTGGCTTCA<br>GCAGAGCGCAGATACCAAATACTGTTCTTCTAGTGTAGCCGTAGTT<br>AGGCCACCACTTCAAGAACTCTGTAGCACCGCCTACATACCTCGC<br>TCTGCTAATCCTGTTACCAGTGGCTGCTGCCAGTGGCGATAAGTC<br>GTGTCTTACCGGGTTGGACTCAAGACGATAGTTACCGGATAAGGC<br>GCAGCGGTCGGGCTGAACGGGGGGTTCGTGCACACAGCCCAGCT<br>TGGAGCGAACGACCTACACCGAACTGAGATACCTACAGCGTGAGC<br>TATGAGAAAGCGCCACGCTTCCCGAAGGGAGAAAGGCGGACAGG<br>TATCCGGTAAGCGGCAGGGTCGGAACAGGAGAGCGCACGAGGGA<br>GCTTCCAGGGGGAAACGCCTGGTATCTTTATAGTCCTGTCGGGTT<br>TCGCCACCTCTGACTTGAGCGTCGATTTTTGTGATGCTCGTCAGG<br>GGGGCGGAGCCTATGGAAAAACGCCAGCAACGCGATCCCGCGAA<br>ATTAATACGACTCACTATAGGGAGACCACAACGGTTTCCCTCTAGA<br>AATAATTTTGTTTAACTTTAAGAAGGAGATATACATATGAGCAAAGG<br>TGAAGAACTGTTTACCGGCGTTGTGCCGATTCTGGTGGAACTGGA<br>TGGCGATGTGAACGGTCACAAATTCAGCGTGCGTGGTGAAGGTGA<br>AGGCGATGCCACGATTGGCAAACTGACGCTGAAATTTATCTGCAC<br>CACCGGCAAACTGCCGGTGCCGTGGCCGACGCTGGTGACCACCC<br>TGACCTATGGCGTTCAGTGTTTTAGTCGCTATCCGGATCACATGAA<br>ACGTCACGATTTCTTTAAATCTGCAATGCCGGAAGGCTATGTGCAG

	GAACGTACGATTAGCTTTAAAGATGATGGCAAATATAAAACGCGCG<br>CCGTTGTGAAATTTGAAGGCGATACCCTGGTGAACCGCATTGAAC<br>TGAAAGGCACGGATTTTAAAGAAGATGGCAATATCCTGGGCCATAA<br>ACTGGAATACAACTTTAATAGCCATAATGTTTATATTACGGCGGATA<br>AACAGAAAAATGGCATCAAAGCGAATTTTACCGTTCGCCATAACGT<br>TGAAGATGGCAGTGTGCAGCTGGCAGATCATTATCAGCAGAATAC<br>CCCGATTGGTGATGGTCCGGTGCTGCTGCCGGATAATCATTATCT<br>GAGCACGCAGACCGTTCTGTCTAAAGATCCGAACGAAAAAGGCAC<br>GCGGGACCACATGGTTCTGCACGAATATGTGAATGCGGCAGGTAT<br>TACGTGGAGCCATCCGCAGTTCGAAAAATAAGTCGACCGGCTGCT<br>AACAAAGCCCGAAAGGAAGCTGAGTTGGCTGCTGCCACCGCTGA<br>GCAATAACTAGCATAACCCCTTGGGGCCTCTAAACGGGTCTTGAG<br>GGGTTTTTTGCTGAAAGCCAATTCTGATTAGAAAAACTCATCGAGC<br>ATCAAATGAAACTGCAATTTATTCATATCAGGATTATCAATACCATA<br>TTTTTGAAAAAGCCGTTTCTGTAATGAAGGAGAAAACTCACCGAGG<br>CAGTTCCATAGGATGGCAAGATCCTGGTATCGGTCTGCGATTCCG<br>ACTCGTCCAACATCAATACAACCTATTAATTTCCCCTCGTCAAAAAT<br>AAGGTTATCAAGTGAGAAATCACCATGAGTGACGACTGAATCCGG<br>TGAGAATGGCAAAAGCTTATGCATTTCTTTCCAGACTTGTTCAACA<br>GGCCAGCCATTACGCTCGTCATCAAAATCACTCGCATCAACCAAAC<br>CGTTATTCATTCGTGATTGCGCCTGAGCGAGACGAAATACGCGAT<br>CGCTGTTAAAAGGACAATTACAAACAGGAATCGAATGCAACCGGC<br>GCAGGAACACTGCCAGCGCATCAACAATATTTTCACCTGAATCAG

	GATATTCTTCTAATACCTGGAATGCTGTTTTCCCGGGGATCGCAGT GGTGAGTAACCATGCATCATCAGGAGTACGGATAAAATGCTTGAT GGTCGGAAGAGGCATAAATTCCGTCAGCCAGTTTAGTCTGACCAT CTCATCTGTAACATCATTGGCAACGCTACCTTTGCCATGTTTCAGA AACAACTCTGGCGCATCGGGCTTCCCATACAATCGATAGATTGTC GCACCTGATTGCCCGACATTATCGCGAGCCCATTTATACCCATATA AATCAGCATCCATGTTGGAATTTAATCGCGGCTTCGAGCAAGACGT TTCCCGTTGAATATGGCTCATAACACCCCTTGTATTACTGTTTATGT AAGCAGACAGTTTTATTGTTCATGATGATATATTTTTATCTTGTGCA ATGTAACATCAGAGATTTTGAGACACAACGTG

**pJL1-Cas9:**<br>**158.4 kDa**	AAAAAAAATCTTATAGGGGCTCTTTTATTTGACAGTGGAGAGACAG<br>CGGAAGCGACTCGTCTCAAACGGACAGCTCGTAGAAGGTATACAC<br>GTCGGAAGAATCGTATTTGTTATCTACAGGAGATTTTTTCAAATGA<br>GATGGCGAAAGTAGATGATAGTTTCTTTCATCGACTTGAAGAGTCT<br>TTTTTGGTGGAAGAAGACAAGAAGCATGAACGTCATCCTATTTTTG<br>GAAATATAGTAGATGAAGTTGCTTATCATGAGAAATATCCAACTATC<br>TATCATCTGCGAAAAAAATTGGTAGATTCTACTGATAAAGCGGATTT<br>GCGCTTAATCTATTTGGCCTTAGCGCATATGATTAAGTTTCGTGGT<br>CATTTTTTGATTGAGGGAGATTTAAATCCTGATAATAGTGATGTGGA<br>CAAACTATTTATCCAGTTGGTACAAACCTACAATCAATTATTTGAAG<br>AAAACCCTATTAACGCAAGTGGAGTAGATGCTAAAGCGATTCTTTC<br>TGCACGATTGAGTAAATCAAGACGATTAGAAAATCTCATTGCTCAG<br>CTCCCCGGTGAGAAGAAAAATGGCTTATTTGGGAATCTCATTGCTT<br>TGTCATTGGGTTTGACCCCTAATTTTAAATCAAATTTTGATTTGGCA<br>GAAGATGCTAAATTACAGCTTTCAAAAGATACTTACGATGATGATTT<br>AGATAATTTATTGGCGCAAATTGGAGATCAATATGCTGATTTGTTTT<br>TGGCAGCTAAGAATTTATCAGATGCTATTTTACTTTCAGATATCCTA<br>AGAGTAAATACTGAAATAACTAAGGCTCCCCTATCAGCTTCAATGA<br>TTAAACGCTACGATGAACATCATCAAGACTTGACTCTTTTAAAAGCT<br>TTAGTTCGACAACAACTTCCAGAAAAGTATAAAGAAATCTTTTTTGA<br>TCAATCAAAAAACGGATATGCAGGTTATATTGATGGGGGAGCTAGC<br>CAAGAAGAATTTTATAAATTTATCAAACCAATTTTAGAAAAAATGGA<br>TGGTACTGAGGAATTATTGGTGAAACTAAATCGTGAAGATTTGCTG

	CGCAAGCAACGGACCTTTGACAACGGCTCTATTCCCCATCAAATTC<br>ACTTGGGTGAGCTGCATGCTATTTTGAGAAGACAAGAAGACTTTTA<br>TCCATTTTTAAAAGACAATCGTGAGAAGATTGAAAAAATCTTGACTT<br>TTCGAATTCCTTATTATGTTGGTCCATTGGCGCGTGGCAATAGTCG<br>TTTTGCATGGATGACTCGGAAGTCTGAAGAAACAATTACCCCATGG<br>AATTTTGAAGAAGTTGTCGATAAAGGTGCTTCAGCTCAATCATTTAT<br>TGAACGCATGACAAACTTTGATAAAAATCTTCCAAATGAAAAAGTAC<br>TACCAAAACATAGTTTGCTTTATGAGTATTTTACGGTTTATAACGAA<br>TTGACAAAGGTCAAATATGTTACTGAAGGAATGCGAAAACCAGCAT<br>TTCTTTCAGGTGAACAGAAGAAAGCCATTGTTGATTTACTCTTCAAA<br>ACAAATCGAAAAGTAACCGTTAAGCAATTAAAAGAAGATTATTTCAA<br>AAAAATAGAATGTTTTGATAGTGTTGAAATTTCAGGAGTTGAAGATA<br>GATTTAATGCTTCATTAGGTACCTACCATGATTTGCTAAAAATTATT<br>AAAGATAAAGATTTTTTGGATAATGAAGAAAATGAAGATATCTTAGA<br>GGATATTGTTTTAACATTGACCTTATTTGAAGATAGGGAGATGATTG<br>AGGAAAGACTTAAAACATATGCTCACCTCTTTGATGATAAGGTGAT<br>GAAACAGCTTAAACGTCGCCGTTATACTGGTTGGGGACGTTTGTCT<br>CGAAAATTGATTAATGGTATTAGGGATAAGCAATCTGGCAAAACAA<br>TATTAGATTTTTTGAAATCAGATGGTTTTGCCAATCGCAATTTTATG<br>CAGCTGATCCATGATGATAGTTTGACATTTAAAGAAGACATTCAAA<br>AAGCACAAGTGTCTGGACAAGGCGATAGTTTACATGAACATATTGC<br>AAATTTAGCTGGTAGCCCTGCTATTAAAAAAGGTATTTTACAGACT<br>GTAAAAGTTGTTGATGAATTGGTCAAAGTAATGGGGCGGCATAAG

	CCAGAAAATATCGTTATTGAAATGGCACGTGAAAATCAGACAACTC AAAAGGGCCAGAAAAATTCGCGAGAGCGTATGAAACGAATCGAAG AAGGTATCAAAGAATTAGGAAGTCAGATTCTTAAAGAGCATCCTGT TGAAAATACTCAATTGCAAAATGAAAAGCTCTATCTCTATTATCTCC AAAATGGAAGAGACATGTATGTGGACCAAGAATTAGATATTAATCG TTTAAGTGATTATGATGTCGATCACATTGTTCCACAAAGTTTCCTTA AAGACGATTCAATAGACAATAAGGTCTTAACGCGTTCTGATAAAAA TCGTGGTAAATCGGATAACGTTCCAAGTGAAGAAGTAGTCAAAAAG ATGAAAAACTATTGGAGACAACTTCTAAACGCCAAGTTAATCACTC AACGTAAGTTTGATAATTTAACGAAAGCTGAACGTGGAGGTTTGAG TGAACTTGATAAAGCTGGTTTTATCAAACGCCAATTGGTTGAAACT CGCCAAATCACTAAGCATGTGGCACAAATTTTGGATAGTCGCATGA ATACTAAATACGATGAAAATGATAAACTTATTCGAGAGGTTAAAGTG ATTACCTTAAAATCTAAATTAGTTTCTGACTTCCGAAAAGATTTCCA ATTCTATAAAGTACGTGAGATTAACAATTACCATCATGCCCATGATG CGTATCTAAATGCCGTCGTTGGAACTGCTTTGATTAAGAAATATCC AAAACTTGAATCGGAGTTTGTCTATGGTGATTATAAAGTTTATGATG TTCGTAAAATGATTGCTAAGTCTGAGCAAGAAATAGGCAAAGCAAC CGCAAAATATTTCTTTTACTCTAATATCATGAACTTCTTCAAAACAG AAATTACACTTGCAAATGGAGAGATTCGCAAACGCCCTCTAATCGA AACTAATGGGGAAACTGGAGAAATTGTCTGGGATAAAGGGCGAGA TTTTGCCACAGTGCGCAAAGTATTGTCCATGCCCCAAGTCAATATT GTCAAGAAAACAGAAGTACAGACAGGCGGATTCTCCAAGGAGTCA

	ATTTTACCAAAAAGAAATTCGGACAAGCTTATTGCTCGTAAAAAAGA<br>CTGGGATCCAAAAAAATATGGTGGTTTTGATAGTCCAACGGTAGCT<br>TATTCAGTCCTAGTGGTTGCTAAGGTGGAAAAAGGGAAATCGAAG<br>AAGTTAAAATCCGTTAAAGAGTTACTAGGGATCACAATTATGGAAA<br>GAAGTTCCTTTGAAAAAAATCCGATTGACTTTTTAGAAGCTAAAGG<br>ATATAAGGAAGTTAAAAAAGACTTAATCATTAAACTACCTAAATATA<br>GTCTTTTTGAGTTAGAAAACGGTCGTAAACGGATGCTGGCTAGTGC<br>CGGAGAATTACAAAAAGGAAATGAGCTGGCTCTGCCAAGCAAATA<br>TGTGAATTTTTTATATTTAGCTAGTCATTATGAAAAGTTGAAGGGTA<br>GTCCAGAAGATAACGAACAAAAACAATTGTTTGTGGAGCAGCATAA<br>GCATTATTTAGATGAGATTATTGAGCAAATCAGTGAATTTTCTAAGC<br>GTGTTATTTTAGCAGATGCCAATTTAGATAAAGTTCTTAGTGCATAT<br>AACAAACATAGAGACAAACCAATACGTGAACAAGCAGAAAATATTA<br>TTCATTTATTTACGTTGACGAATCTTGGAGCTCCCGCTGCTTTTAAA<br>TATTTTGATACAACAATTGATCGTAAACGATATACGTCTACAAAAGA<br>AGTTTTAGATGCCACTCTTATCCATCAATCCATCACTGGTCTTTATG<br>AAACACGCATTGATTTGAGTCAGCTAGGAGGTGACTGAGTCGACC<br>GGCTGCTAACAAAGCCCGAAAGGAAGCTGAGTTGGCTGCTGCCAC<br>CGCTGAGCAATAACTAGCATAACCCCTTGGGGCCTCTAAACGGGT<br>CTTGAGGGGTTTTTTGCTGAAAGCCAATTCTGATTAGAAAAACTCA<br>TCGAGCATCAAATGAAACTGCAATTTATTCATATCAGGATTATCAAT<br>ACCATATTTTTGAAAAAGCCGTTTCTGTAATGAAGGAGAAAACTCA<br>CCGAGGCAGTTCCATAGGATGGCAAGATCCTGGTATCGGTCTGCG

	ATTCCGACTCGTCCAACATCAATACAACCTATTAATTTCCCCTCGTC AAAAATAAGGTTATCAAGTGAGAAATCACCATGAGTGACGACTGAA TCCGGTGAGAATGGCAAAAGCTTATGCATTTCTTTCCAGACTTGTT CAACAGGCCAGCCATTACGCTCGTCATCAAAATCACTCGCATCAAC CAAACCGTTATTCATTCGTGATTGCGCCTGAGCGAGACGAAATAC GCGATCGCTGTTAAAAGGACAATTACAAACAGGAATCGAATGCAAC CGGCGCAGGAACACTGCCAGCGCATCAACAATATTTTCACCTGAA TCAGGATATTCTTCTAATACCTGGAATGCTGTTTTCCCGGGGATCG CAGTGGTGAGTAACCATGCATCATCAGGAGTACGGATAAAATGCTT GATGGTCGGAAGAGGCATAAATTCCGTCAGCCAGTTTAGTCTGAC CATCTCATCTGTAACATCATTGGCAACGCTACCTTTGCCATGTTTC AGAAACAACTCTGGCGCATCGGGCTTCCCATACAATCGATAGATT GTCGCACCTGATTGCCCGACATTATCGCGAGCCCATTTATACCCAT ATAAATCAGCATCCATGTTGGAATTTAATCGCGGCTTCGAGCAAGA CGTTTCCCGTTGAATATGGCTCATAACACCCCTTGTATTACTGTTTA TGTAAGCAGACAGTTTTATTGTTCATGATGATATATTTTTATCTTGT GCAATGTAACATCAGAGATTTTGAGACACAACGTGAGATCAAAGGA TCTTCTTGAGATCCTTTTTTTCTGCGCGTAATCTGCTGCTTGCAAAC AAAAAAACCACCGCTACCAGCGGTGGTTTGTTTGCCGGATCAAGA GCTACCAACTCTTTTTCCGAAGGTAACTGGCTTCAGCAGAGCGCA GATACCAAATACTGTTCTTCTAGTGTAGCCGTAGTTAGGCCACCAC TTCAAGAACTCTGTAGCACCGCCTACATACCTCGCTCTGCTAATCC TGTTACCAGTGGCTGCTGCCAGTGGCGATAAGTCGTGTCTTACCG

	GGTTGGACTCAAGACGATAGTTACCGGATAAGGCGCAGCGGTCG<br>GGCTGAACGGGGGGTTCGTGCACACAGCCCAGCTTGGAGCGAAC<br>GACCTACACCGAACTGAGATACCTACAGCGTGAGCTATGAGAAAG<br>CGCCACGCTTCCCGAAGGGAGAAAGGCGGACAGGTATCCGGTAA<br>GCGGCAGGGTCGGAACAGGAGAGCGCACGAGGGAGCTTCCAGG<br>GGGAAACGCCTGGTATCTTTATAGTCCTGTCGGGTTTCGCCACCT<br>CTGACTTGAGCGTCGATTTTTGTGATGCTCGTCAGGGGGGCGGAG<br>CCTATGGAAAAACGCCAGCAACGCGATCCCGCGAAATTAATACGA<br>CTCACTATAGGGAGACCACAACGGTTTCCCTCTAGAAATAATTTTG<br>TTTAACTTTAAGAAGGAGATATACATATGGATAAGAAATACTCAATA<br>GGCTTAGATATCGGCACAAATAGCGTCGGATGGGCGGTGATCACT<br>GATGAATATAAGGTTCCGTCTAAAAAGTTCAAGGTTCTGGGAAATA<br>CAGACCGCCACAGTATC

**pJL1-<br>CRM197-<br>ComP:**	AAAAAAACCACCGCTACCAGCGGTGGTTTGTTTGCCGGATCAAGA<br>GCTACCAACTCTTTTTCCGAAGGTAACTGGCTTCAGCAGAGCGCA<br>GATACCAAATACTGTTCTTCTAGTGTAGCCGTAGTTAGGCCACCAC<br>TTCAAGAACTCTGTAGCACCGCCTACATACCTCGCTCTGCTAATCC<br>TGTTACCAGTGGCTGCTGCCAGTGGCGATAAGTCGTGTCTTACCG<br>GGTTGGACTCAAGACGATAGTTACCGGATAAGGCGCAGCGGTCG<br>GGCTGAACGGGGGGTTCGTGCACACAGCCCAGCTTGGAGCGAAC<br>GACCTACACCGAACTGAGATACCTACAGCGTGAGCTATGAGAAAG<br>CGCCACGCTTCCCGAAGGGAGAAAGGCGGACAGGTATCCGGTAA<br>GCGGCAGGGTCGGAACAGGAGAGCGCACGAGGGAGCTTCCAGG<br>GGGAAACGCCTGGTATCTTTATAGTCCTGTCGGGTTTCGCCACCT<br>CTGACTTGAGCGTCGATTTTTGTGATGCTCGTCAGGGGGGCGGAG<br>CCTATGGAAAAACGCCAGCAACGCGATCCCGCGAAATTAATACGA<br>CTCACTATAGGGAGACCACAACGGTTTCCCTCTAGAAATAATTTTG<br>TTTAACTTTAAGAAGGAGATATACATATGGGCGCTGATGATGTTGT<br>TGATTCTTCTAAATCTTTTGTGATGGAAAACTTTTCTTCGTACCACG<br>GGACTAAACCTGGTTATGTAGATTCCATTCAAAAAGGTATACAAAA<br>GCCAAAATCTGGTACACAAGGAAATTATGACGATGATTGGAAAGAA<br>TTTTATAGTACCGACAATAAATACGACGCTGCGGGATACTCTGTAG<br>ATAATGAAAACCCGCTCTCTGGAAAAGCTGGAGGCGTGGTCAAAG<br>TGACGTATCCAGGACTGACGAAGGTTCTCGCACTAAAAGTGGATA<br>ATGCCGAAACTATTAAGAAAGAGTTAGGTTTAAGTCTCACTGAACC<br>GTTGATGGAGCAAGTCGGAACGGAAGAGTTTATCAAAAGGTTCGG

	TGATGGTGCTTCGCGTGTAGTGCTCAGCCTTCCCTTCGCTGAGGG<br>GAGTTCTAGCGTTGAATATATTAATAACTGGGAACAGGCGAAAGCG<br>TTAAGCGTAGAACTTGAGATTAATTTTGAAACCCGTGGAAAACGTG<br>GCCAAGATGCGATGTATGAGTATATGGCTCAAGCCTGTGCAGGAA<br>ATCGTGTCAGGCGATCAGTAGGTAGCTCATTGTCATGCATAAATCT<br>TGATTGGGATGTCATAAGGGATAAAACTAAGACAAAGATAGAGTCT<br>TTGAAAGAGCATGGCCCTATCAAAAATAAAATGAGCGAAAGTCCCA<br>ATAAAACAGTATCTGAGGAAAAAGCTAAACAATACCTAGAAGAATT<br>TCATCAAACGGCATTAGAGCATCCTGAATTGTCAGAACTTAAAACC<br>GTTACTGGGACCAATCCTGTATTCGCTGGGGCTAACTATGCGGCG<br>TGGGCAGTAAACGTTGCGCAAGTTATCGATAGCGAAACAGCTGAT<br>AATTTGGAAAAGACAACTGCTGCTCTTTCGATACTTCCTGGTATCG<br>GTAGCGTAATGGGCATTGCAGACGGTGCCGTTCACCACAATACAG<br>AAGAGATAGTGGCACAATCAATAGCTTTATCATCTTTAATGGTTGCT<br>CAAGCTATTCCATTGGTAGGAGAGCTAGTTGATATTGGTTTCGCTG<br>CATATAATTTTGTAGAGAGTATTATCAATTTATTTCAAGTAGTTCATA<br>ATTCGTATAATCGTCCCGCGTATTCTCCGGGGCATAAAACGCAACC<br>ATTTCTTCATGACGGGTATGCTGTCAGTTGGAACACTGTTGAAGAT<br>TCGATAATCCGAACTGGTTTTCAAGGGGAGAGTGGGCACGACATA<br>AAAATTACTGCTGAAAATACCCCGCTTCCAATCGCGGGTGTCCTAC<br>TACCGACTATTCCTGGAAAGCTGGACGTTAATAAGTCCAAGACTCA<br>TATTTCCGTAAATGGTCGGAAAATAAGGATGCGTTGCAGAGCTATA<br>GACGGTGATGTAACTTTTTGTCGCCCTAAATCTCCTGTTTATGTTG

	GTAATGGTGTGCATGCGAATCTTCACGTGGCATTTCACAGAAGCA
	GCTCGGAGAAAATTCATTCTAATGAAATTTCATCGGATTCCATAGG
	CGTTCTTGGGTACCAGAAAACAGTAGATCACACCAAGGTTAATTCT
	AAGCTATCGCTATTTTTTGAAATCAAAAGCCTCGAGGGTGGTGGTA
	GCGCATATACGGATTATACGGTTCGTTCGCGTGTTACTGAGGGTTT
	AACAACGGCATCCGCCATGAAGGCAACAGTTTCCGAGAATATCAT
	GAATGCAGGTGGGACTAGTATGCCAAGTTCCGGGAATTGTACAGG
	TGTTACGCAAATCGCCTCAGGGGCCTCGGCCGCCACTACGAATGT
	TGCCTCGGCTCAATGTTCTGACTCCGACGGGGTCATCACTGTTAC
	AATGACGGACAAGGCCAAGGGTGTTTCAATCAAGTTAACACCTTCT
	TTCTCTTCAACAGGATCAGTTGGTTGGAAGTGTACAACATCGTCAG
	ACAAGAAGTACGTTCCATCCGAGTGTCGTGGTACTGTCGACCATC
	ACCATCATCACCATTAAGTCGACCGGCTGTAACAAAGCCCGAAAG
	GAAGCTGAGTTGGCTGCTGCCACCGCTGAGCAATAACTAGCATAA
	CCCCTTGGGGCCTCTAAACGGGTCTTGAGGGGTTTTTTGCTGAAA
	GCCAATTCTGATTAGAAAAACTCATCGAGCATCAAATGAAACTGCA
	ATTTATTCATATCAGGATTATCAATACCATATTTTTGAAAAAGCCGT
	TTCTGTAATGAAGGAGAAAACTCACCGAGGCAGTTCCATAGGATG
	GCAAGATCCTGGTATCGGTCTGCGATTCCGACTCGTCCAACATCA
	ATACAACCTATTAATTTCCCCTCGTCAAAAATAAGGTTATCAAGTGA
	GAAATCACCATGAGTGACGACTGAATCCGGTGAGAATGGCAAAAG
	CTTATGCATTTCTTTCCAGACTTGTTCAACAGGCCAGCCATTACGC
	TCGTCATCAAAATCACTCGCATCAACCAAACCGTTATTCATTCGTG

	ATTGCGCCTGAGCGAGACGAAATACGCGATCGCTGTTAAAAGGAC<br>AATTACAAACAGGAATCGAATGCAACCGGCGCAGGAACACTGCCA<br>GCGCATCAACAATATTTTCACCTGAATCAGGATATTCTTCTAATACC<br>TGGAATGCTGTTTTCCCGGGGATCGCAGTGGTGAGTAACCATGCA<br>TCATCAGGAGTACGGATAAAATGCTTGATGGTCGGAAGAGGCATA<br>AATTCCGTCAGCCAGTTTAGTCTGACCATCTCATCTGTAACATCATT<br>GGCAACGCTACCTTTGCCATGTTTCAGAAACAACTCTGGCGCATC<br>GGGCTTCCCATACAATCGATAGATTGTCGCACCTGATTGCCCGAC<br>ATTATCGCGAGCCCATTTATACCCATATAAATCAGCATCCATGTTG<br>GAATTTAATCGCGGCTTCGAGCAAGACGTTTCCCGTTGAATATGGC<br>TCATAACACCCCTTGTATTACTGTTTATGTAAGCAGACAGTTTTATT<br>GTTCATGATGATATATTTTTATCTTGTGCAATGTAACATCAGAGATT<br>TTGAGACACAACGTGAGATCAAAGGATCTTCTTGAGATCCTTTTTTT<br>CTGCGCGTAATCTGCTGCTTGCAAAC

**pJL1-TRI2-2:**	AAAAAAACCACCGCTACCAGCGGTGGTTTGTTTGCCGGATCAAGA<br>GCTACCAACTCTTTTTCCGAAGGTAACTGGCTTCAGCAGAGCGCA<br>GATACCAAATACTGTTCTTCTAGTGTAGCCGTAGTTAGGCCACCAC<br>TTCAAGAACTCTGTAGCACCGCCTACATACCTCGCTCTGCTAATCC<br>TGTTACCAGTGGCTGCTGCCAGTGGCGATAAGTCGTGTCTTACCG<br>GGTTGGACTCAAGACGATAGTTACCGGATAAGGCGCAGCGGTCG<br>GGCTGAACGGGGGGTTCGTGCACACAGCCCAGCTTGGAGCGAAC<br>GACCTACACCGAACTGAGATACCTACAGCGTGAGCTATGAGAAAG<br>CGCCACGCTTCCCGAAGGGAGAAAGGCGGACAGGTATCCGGTAA<br>GCGGCAGGGTCGGAACAGGAGAGCGCACGAGGGAGCTTCCAGG<br>GGGAAACGCCTGGTATCTTTATAGTCCTGTCGGGTTTCGCCACCT<br>CTGACTTGAGCGTCGATTTTTGTGATGCTCGTCAGGGGGGCGGAG<br>CCTATGGAAAAACGCCAGCAACGCGATCCCGCGAAATTAATACGA<br>CTCACTATAGGGAGACCACAACGGTTTCCCTCTAGAAATAATTTTG<br>TTTAACTTTAAGAAGGAGATATACATATGGAGAAAAAAATCGAGTTA<br>GAAGAACAAGTTATGCACGTACTGGACCAGGTCAGCGAACTCGCG<br>CATGAATTGCTGCACAAACTGACCGGCGAAGAATTGGAGCGCGCA<br>GCGTACTTCAACTGGTGGGCAACCGAAATGATGCTGGAGCTGATT<br>AAATCTGACGACGAACGCGAAATTCGTGAGATCGAAGAGGAGGCT<br>CGCCGTATTCTGGAGCACCTGGAAGAACTTGCGCGTAAAGGGGG<br>GTCCGAAGCCCTGGAAGAATTGGAAAAAGCACTTCGCGAATTAAA<br>AAAATCCACAGACGAACTTGAGCGCTCTACAGAGGAGTTAGAGAA

	GAATCCATCCGAAGACGCTTTGGTCGAGAATAATCGCTTGATCGTA<br>GAAAACAATAAGATCATCGTAGAGGTTCTGCGTATTATCGCCAAAG<br>TCTTAAAGGGTGGCGCTTCTCCTGCTGCACCTGCTCCTGGAGGCG<br>ACTACAAAGATGAAGACCTTCTTGGTGGAGCCAGTCCAGCAGCTC<br>CTGCGCCTGGCGGGTCCGCATGGTCCCACCCTCAATTTGAAAAAT<br>AAGTCGACCGGCTGCTAACAAAGCCCGAAAGGAAGCTGAGTTGGC<br>TGCTGCCACCGCTGAGCAATAACTAGCATAACCCCTTGGGGCCTC<br>TAAACGGGTCTTGAGGGGTTTTTTGCTGAAAGCCAATTCTGATTAG<br>AAAAACTCATCGAGCATCAAATGAAACTGCAATTTATTCATATCAGG<br>ATTATCAATACCATATTTTTGAAAAAGCCGTTTCTGTAATGAAGGAG<br>AAAACTCACCGAGGCAGTTCCATAGGATGGCAAGATCCTGGTATC<br>GGTCTGCGATTCCGACTCGTCCAACATCAATACAACCTATTAATTT<br>CCCCTCGTCAAAAATAAGGTTATCAAGTGAGAAATCACCATGAGTG<br>ACGACTGAATCCGGTGAGAATGGCAAAAGCTTATGCATTTCTTTCC<br>AGACTTGTTCAACAGGCCAGCCATTACGCTCGTCATCAAAATCACT<br>CGCATCAACCAAACCGTTATTCATTCGTGATTGCGCCTGAGCGAG<br>ACGAAATACGCGATCGCTGTTAAAAGGACAATTACAAACAGGAATC<br>GAATGCAACCGGCGCAGGAACACTGCCAGCGCATCAACAATATTT<br>TCACCTGAATCAGGATATTCTTCTAATACCTGGAATGCTGTTTTCCC<br>GGGGATCGCAGTGGTGAGTAACCATGCATCATCAGGAGTACGGAT<br>AAAATGCTTGATGGTCGGAAGAGGCATAAATTCCGTCAGCCAGTTT<br>AGTCTGACCATCTCATCTGTAACATCATTGGCAACGCTACCTTTGC<br>CATGTTTCAGAAACAACTCTGGCGCATCGGGCTTCCCATACAATCG

	ATAGATTGTCGCACCTGATTGCCCGACATTATCGCGAGCCCATTTA<br>TACCCATATAAATCAGCATCCATGTTGGAATTTAATCGCGGCTTCG<br>AGCAAGACGTTTCCCGTTGAATATGGCTCATAACACCCCTTGTATT<br>ACTGTTTATGTAAGCAGACAGTTTTATTGTTCATGATGATATATTTTT<br>ATCTTGTGCAATGTAACATCAGAGATTTTGAGACACAACGTGAGAT<br>CAAAGGATCTTCTTGAGATCCTTTTTTTCTGCGCGTAATCTGCTGC<br>TTGCAAAC

**pJL1-MS2:**	AAAAAAACCACCGCTACCAGCGGTGGTTTGTTTGCCGGATCAAGA<br>GCTACCAACTCTTTTTCCGAAGGTAACTGGCTTCAGCAGAGCGCA<br>GATACCAAATACTGTTCTTCTAGTGTAGCCGTAGTTAGGCCACCAC<br>TTCAAGAACTCTGTAGCACCGCCTACATACCTCGCTCTGCTAATCC<br>TGTTACCAGTGGCTGCTGCCAGTGGCGATAAGTCGTGTCTTACCG<br>GGTTGGACTCAAGACGATAGTTACCGGATAAGGCGCAGCGGTCG<br>GGCTGAACGGGGGGTTCGTGCACACAGCCCAGCTTGGAGCGAAC<br>GACCTACACCGAACTGAGATACCTACAGCGTGAGCTATGAGAAAG<br>CGCCACGCTTCCCGAAGGGAGAAAGGCGGACAGGTATCCGGTAA<br>GCGGCAGGGTCGGAACAGGAGAGCGCACGAGGGAGCTTCCAGG<br>GGGAAACGCCTGGTATCTTTATAGTCCTGTCGGGTTTCGCCACCT<br>CTGACTTGAGCGTCGATTTTTGTGATGCTCGTCAGGGGGGCGGAG<br>CCTATGGAAAAACGCCAGCAACGCGATCCCGCGAAATTAATACGA<br>CTCACTATAGGGAGACCACAACGGTTTCCCTCTAGAAATAATTTTG<br>TTTAACTTTAAGAAGGAGATATACATAATGGCTTCTAACTTTACTCA<br>GTTCGTTCTCGTCGACAATGGCGGAACTGGCGACGTGACTGTCGC<br>CCCAAGCAACTTCGCTAACGGGGTCGCTGAATGGATCAGCTCTAA<br>CTCGCGTTCACAGGCTTACAAAGTAACCTGTAGCGTTCGTCAGAG<br>CTCTGCGCAGAATCGCAAATACACCATCAAAGTCGAGGTGCCTAA<br>AGTGGCAACCCAGACTGTTGGTGGTGTAGAGCTTCCTGTAGCCGC<br>ATGGCGTTCGTACTTAAATATGGAACTAACCATTCCAATTTTCGCTA<br>CGAATTCCGACTGCGAGCTTATTGTTAAGGCGATGCAAGGTCTCC

	TAAAAGATGGAAACCCGATTCCCTCAGCAATCGCAGCAAACTCCG<br>GCATCTACTAAAGTCGACCGGCTGCTAACAAAGCCCGAAAGGAAG<br>CTGAGTTGGCTGCTGCCACCGCTGAGCAATAACTAGCATAACCCC<br>TTGGGGCTTCTAAACGGGTCTTGAGGGGTTTTTTGCTGAAAGCCA<br>ATTCTGATTAGAAAAACTCATCGAGCATCAAATGAAACTGCAATTTA<br>TTCATATCAGGATTATCAATACCATATTTTTGAAAAAGCCGTTTCTG<br>TAATGAAGGAGAAAACTCACCGAGGCAGTTCCATAGGATGGCAAG<br>ATCCTGGTATCGGTCTGCGATTCCGACTCGTCCAACATCAATACAA<br>CCTATTAATTTCCCCTCGTCAAAAATAAGGTTATCAAGTGAGAAATC<br>ACCATGAGTGACGACTGAATCCGGTGAGAATGGCAAAAGCTTATG<br>CATTTCTTTCCAGACTTGTTCAACAGGCCAGCCATTACGCTCGTCA<br>TCAAAATCACTCGCATCAACCAAACCGTTATTCATTCGTGATTGCG<br>CCTGAGCGAGACGAAATACGCGATCGCTGTTAAAAGGACAATTAC<br>AAACAGGAATCGAATGCAACCGGCGCAGGAACACTGCCAGCGCAT<br>CAACAATATTTTCACCTGAATCAGGATATTCTTCTAATACCTGGAAT<br>GCTGTTTTCCCGGGGATCGCAGTGGTGAGTAACCATGCATCATCA<br>GGAGTACGGATAAAATGCTTGATGGTCGGAAGAGGCATAAATTCC<br>GTCAGCCAGTTTAGTCTGACCATCTCATCTGTAACATCATTGGCAA<br>CGCTACCTTTGCCATGTTTCAGAAACAACTCTGGCGCATCGGGCTT<br>CCCATACAATCGATAGATTGTCGCACCTGATTGCCCGACATTATCG<br>CGAGCCCATTTATACCCATATAAATCAGCATCCATGTTGGAATTTAA<br>TCGCGGCTTCGAGCAAGACGTTTCCCGTTGAATATGGCTCATAAC<br>ACCCCTTGTATTACTGTTTATGTAAGCAGACAGTTTTATTGTTCATG

	ATGATATATTTTTATCTTGTGCAATGTAACATCAGAGATTTTGAGAC<br>ACAACGTGAGATCAAAGGATCTTCTTGAGATCCTTTTTTTCTGCGC<br>GTAATCTGCTGCTTGCAAAC

Table S5. Growth data of all strains included in this study. Lag time and doubling times shown. Data represent mean and standard deviation (SD) of n=7 replicates. Replicates containing outliers that fell >2 standard deviations from the median were excluded from analysis.

Strain	Lag time (hrs)	SD	Doubling Time (hrs)	SD
SQ-AAA	**3.40**	0.14	**1.19**	0.01
SQ-ABB	**3.13**	0.15	**1.08**	0.00
SQ-BBB	**2.78**	0.07	**1.05**	0.00
SQ-BCB	**2.78**	0.04	**1.07**	0.00
WT - BL21(DE3)*	**1.31**	0.02	**0.66**	0.00
SQ-CCC	**3.21**	0.18	**1.00**	0.01
SQ-DBB	**3.24**	0.13	**1.05**	0.01
SQ-EEE	**2.96**	0.07	**1.07**	0.00
SQ-GGG	**3.15**	0.04	**1.13**	0.00
SQ-HBB	**3.24**	0.03	**1.11**	0.00
WT - MG1655	**1.28**	0.02	**0.69**	0.01
