# Supplementary material for: Ribosome Pool Engineering Increases Protein Biosynthesis Yields
Source: ACS Cent Sci. 2024 Mar 20;10(4):871–81. doi: 10.1021/acscentsci.3c01413 (PMC11046459; doi:10.1021/acscentsci.3c01413)
Supplement: Supplementary file 2 — oc3c01413_si_002.pdf [file oc3c01413_si_002.pdf]

oc-2023-01413u.R1

Name: Peer Review Information for "Ribosome pool engineering increases protein biosynthesis yields"

First Round of Reviewer Comments

Reviewer: 1

Comments to the Author

This study aims to investigate the functions of ribosomes in affecting protein yields. The authors creatively explored various combinations of 16S and 23S rRNAs in the in vitro translation platform. Some pretty interesting results have been observed. These findings might promote an understanding of life systems, and they also could provide rational strategies for protein engineering. As currently presented, a few points need to be further addressed.

1. The description of the significance of this study at the basic science and application levels is not very clear. Please describe more clearly。

2. iSAT enables the activation of rRNA transcription. Have the authors quantified the rRNA or ribosome amounts?

3. The rRNAs or ribosomes are easily degraded. How did the authors prevent the degradation in the testing system?

4. Different 16S:23S:5S combinations demonstrated various results. Suggest that the authors draw a diagram clarifying the action mechanism of interactions between 16S, 23S, and 5S.

5. The rRNA operons are primarily the same in sequence but differ by 21 unique point mutants in the 16S rRNA, 34 in the 23S rRNA, and 3 in the 5S rRNA. How these mutations affect the functions needs to be further clarified.

Reviewer: 2

Comments to the Author

Comments:

This manuscript by Camila Kofman, et al. reveals the existence of functional activity variation among ribosomes originating from the seven rRNA operons within native ribosome pools in E. coli.

The authors introduce the notion of ribosome pool engineering, demonstrating that certain rRNA sequences can significantly enhance the yield of bulk protein biosynthesis. From a new perspective on effects of different rRNA sequences, this study provides new ideals for optimizing the efficiency and yields of CFPS. However, prior publications from this research group involving the design and screening of novel rRNA to enhance ribosomal efficiency are already in existence. (See Nucleic Acids Research, 2022, 50(22): 13143-13154 and Nature Communications, 2023, 14(1): 961.) This current work appears to be a very basic application of ISAT technology and lacks sufficient novelty, with the efficacy of the application being suboptimal comparing to previous works. Therefore, I find that the manuscript does not align sufficiently with the level of advance for ACS Central Science.

1. In figure 1C, the colors of different operons are too similar, making it difficult to correspond them individually.

2. Line 5 on page 8, rrnD and H sequences are unable to support life while figure 2B shows rrnH can support strain growth. This is very confusing.

3. In figure 1C and figure 2D, why is the relative activity of different operons inconsistent between the two figures? For example, rrnA and G exhibit similar activities in figure 1C, but in Figure 2D, there is a significant difference in their activities. Given that the reaction conditions are largely the same, why would this lead to such significant variations.

4. Still in figure 2, why is there a significant difference in the activities of rrnA and C, but their impact on cell growth is minimal as shown in figure 2B?

5. Further details on the growth of strains containing single operon need to be characterized such as growth curve. Does higher operon activity correlate with better growth of the strain? Will strains containing only rrnA exhibit increased protein expression yield?

6. Most importantly, the effect of using single operon derived ribosomes pools to enhance protein yield is not significant. Is it feasible to design and screen for rRNA sequences with enhanced activity?

Reviewer: 3

Comments to the Author

This manuscript describes an investigation of the seven distinct genomic rRNA operons from E. coli MG1655 and the performance of the ribosomes encoded in these operons in supporting protein synthesis. In single-operon iSAT assays, the operons support varying levels of GFP synthesis. Attempts to transform a ribosome-deficient E. coli strain with plasmids encoding one of the operons led to successful transformation and proliferation of 5 out of 7 single operons. GFP synthesis using purified single-operon ribosomes showed that multiple 70s ribosome variants

support higher levels of GFP synthesis than the pooled ribosomes from MG1655. Mutations at several helices within the ribosomes were studies systematically and shown to exhibit further variability in the levels of GFP synthesis observed. Cell-free protein synthesis studies with a single operon ribosome led to higher yields of GFP for several variants, and for the AAA operon ribosome, several proteins were expressed at higher levels in comparison to the control MG1655 pool of ribosomes. Additional assays using the single-operon ribosome lysate yielded positive functional readouts, further confirming successful protein synthesis with single operon ribosomes in vitro.

Overall, this study is an important study and has implications for biochemistry, synthetic biology, and biomanufacturing. This study is extremely interesting because it indicates that ribosomes encoded by different genomic rRNA operons from E. coli exhibit substantially different behaviors under the conditions studied. This in itself is an important and surprising finding. The additional investigations of single operon ribosome properties both in in vitro protein synthesis assays and in cell spotting assays (to determine viability) are also intriguing. The fact that some single operons substantially outperform the wild-type ribosome pool from MG1655 and that other single operons exhibit no detectable protein synthesis is another important set of observations pointing to a diverse ribosome pool in MG1655. While this is important work, there are some concerns outlined below that the authors should address prior to publication. The concerns range from relatively substantial to relatively minor.

1. Figure 2: Panel B is labeled with operons ABCGH, but panel D is labeled with operons ABCEG, and the text states that strain development for operons D and H was not possible. This discrepancy needs to be resolved.

2. Figure 4: The AdhE2 activity quantification results are a bit puzzling. The methods state that the negative control lacks AdhE2 expression, but ~30% conversion of butanol is observed even without the added plasmid encoding the alcohol dehydrogenase. Is this level of conversion expected for the negative control? This needs to be clarified in the text.

3. “Support life” and related terminology. Laboratory cell growth and protein synthesis conditions are very narrow in relationship to the possible range of conditions that are possible in natural systems (for example, consider biofilm growth versus planktonic growth). It is not yet known whether the operons that show low or zero activity in the experiments performed in this study are similarly inactive in other conditions. Thus, the phrase, “support life” is overly broad and not accurate without additional clarification. Recommend rewording and careful consideration of language surrounding what protein synthesis and cellular proliferation is supported by different variants.

4. This study is performed predominantly in vitro, but several new E. coli strains containing only a single rRNA operon were created. It would be interesting to know the growth characteristics of these strains and how they compare to other derivatives of the SQ171 strain. Ideally, doubling times or growth curves of the newly created strains alongside additional SQ171 strain derivatives such as SQ171fg as controls would be an excellent addition to this work (spot assays may also be

appropriate). This would augment the quality of this study substantially, especially since the properties of a ribosome that make it good for high-level protein synthesis may not lead to efficient cell proliferation.

5. At the start of the results and discussion, there are two paragraphs of description of the iSAT system. Some of the text of these paragraphs reads like introductory material, especially the description of prior iSAT studies. Recommend considering moving some of this material into the introduction.

6. Figure 1C: with every operon in a different shade of green, it is hard to pinpoint which shade corresponds to which specific operon. Recommend using multiple colors to make this easier to understand. The same color scheme could be used in the “single operons” graph to the right, which might clarify things further and make the figure easier to understand.

7. Supporting information: there is an unresolved Word comment regarding plasmid pJL1-CRM197 and its sequence in relationship to the sequence found in another lab publication. The apparent discrepancy mention in the comment needs to be resolved and clarified.

Author's Response to Peer Review Comments:

Reviewer(s)' Comments to Author:
Reviewer: 1

Recommendation: Publish in ACS Central Science after minor revisions noted.

Comments:
This study aims to investigate the functions of ribosomes in affecting protein yields. The authors creatively explored various combinations of 16S and 23S rRNAs in the in vitro translation platform. Some pretty interesting results have been observed. These findings might promote an understanding of life systems, and they also could provide rational strategies for protein engineering. As currently presented, a few points need to be further addressed.

We thank you for celebrating the interesting results we observed.

1. The description of the significance of this study at the basic science and application levels is not very clear. Please describe more clearly.

We thank you for the suggestion to describe the significance of this study more clearly and have revised the abstract and discussion to do so.

In terms of basic science significance, we now directly describe that, for the first time, we:

"observe that the polymorphisms distinguishing native E. coli rRNA operons lead to significant functional changes in the resulting ribosomes, ranging from negligible or low gene expression to matching the protein production activity of the standard rRNA operon B sequence. We go on to generate strains expressing single rRNA operons and show that not only do some purified in vivo expressed homogeneous ribosome pools outperform the wildtype, heterogeneous ribosome pool, but also that a crude cell lysate made from the strain expressing only operon A ribosomes shows significant yield increases for a panel of medically and industrially relevant proteins."

In terms of application significance, we include in the final sentence of our abstract the following:

"We anticipate that ribosome pool engineering can be applied as a tool to increase yields across many protein biomanufacturing systems, as well as improve basic understanding of ribosome heterogeneity and evolution."

We further highlight these points in the final paragraph of the discussion:

"In summary, this work demonstrates new basic science knowledge that functional activity variation exists across ribosomes derived from the seven rRNA operons in natively expressed ribosome pools in E. coli. We also illustrate the concept of ribosome pool engineering and show that some rRNA sequences have increased bulk protein biosynthesis yields. We anticipate that ribosome pool engineering will enable engineering applications in common workhorse organisms and strains by optimizing the ribosome pool to contain only the most productive rRNA sequences for specific applications both in vitro and in vivo. Thus, ribosome pool engineering represents a previously overlooked dimension of optimization for maximizing industrial protein production. Looking forward, we anticipate that studying rRNA sequence-function relationships will build a deeper understanding of how ribosomes have evolved and how we might design specialized ribosomes for applications in biotechnology and synthetic biology."

2. iSAT enables the activation of rRNA transcription. Have the authors quantified the rRNA or ribosome amounts?

You are correct that iSAT co-activates multiple biological processes, including rRNA transcription, ribosome assembly, and translation of a reporter protein, in this case sfGFP. Previous work in our lab has quantified synthesized ribosome amounts to be ~100nM with the molarity of the 16S rRNAs in the iSAT reaction higher than that of the

23S rRNA[1,2]. Our previous studies have also showed consistent rRNA transcription from the T7 RNA polymerase promoter and so we did not re-quantify rRNA here[2]. To assess activity of ribosomes alone (not iSAT activity), we quantify translational activity with the more direct concentration normalization from the strain-isolated and purified ribosomes (e.g., Figure 2E).

3. The rRNAs or ribosomes are easily degraded. How did the authors prevent the degradation in the testing system?

Our system has two features that help prevent rRNA and ribosome degradation. First, the strain that we use to make S150 lysate is called MRE600, a strain that has an RNase knockout (RNase I) and thus is known for its low RNase activity. Second, during S150 extract preparation, significant amounts of RNAse inhibitor are added both before *and* after cell lysis, as described in the Methods section. The denaturing gels that were run in our NAR 2014 study[2] show clearly that there is rRNA transcribed in the iSAT reaction to stable levels.

We added the following sentence to the results section:

> *"Notably, iSAT has two features that help prevent rRNA and ribosome degradation. First, the strain that we use to make S150 lysate lacks RNase I and thus is known for its low RNase activity. Second, during S150 extract preparation, significant amounts of RNAse inhibitor are added both before and after cell lysis, as described in the Methods section."*

4. Different 16S:23S:5S combinations demonstrated various results. Suggest that the authors draw a diagram clarifying the action mechanism of interactions between 16S, 23S, and 5S.

We agree with you that drawing a diagram to clarify the mechanism of interactions between 16S, 23S, and 5S is warranted. We have thus prepared a new Supplementary Figure S1 that highlights the polymorphisms that exist between the different operon sequences in 3D space (see below). This will help the reader to understand where the differences in sequence are manifest in the ribosome's structure and how they may be affecting function.

---

[1] Brian R. Fritz, Osman K. Jamil, and Michael C. Jewett, "Implications of Macromolecular Crowding and Reducing Conditions for in Vitro Ribosome Construction," *Nucleic Acids Research* 43, no. 9 (March 2015): 4774–4784, doi:10.1093/nar/gkv329.

[2] Brian R. Fritz and Michael C. Jewett, "The Impact of Transcriptional Tuning on in Vitro Integrated RRNA Transcription and Ribosome Construction," *Nucleic Acids Research* 42, no. 10 (2014): 6774–6785, doi:10.1093/nar/gku307.

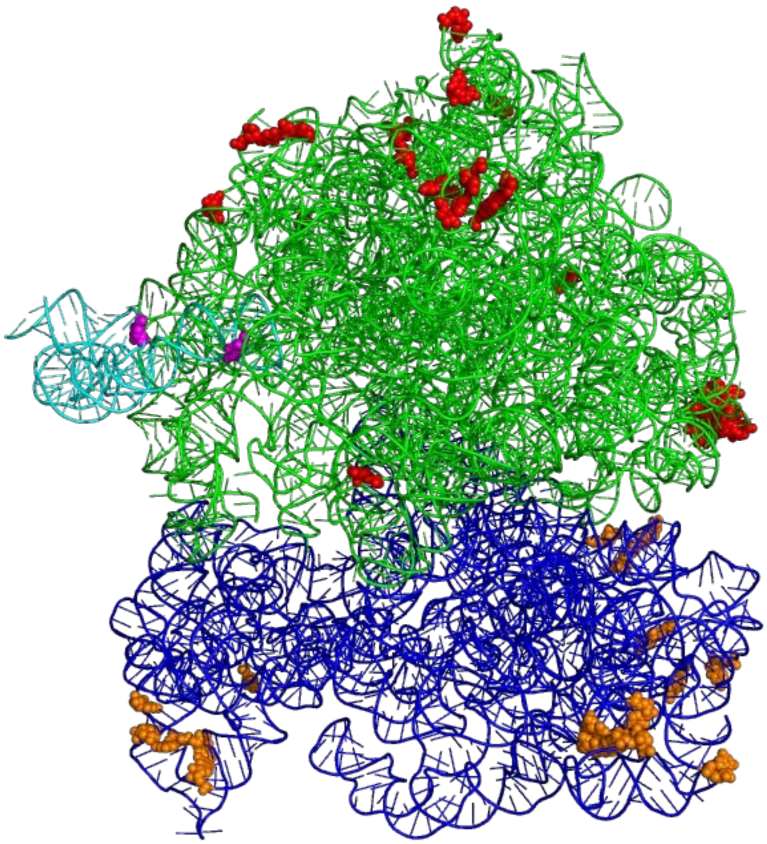

Supplementary Figure S1. Polymorphism mapping onto rRNA structure. 23S rRNA is shown in green, with polymorphisms highlighted as red spheres. 16S rRNA is shown in dark blue, with polymorphisms highlighted as orange spheres. 5S rRNA is shown in light blue, with polymorphisms highlighted as magenta spheres. Ribosomal proteins are not shown.

We also added a section in the discussion of Figure 3 to include an in-depth analysis and literature review of specific mutations that may be responsible for the changes in activity that we measured.

*"Considering the structure of the rRNA and where these polymorphisms fall in 3D space, we can infer how sequence differences between operons may affect activity (**Supplementary Figure S1**). While most of the polymorphisms sit on the outer regions of the rRNA and are thus less likely to be directly interacting with translocation processes of tRNAs through the ribosomal active site, some exist in motifs that are known to play dynamic roles in translation activity. For example, the 23S rRNA sequences of operons A, C, E, and H differ from that of B, D, and G at Helix 98 (H98), a motif that has been shown to participate in a tertiary interaction that is important for ribosome stabilization [29]. Similarly, H68, a 23S rRNA helix that is extended by a Watson-Crick (WC) base pair in the A operon, is known to be actively involved in dynamic ribosome movements that are necessary for the process of elongation via coordination with the L1 stalk and tRNAs[30]. The additional WC, which would increase the length of H68, potentially*

---

[3] Michael J. Hammerling et al., "In Vitro Ribosome Synthesis and Evolution through Ribosome Display," *Nature Communications* 11, no. 1 (2020), doi:10.1038/s41467-020-14705-2.

affects the dynamics of the interaction with the L1 stalk to alter the efficiency of the elongation process. In the 16S rRNA, operon H carries seven unique polymorphisms in helix 33 (h33), which sits in the "head" of the small subunit and plays an important role in ratcheting along the mRNA in the process of translation [31]. Additionally, many polymorphisms exist in rRNA motifs that interact closely with r-proteins. For example, h11 of operon D contains a single residue change, but is adjacent to S16, a protein that is essential for cellular viability[32]. Changes in the sequence of h11 could thus impact the interaction with S16 and potentially have deleterious effects on ribosome activity."

5. The rRNA operons are primarily the same in sequence but differ by 21 unique point mutants in the 16S rRNA, 34 in the 23S rRNA, and 3 in the 5S rRNA. How these mutations affect the functions needs to be further clarified.

We agree that we should better clarify how the mutations present in the native operons could affect ribosomal functions. In the revised manuscript, we map the polymorphisms onto the 3D ribosome structure (Supplementary Figure S1) and provide additional discussion as captured in our response to the previous point above.

Notably, we also directly explore mutations in Figure 3 (panels 3B & C), in which we studied the differences between rrnC and rrnD sequences. We found that the polymorphisms present in Helix 92 of rrnD impact function by causing a loss of iSAT activity (i.e., combined transcription, ribosome assembly, and translation).

Looking ahead, we share your interest to explore even further how each mutation affects function in a future study. We are particularly curious about not only individual polymorphisms but also combinations of polymorphisms due to the strong epistatic interactions that occur between rRNAs in the ribosome [3,4]. We have added a sentence at the end of this section in the manuscript to capture this interest:

> *"Future work to systematically elucidate all effects of individual and combinatorial polymorphisms on translational activity would improve understanding of the mechanistic and functional consequences of these distinct, natively occurring rRNA sequences."*

Additional Questions:
Quality of experimental data, technical rigor: Top 5%

Significance to chemistry researchers in this and related fields: Top 5%

Broad interest to other researchers: Top 5%

Novelty: Top 5%

---

[4] Camila Kofman et al., "Computationally-Guided Design and Selection of High Performing Ribosomal Active Site Mutants," *Nucleic Acids Research* 50, no. 22 (December 2022): 13143–13154, doi:10.1093/nar/gkac1036.

Is this research study suitable for media coverage or a First Reactions (a News & Views piece in the journal)?: Yes

Reviewer: 2

Recommendation: Reconsider after major revisions noted.

Comments:
This manuscript by Camila Kofman, et al. reveals the existence of functional activity variation among ribosomes originating from the seven rRNA operons within native ribosome pools in E. coli. The authors introduce the notion of ribosome pool engineering, demonstrating that certain rRNA sequences can significantly enhance the yield of bulk protein biosynthesis. From a new perspective on effects of different rRNA sequences, this study provides new ideals for optimizing the efficiency and yields of CFPS. However, prior publications from this research group involving the design and screening of novel rRNA to enhance ribosomal efficiency are already in existence. (See Nucleic Acids Research, 2022, 50(22): 13143-13154 and Nature Communications, 2023, 14(1): 961.) This current work appears to be a very basic application of ISAT technology and lacks sufficient novelty, with the efficacy of the application being suboptimal comparing to previous works. Therefore, I find that the manuscript does not align sufficiently with the level of advance for ACS Central Science.

We thank you for celebrating our innovation of the notion of ribosome pool engineering and highlighting how our study provides new ideas for optimizing protein expression.

Your comments also helped us realize that we need to better highlight the level of advance our manuscript presents. The advance of this study is not solely the application of iSAT to rRNA sequence characterization, but rather that we go significantly beyond what was done before to characterize naturally occurring variation between genomic rRNA operons and to see if this can be used to optimize protein biosynthesis.

Ribosomes have conventionally been thought of as uniform molecular assemblies even though most organisms carry multiple copies of unique rRNA-encoding operons (rrn) in their genomes. Surprisingly, our observations show that small rRNA polymorphisms lead to large functional consequences. As highlighted by Reviewer 3, this "indicates that ribosomes encoded by different genomic rRNA operons from *E. coli* exhibit substantially different behaviors … (which) is an important and surprising finding." We go on to introduce the concept of ribosome pool engineering. This concept is reported for the first time to our knowledge and has important implications for synthetic biology and biotechnology.

We now more clearly state the goals of the manuscript in the introduction (including that we are simply using iSAT as a method) and focus of the advance:

“we set out to use the iSAT method to explore whether heterogeneity of native rRNA sequences affects the activity of resulting ribosomes and if this can be used to optimize protein biosynthesis.”

“Our results suggest that ribosome pool engineering has the potential to improve biomanufacturing systems for many applications in synthetic biology, including cell-free protein synthesis and recombinant protein production, as well as elucidate deeper understanding of ribosome heterogeneity and evolution.”

1. In figure 1C, the colors of different operons are too similar, making it difficult to correspond them individually.

We thank you for this comment and agree that the different shades of green were difficult to differentiate. We have updated the figure to have higher contrast colors and have updated all figures in the manuscript to match the new color scheme.

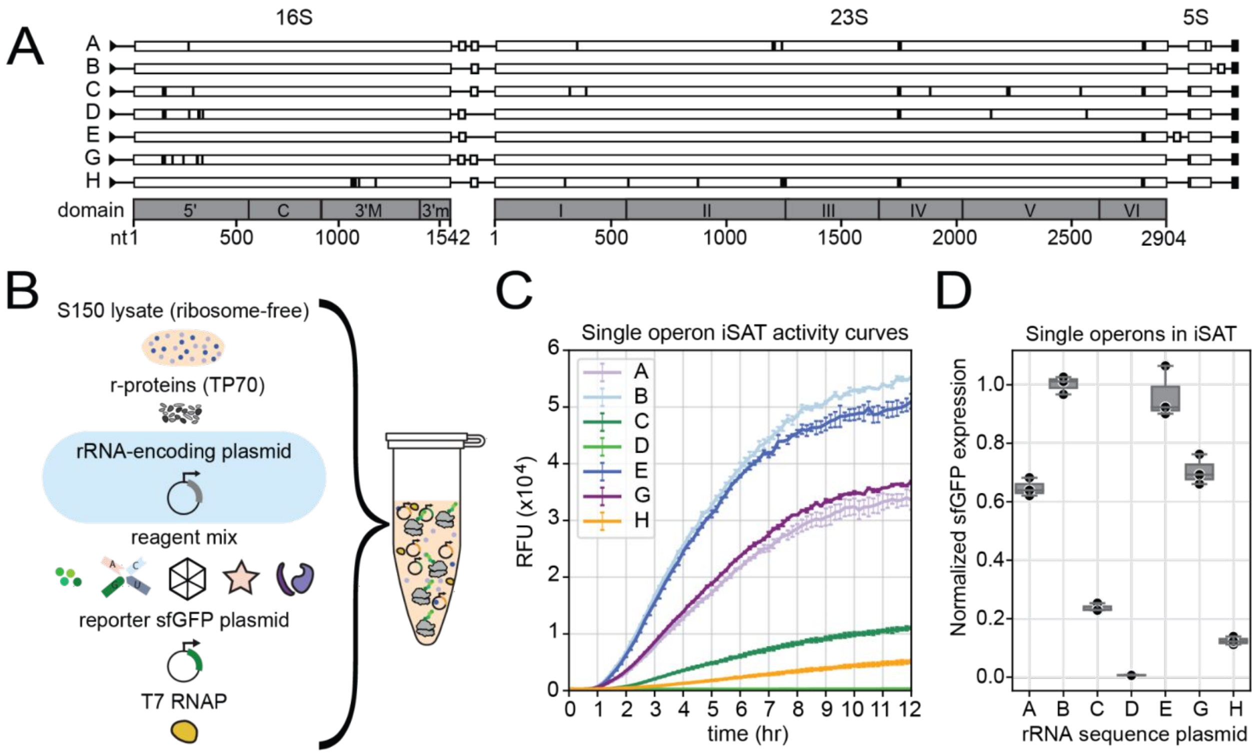

2. Line 5 on page 8, rrnD and H sequences are unable to support life while figure 2B shows rrnH can support strain growth. This is very confusing.

We thank you for raising this point and agree this was confusing. It turns out that we previously made an error in labeling Figure 2B. Operon rrnH indeed cannot support strain growth. We have corrected this mistake by relabeling the figure (noting that the original Figure 2B is now Figure 2C).

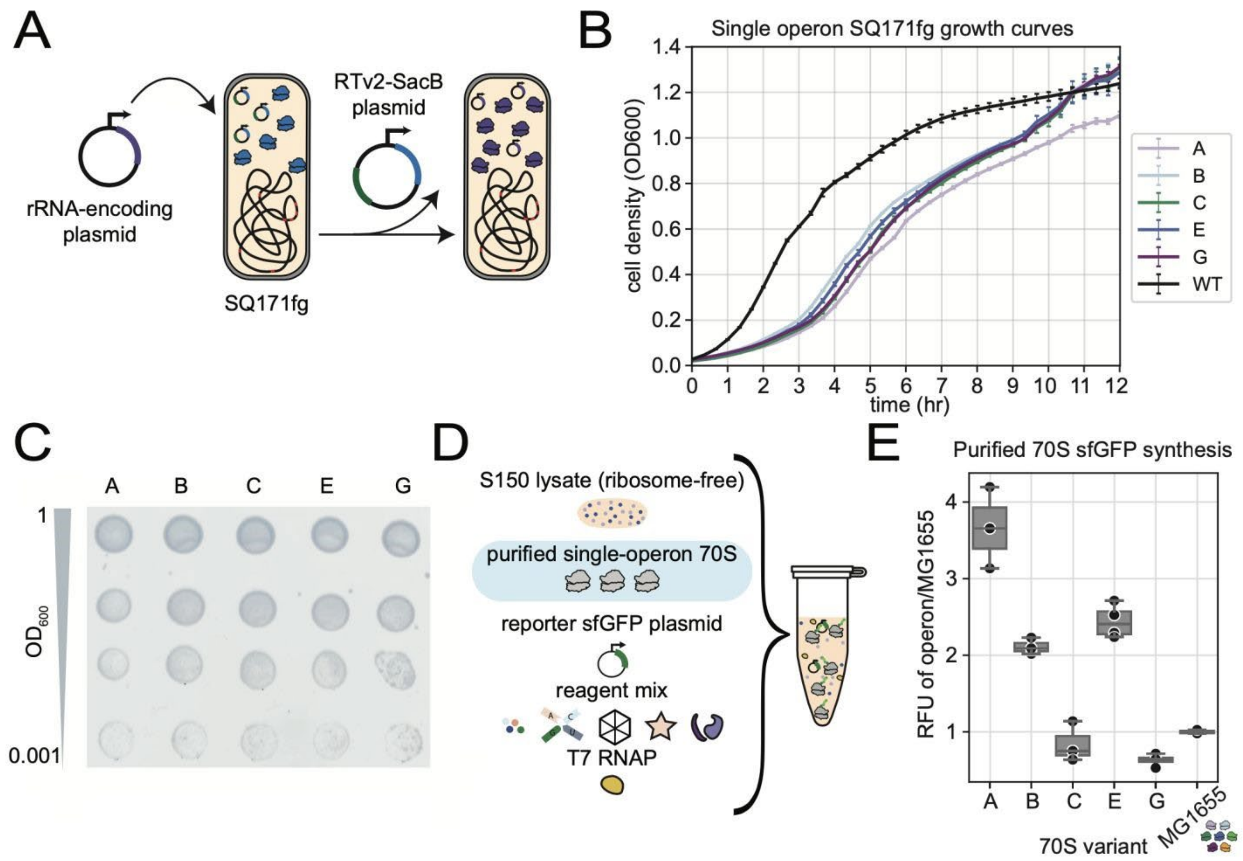

3. In figure 1C and figure 2D, why is the relative activity of different operons inconsistent between the two figures? For example, rrnA and G exhibit similar activities in figure 1C, but in Figure 2D, there is a significant difference in their activities. Given that the reaction conditions are largely the same, why would this lead to such significant variations.

We thank you for asking this question and understand how one might expect the activity trends to be the same. However, there is a nuance that leads to the variation. Specifically, the data in Figure 1C are the output of iSAT reactions, which means they are reporting on the integrated activity of *in vitro* rRNA transcription, ribosome assembly, and translation. The data in Figure 2D (2E in the revised manuscript) is taken from *in vitro* translation reactions with purified ribosomes (i.e., ribosomes expressed and assembled in cells and then purified via ultracentrifugation). Thus, variations emerge as a result ribosomes being assembled in iSAT reactions in Figure 1C and having already been assembled in cells in Figure 2D (now 2E). We have now clarified this in the text.

> *"As expected, we also observed that the activity trends for purified homogeneous ribosome pools (Figure 2E) do not perfectly match the trends seen in iSAT (Figure 1C) (see, for example, operons A and G). This apparent inconsistency is likely a result of assay differences. Whereas iSAT activity reports on combined in vitro rRNA transcription, ribosome assembly, and translation, cell-free reactions with purified ribosomes are only assessing translational activity of ribosomes expressed and assembled in cells and then purified via ultracentrifugation."*

4. Still in figure 2, why is there a significant difference in the activities of rrnA and C, but their impact on cell growth is minimal as shown in figure 2B?

We thank you for bringing up this point and agree that there are differences between activity of a specific rRNA sequence in iSAT and that same ribosome's activity *in vivo.* In the iSAT system, the ribosome is being tasked with producing a single protein product (i.e., sfGFP). However, in the context of cell growth, the ribosome is responsible for expressing thousands of genes, which may lead to the variations observed. We added text acknowledging this difference and explaining potential reasons for the discrepancy:

*"Of note, we see differences between growth trends (**Figure 2B**) and purified ribosome activity (**Figure 2E**), indicating that a strain's growth profile is not necessarily correlated to the protein synthesis capacity of its ribosome pool. For example, strains carrying operon A have similar growth profiles to strains carrying operon C (**Figure 2B**), while the purified A ribosomes produce nearly four-fold higher sfGFP yields when tested in the cell-free translation (**Figure 2E**). Such variations are consistent with previous studies that have shown that activity of a specific rRNA sequence in an in vitro context does not always correlate directly to that same ribosome's activity in vivo [18,19]. These observations could be attributed to differences between testing for protein synthesis activity in simplified, cell-free environments versus assessing a ribosome pool's ability to enable cell proliferation."*

5. Further details on the growth of strains containing single operon need to be characterized such as growth curve. Does higher operon activity correlate with better growth of the strain? Will strains containing only rrnA exhibit increased protein expression yield?

We agree with the reviewer that further strain growth details should be provided. We have performed growth curve experiments to characterize the Squires strains containing single rRNA operons. In the revised manuscript, we have included growth curves in Figure 2B as well as all growth data (e.g., doubling times, lag time) in the Supplement (shown below). This analysis showed that higher activity does not correlate with better growth (see comment 4 and discussion added to the manuscript above). We added the following point to the text:

"While these SQ171fg derived single-operon strains could grow under the laboratory conditions tested, they had slower growth rates and increased lag times as compared wild type strains, such as MG1655 and BL21 Star (DE3) (**Supplementary Table S5).** This can be attributed, in part, to the metabolic burden of plasmid maintenance as has been reported in the literature[26,27]."

Strain	Lag time (hrs)	SD	Doubling Time (hrs)	SD
SQ-AAA	**3.40**	0.14	**1.19**	0.01
SQ-ABB	**3.13**	0.15	**1.08**	0.00
SQ-BBB	**2.78**	0.07	**1.05**	0.00

SQ-BCB	**2.78**	0.04	**1.07**	0.00
WT - BL21(DE3)*	**1.31**	0.02	**0.66**	0.00
SQ-CCC	**3.21**	0.18	**1.00**	0.01
SQ-DBB	**3.24**	0.13	**1.05**	0.01
SQ-EEE	**2.96**	0.07	**1.07**	0.00
SQ-GGG	**3.15**	0.04	**1.13**	0.00
SQ-HBB	**3.24**	0.03	**1.11**	0.00
WT - MG1655	**1.28**	0.02	**0.69**	0.01

Supplementary Table S5: Growth data of all strains included in this study. Lag time and doubling times shown. Data represent mean and standard deviation (SD) of n=7 replicates. Replicates containing outliers that fell >2 standard deviations from the median were excluded from analysis.

6. Most importantly, the effect of using single operon derived ribosomes pools to enhance protein yield is not significant. Is it feasible to design and screen for rRNA sequences with enhanced activity?

Thank you for the opportunity to clarify this point and we apologize that this was unclear. As shown in Figures 4C and 4D, we have designed and shown experiments for rRNA sequences that enhance protein synthesis activity. Specifically, the enhancement in protein yield because of the single operon derived ribosome pool is nearly 2-fold and statistically significant for 5 of the 6 proteins tested in this study. We performed a students' paired t-test with $p<0.05$ and have highlighted figure 4D that four of the five proteins tested in this panel have a statistically significant increase in expression when expressed in lysate expressing the single-operon rRNA. We added the following text to the manuscript:

> *"Of the five proteins tested, four showed a statistically significant increase in yield (as calculated by a student's paired t-test with $p<0.05$)…"*

Additional Questions:
Quality of experimental data, technical rigor: Moderate

Significance to chemistry researchers in this and related fields: Moderate

Broad interest to other researchers: Moderate

Novelty: Moderate

Is this research study suitable for media coverage or a First Reactions (a News & Views piece in the journal)?: No

Reviewer: 3

Recommendation: Publish in ACS Central Science after minor revisions noted.

Comments:
This manuscript describes an investigation of the seven distinct genomic rRNA operons from E. coli MG1655 and the performance of the ribosomes encoded in these operons in supporting protein synthesis. In single-operon iSAT assays, the operons support varying levels of GFP synthesis. Attempts to transform a ribosome-deficient E. coli strain with plasmids encoding one of the operons led to successful transformation and proliferation of 5 out of 7 single operons. GFP synthesis using purified single-operon ribosomes showed that multiple 70s ribosome variants support higher levels of GFP synthesis than the pooled ribosomes from MG1655. Mutations at several helices within the ribosomes were studies systematically and shown to exhibit further variability in the levels of GFP synthesis observed. Cell-free protein synthesis studies with a single operon ribosome led to higher yields of GFP for several variants, and for the AAA operon ribosome, several proteins were expressed at higher levels in comparison to the control MG1655 pool of ribosomes. Additional assays using the single-operon ribosome lysate yielded positive functional readouts, further confirming successful protein synthesis with single operon ribosomes in vitro.

We thank you for this detailed and accurate summary of our work.

Overall, this study is an important study and has implications for biochemistry, synthetic biology, and biomanufacturing. This study is extremely interesting because it indicates that ribosomes encoded by different genomic rRNA operons from E. coli exhibit substantially different behaviors under the conditions studied. This in itself is an important and surprising finding. The additional investigations of single operon ribosome properties both in in vitro protein synthesis assays and in cell spotting assays (to determine viability) are also intriguing. The fact that some single operons substantially outperform the wild-type ribosome pool from MG1655 and that other single operons exhibit no detectable protein synthesis is another important set of observations pointing to a diverse ribosome pool in MG1655. While this is important work, there are some concerns outlined below that the authors should address prior to publication. The concerns range from relatively substantial to relatively minor.

We thank you for highlighting how our study is important for multiple fields and 'extremely interesting.' We, too, were surprised by the results, and have addressed your minor concerns below.

1. Figure 2: Panel B is labeled with operons ABCGH, but panel D is labeled with operons ABCEG, and the text states that strain development for operons D and H was not possible. This discrepancy needs to be resolved.

We thank you for catching this mistake, as both panels B and D should be labeled ABCEG. We have corrected this in the figure.

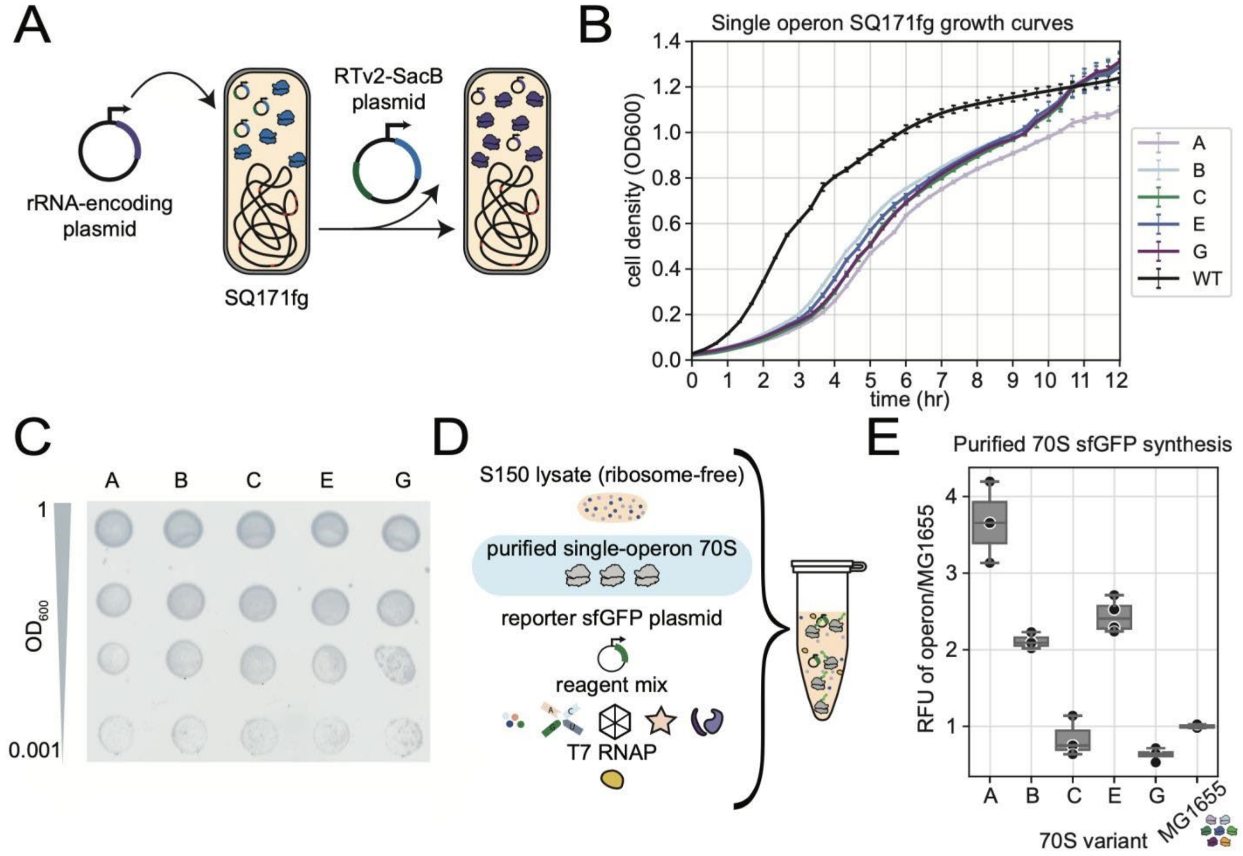

2. Figure 4: The AdhE2 activity quantification results are a bit puzzling. The methods state that the negative control lacks AdhE2 expression, but ~30% conversion of butanol is observed even without the added plasmid encoding the alcohol dehydrogenase. Is this level of conversion expected for the negative control? This needs to be clarified in the text.

We understand your confusion and would like to clarify the result, both here and in the text. In this experiment we expressed the AdhE2 enzyme from *Clostridium acetobutylicum* in our crude cell lysates. The contains numerous native *E. coli* metabolic enzymes. *E. coli* natively has several alcohol dehydrogenases that are known to act on aldehydes [5]. The assay to confirm activity of AdhE2 involved adding butyraldehyde and NADH to the crude lysates with and without AdhE2. Without AdhE2 native alcohol dehydrogenases will act on butyraldehyde. In the presence of AdhE2, we see an increase in conversion to butanol. The relative increase in conversion is used to confirm that AdhE2 is active. Our goal was to test our altered ribosome pool's ability to produce active protein and not to quantitatively evaluate enzymatic conversion of butyraldehyde to butanol. We have clarified this accordingly in the text.

---

[5] Jason T. Ku, Wiwik Simanjuntak, and Ethan I. Lan, "Renewable Synthesis of N-Butyraldehyde from Glucose by Engineered Escherichia Coli," *Biotechnology for Biofuels* 10 (December 2017): 291, doi:10.1186/s13068-017-0978-7.

*"We also confirmed the functionality of the aldehyde-alcohol dehydrogenase (AdhE2) from Clostridium acetobutylicum by measuring conversion of butyraldehyde to butanol in crude AAA lysates with and without AdhE2 expression (**Figure 4F**)[50]. When AdhE2 was expressed in AAA lysate, we measured a net conversion rate of ~35% of butyraldehyde to butanol, matching previously reported values[50]. The butanol yield seen in the negative control (without AdhE2 expression) results from the previously described activity of native E. coli alcohol dehydrogenases which act on butyraldehyde[51]."*

3. "Support life" and related terminology. Laboratory cell growth and protein synthesis conditions are very narrow in relationship to the possible range of conditions that are possible in natural systems (for example, consider biofilm growth versus planktonic growth). It is not yet known whether the operons that show low or zero activity in the experiments performed in this study are similarly inactive in other conditions. Thus, the phrase, "support life" is overly broad and not accurate without additional clarification. Recommend rewording and careful consideration of language surrounding what protein synthesis and cellular proliferation is supported by different variants.

We thank you for this comment and agree that the terminology could be overly broad and would benefit from additional clarification. When we use the phrase "support life," we mean it in the context of a single operon sequence being introduced into the SQ171fg strain and resulting in cell growth under controlled conditions (grown in rich media and at 37°C). We have altered the language to clarify this, replacing "support life" throughout the text with more detailed explanations of the selection performed. For example, with respect to Figure 2, we have replaced "support life" with "unable to independently enable cell growth in the context of the SQ171g strain under controlled laboratory conditions."

4. This study is performed predominantly in vitro, but several new E. coli strains containing only a single rRNA operon were created. It would be interesting to know the growth characteristics of these strains and how they compare to other derivatives of the SQ171 strain. Ideally, doubling times or growth curves of the newly created strains alongside additional SQ171 strain derivatives such as SQ171fg as controls would be an excellent addition to this work (spot assays may also be appropriate). This would augment the quality of this study substantially, especially since the properties of a ribosome that make it good for high-level protein synthesis may not lead to efficient cell proliferation.

We thank you for this suggestion and agree it is an interesting experiment. In the revised manuscript, we have performed growth assays of the new strains alongside control strains, which we now include in Figure 2 as well as in the supplement, where we now have the doubling times and lag times of all the strains shown in this work. As you hypothesized, the properties of a ribosome that make it good for high-level protein synthesis did not lead to more efficient cell proliferation. We have added the following discussion:

*"Operons A, B, C, E, and G were successfully transformed and selected, having similar growth phenotypes when grown in LB medium (**Figure 2B, 2C**). While these SQ171fg derived single-operon strains could grow under the laboratory conditions tested, they had slower growth*

*rates and increased lag times as compared wild type strains, such as MG1655 and BL21 Star (DE3) (**Supplementary Table S5).** This can be attributed, in part, to the metabolic burden of plasmid maintenance as has been reported in the literature[26,27]. Single operon strain development for operons D and H was not successful, as the original SacB plasmid was not able to be cured, indicating that rrnD and H sequences are unable to independently enable cell growth in the context of the SQ171g strain under controlled laboratory conditions."*

5. At the start of the results and discussion, there are two paragraphs of description of the iSAT system. Some of the text of these paragraphs reads like introductory material, especially the description of prior iSAT studies. Recommend considering moving some of this material into the introduction.

As recommended, we moved the text introducing how the iSAT system works and as well as examples of past projects that used this platform into the introduction section.

6. Figure 1C: with every operon in a different shade of green, it is hard to pinpoint which shade corresponds to which specific operon. Recommend using multiple colors to make this easier to understand. The same color scheme could be used in the "single operons" graph to the right, which might clarify things further and make the figure easier to understand.

We thank you for this comment and agree that the different shades of green were difficult to differentiate. We have updated the figure to have higher contrast colors and have updated all figures in the manuscript to match the new coloring.

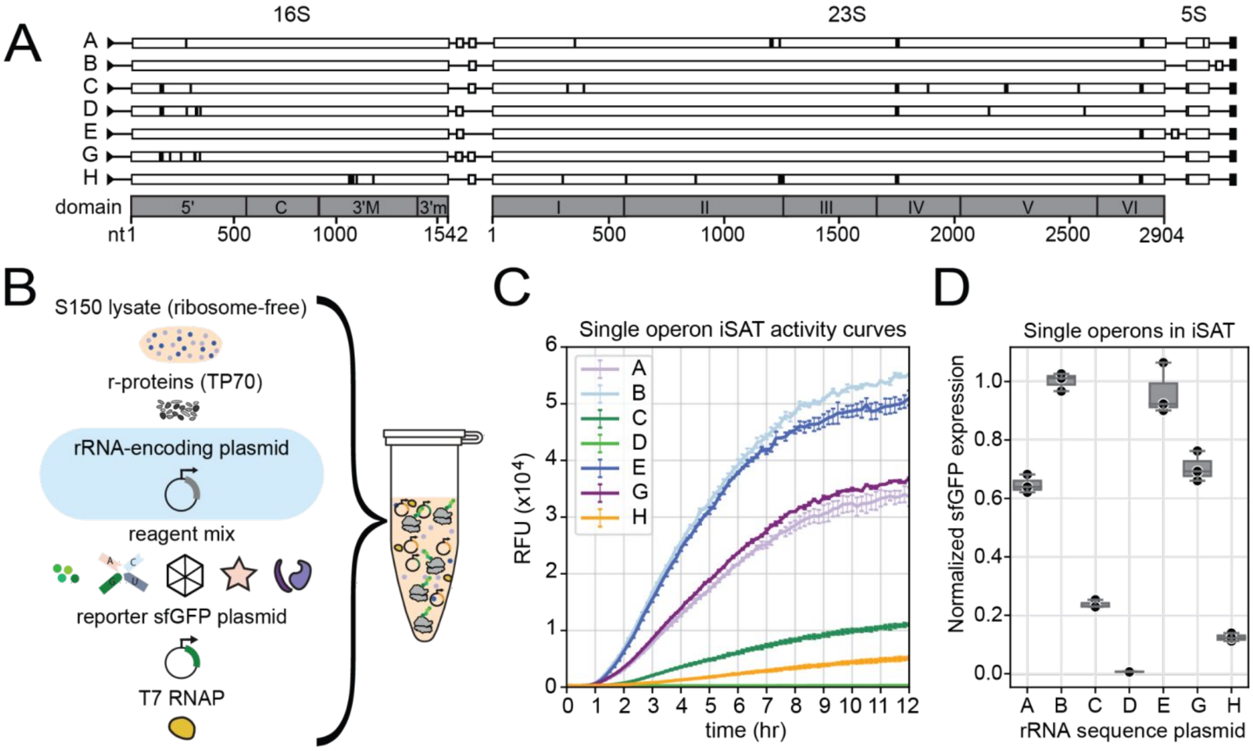

7. Supporting information: there is an unresolved Word comment regarding plasmid pJL1CRM197 and its sequence in relationship to the sequence found in another lab publication. The apparent discrepancy mention in the comment needs to be resolved and clarified.

We thank you for catching this, as there is a difference in the C-terminal tags that we need to clarify. The plasmid map being referred to in the comment is actually "pJL1CRM197-4XDQNAT", whereas the plasmid used in this study is "pJL1-CRM197-ComP". We confirmed that this plasmid is pJL1-CRM197-ComP by sequencing it using services provided by Plasmidsaurus and have corrected the name of the plasmid in the supplement to resolve the issue.

Additional Questions:
Quality of experimental data, technical rigor: High

Significance to chemistry researchers in this and related fields: Top 1%

Broad interest to other researchers: Top 1%

Novelty: Top 1%

Is this research study suitable for media coverage or a First Reactions (a News & Views piece in the journal)?: No

If the Editor has suggested that your manuscript may benefit from language editing, you are encouraged to have it reviewed by a fluent English speaker before resubmitting. ACS Authoring Services can assist you by matching your manuscript with trained chemists who will edit your work for accurate terminology and phrasing. For pricing and information, visit https://authoringservices.acs.org/. Please note that using this (or any) service does not guarantee that your manuscript will be accepted for publication.

oc-2023-01413u.R2

Name: Peer Review Information for "Ribosome pool engineering increases protein biosynthesis yields"

Second Round of Reviewer Comments

Reviewer: 1

Comments to the Author

The authors have addressed all my concerns.

Reviewer: 2

Comments to the Author

I have no more concerns.

Reviewer: 3

Comments to the Author

The thoughtful revisions greatly increased the quality of an already-strong study. I have no further points for consideration.

Author's Response to Peer Review Comments:

Dear Editor,

We are excited to hear that our manuscript has been provisionally accepted for publication in ACS Central Science. Please find enclosed our revised manuscript with the required formatting changes made.

The TOC/Synopsis is now beneath the References along with the SI paragraph, and the Supplementary Information document has been updated to have a Table of Contents section as well as page numbers numbered consecutively starting with S1.

Thank you very much for your time and consideration.

Sincerely,

Camila Kofman, PhD
